# Trust in Group Decisions: a scoping review

**DOI:** 10.1186/s12909-019-1726-4

**Published:** 2019-08-14

**Authors:** Jason E. Sapp, Dario M. Torre, Kelsey L. Larsen, Eric S. Holmboe, Steven J. Durning

**Affiliations:** 1 Department of Medicine, Division of Health Professions Education, Uniformed Services University of the Health Sciences, 4301 Jones Bridge Rd, Bethesda, MD 20814 USA; 2 Milestones Development and Evaluation, Accreditation Council for Graduate Medical, Education, Suite 2000, 401 North Michigan Avenue, Chicago, IL 60611 USA; 3 Department of Medicine, Tripler Army Medical Center, 1 Jarrett White Rd, Tripler AMC, HI 96859 USA

**Keywords:** Trust, Group, Decision, Competency committee

## Abstract

**Background:**

Trust is a critical component of competency committees given their high-stakes decisions. Research from outside of medicine on group trust has not focused on trust in group decisions, and “group trust” has not been clearly defined. The purpose was twofold: to examine the definition of trust in the context of group decisions and to explore what factors may influence trust from the perspective of those who rely on competency committees through a proposed group trust model.

**Methods:**

The authors conducted a literature search of four online databases, seeking articles published on trust in group settings. Reviewers extracted, coded, and analyzed key data including definitions of trust and factors pertaining to group trust.

**Results:**

The authors selected 42 articles for full text review. Although reviewers found multiple general definitions of trust, they were unable to find a clear definition of group trust and propose the following: a group-directed willingness to accept vulnerability to actions of the members based on the expectation that members will perform a particular action important to the group, encompassing social exchange, collective perceptions, and interpersonal trust. Additionally, the authors propose a model encompassing individual level factors (trustor and trustee), interpersonal interactions, group level factors (structure and processes), and environmental factors.

**Conclusions:**

Higher degrees of trust at the individual and group levels have been associated with attitudinal and performance outcomes, such as quality of group decisions. Developing a deeper understanding of trust in competency committees may help these committees implement more effective and meaningful processes to make collective decisions.

**Electronic supplementary material:**

The online version of this article (10.1186/s12909-019-1726-4) contains supplementary material, which is available to authorized users.

## Background

Training competent physicians is, in many ways, a matter of trust. Patients trust providers to competently address their ailments and assist in helping them achieve good health. Accreditation organizations trust that graduate medical education (GME) programs implement policies and procedures to prepare graduates to provide highly competent medical care. GME programs that utilize competency committees trust these groups to make accurate decisions regarding trainee progress. Trust as a core concept is growing in prevalence in the broader education literature and more recently has been discussed in health professions education, but is the nature of trust in each of these contexts the same?

Health professions education has examined trust in the context of Entrustable Professional Activities (EPAs), which include decisions that supervisors make about the appropriate level of trainee supervision for different clinical responsibilities [1, 2]. While such individual decisions of trust are important, group decisions regarding trainee competence are critical. In some countries, competency committees are an essential part of the postgraduate medical education program. These committees usually consist of training program faculty who collectively determine which trainees are on appropriate paths towards unsupervised clinical practice and which are not. They assess each trainee’s progress, and they make recommendations to program directors regarding trainee overall progress, promotion, and remediation [3–5]. Given the high-stakes decisions shouldered by competency committees, trust is a critical component of these group decisions.

Although some recent studies describe data sources that competency committees use and how this information may impact their collective decisions [6, 7], health professions education research has not studied trust within these committees and trust in committee decisions from individuals either within or outside the group. Scholars outside of medicine have explored trust at the individual level and also at the group level (e.g. in work teams) [8–10]. Even within a group, research suggests that trust occurs at multiple levels encompassing both individual level and group level processes simultaneously [8]. For purposes of this review, we define a group as “a collection of individuals who have regular contact and frequent interaction, mutual influence, common feeling of camaraderie, and who work together to achieve a common set of goals.” [11]

Research on group trust has focused primarily on trust within a group (e.g. as an aggregate or combination of individual trust) as opposed to trust in group decisions from individuals either within or outside of the group. However, “group trust” in any of these contexts has not been clearly defined. Defining trust in group settings is important to informing and potentially improving how competency committees and other groups make collective decisions; proposing a model illustrating various components of group trust may also help such committees implement a more effective and meaningful decision process regarding trainee competence.

The purpose of this scoping review is to examine the definition of trust in the context of group decisions and propose a definition of group trust. Additionally, we examine what factors may influence group trust through a proposed model to illustrate the relationship between individual level and group level trust.

### Theoretical framework

In order for decision-making groups to achieve consensus, communication and interactions between people, objects (i.e. items that help augment human capabilities in a learning or task-oriented setting, such as learning management systems), and the environment are believed to be important. We therefore chose situated cognition as our theoretical framework to support the development of a model and interpretation of the literature, helping to advance our understanding of group processes in the context of competency committees. Situated cognition proposes that thinking and learning are situated (or located) within the larger social and physical context of the environment [12]. This theory suggests that a group setting is highly complex with multiple components (i.e. physical, social, and cultural) and opportunities for interactions between these components. It recognizes the complex relationship between participants, objects (i.e. artifacts) that help to augment individuals’ cognitive capabilities, and the environment. Situated cognition emphasizes how these various interactions lead to thinking, learning, and decisions, and the model we propose in this review is built upon all of these interactions.

## Methods

We chose a scoping review to understand how trust operates in groups making collective decisions and to support the development of a model using situated cognition that can be tested empirically in future studies. This scoping review was conducted based upon the methodology originally outlined by Arksey and O’Malley and expanded upon by Levac et al. [13, 14] We initially reviewed the broad literature on trust at the individual, group, and organizational levels. During this initial review, we identified a multilevel model of trust in work teams from outside of health professions education that we used as a framework to guide our subsequent search and analysis [8]. Recognizing that the medical literature is narrow for this particular subject and the potential benefits of including what is known about trust in group settings from other fields, we chose to expand our search to include other disciplines that involve similar groups (e.g. committees and juries).

With the assistance of a medical librarian, we conducted a search in MEDLINE, ERIC, PsycINFO, and SCOPUS in April 2018 (our specific search strategy is shown in Supplemental Digital Appendix). We did not limit the publication dates of our searches, but the results were limited to English-language articles.

The research team reviewed titles and abstracts using the following initial inclusion criteria: original research or review articles; “trust” referenced in the title; and articles that included information about a group, team, committee, or similar collection of individuals. Articles were excluded if they did not meet the inclusion criteria above. We also excluded articles for the following reasons: the word “trust” was used in a different context (e.g. trust fund); trust was a narrow focus of the article; no clear link to group processes or dynamics; the link between trust and a group was not clear; the article referenced only virtual groups; and articles from the computer science, information technology, and economics fields. Articles were excluded from these three fields because we felt that the context in which they utilized trust was not applicable to decision-making groups. The research team met regularly and came to complete consensus during this step of the process.

**Fig. 1:**
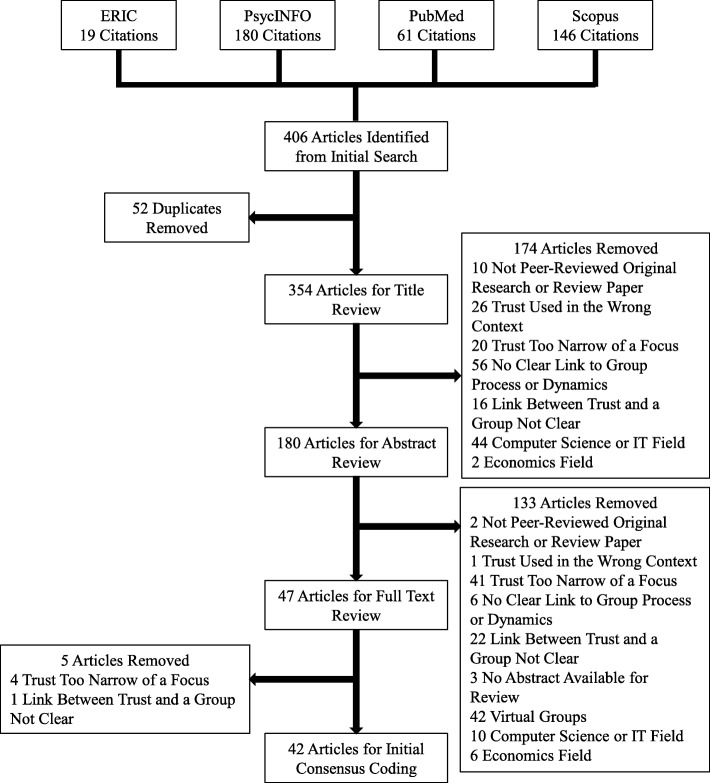
Flowchart of inclusion/exclusion determinations

A data extraction sheet was modeled after a sensitizing framework in a recently published review of trust in teams outside of medicine and our initial review of the trust literature (See Additional file 1) [8]. We extracted definitions of trust along with factors pertaining to trust at the group level and synthesized this information into our primary outcomes: a definition of group trust, factors that influence group trust, and a proposed model to illustrate the relationship between trust at the individual and group levels. Each article was independently coded by two members of the research team. Discrepancies in coding were resolved in regular in-person research team meetings. Figure 1 outlines the review process.

Consistent with revised scoping review methodology recommendations [14], we obtained consultation from two independent medical educators outside of our research team who have experience with competency committees. We presented them our preliminary findings, and we incorporated their feedback in our final revision of the results.

## Results

We identified 406 publications and included 42 articles. The two main reasons for exclusion were that the articles demonstrated no clear link to group dynamics and/or trust was too narrow of a focus (Fig. 1). A complete list of the 42 included publications is available in Additional file 2. Additionally, a complete list of the excluded publications and the reasons for exclusion are available in Additional files 3, 4, 5, and 6.

### Publication characteristics

Publications were written by 108 unique authors, with 103 (95.4%) contributing once. Twelve countries were represented, and six (14.2%) publications were multi-national collaborations. Five (11.9%) publications involved healthcare work teams. Thirteen (31%) involved undergraduate or graduate students, though none of the included publications focused on health professions education (Table 1).

**Table 1:** Demographics Of Manuscripts On Trust And Groups

Characteristic	n (%)
Country of Origin	
United States	17 (40.4%)
Netherlands	10 (23.8%)
Australia	3 (7.1%)
United Kingdom	2 (4.7%)
Canada	2 (4.7%)
Finland	2 (4.7%)
Denmark, France, Israel, Singapore, Spain, United Arab Emirates	1 each (2.4%)
Study Population	
Work Teams	19 (45.2%)
Multiple Industries	7 (16.7%)
Healthcare	3 (7.1%)
Education	2 (4.7%)
Finance	2 (4.7%)
Social Care	2 (4.7%)
Manufacturing	2 (4.7%)
Hospitality	1 (2.4%)
Not Specified	10 (23.8%)
Undergraduate Students	7 (16.7%)
Graduate Students	6 (14.2%)

### Publication types

Thirty-four (81%) publications were empirical research studies, seven (16.7%) were literature reviews, and one (2.4%) was a meta-analysis. Of the empirical publications, all were prospective studies; 85.3% used quantitative methods (*n* = 29: surveys = 27, scenarios = 2), while the remaining utilized qualitative (n = 2) or mixed (*n* = 3) methods.

### Definitions of trust

The most often cited general definition of trust by articles in this review (*n* = 28, 66.7%; almost always referring to trust at the individual level) is the “willingness of a party to be *vulnerable* to the actions of another party based on the *[positive] expectation* that the other will perform a particular action important to the trustor, irrespective of the ability to monitor or control that other party.” [15] Other general definitions of trust cited by articles in this study are provided in Table 2. Positive expectations are beliefs that the actions of another will be beneficial, favorable, or at least not detrimental [26]. Vulnerability implies that there is something of importance to be lost and involves taking a risk; indeed, risk is also a commonly cited component of trust [17, 18, 21, 22, 24, 27–33]. Thus, because trust in general can be defined as a willingness to be vulnerable to the actions of another party, trust at any level (e.g. individual or group) can arguably be characterized as a measure of how much risk an individual is willing to incur in relationships.

**Table 2:** Definitions Of Trust

“The willingness of a party to be vulnerable to the actions of another party based on the [positive] expectation that the other will perform a particular action important to the trustor, irrespective of the ability to monitor or control that other party.” [15]	
“The degree of confidence the members of a team have in the goodwill of its leader, specifically, the extent to which they believe that the leader is honest, sincere, and unbiased in taking their positions into account.” [16]	
“A psychological state that manifests itself in the behaviors towards others, is based on the expectations made upon behaviors of these others, and on the perceived motives and intentions in situations entailing risk for the relationship with those others.” [17]	
“Trust in individuals is an expectation or belief that actions from another party will be motivated by good intentions. Moreover, individuals take a risk in this belief because the other party may not act out of benevolence.” [18]	
“Expectations, assumptions, or beliefs about the likelihood that another’s future actions will be beneficial, favorable, or at least not detrimental to one’s interests.” [19]	
“A belief (held by an individual or a group) that another individual or group (a) makes good-faith efforts to behave in accordance with any commitments made both explicitly or implicitly, (b) is honest in whatever interactions preceded such commitments, and (c) would not take excessive advantage of another even when the opportunity became available.” [20]	
“The extent to which a person is confident in, and willing to act on the basis of, the words, actions, and decisions of another. Critically, trust requires the presence of uncertainty and risk.” [21]	
“One party’s (the trustor) confident expectation that another party (the trustee), on whom the trustor must rely, will help the trustor reach his or her goals in an environment of risk and uncertainty.” [22]	
“The optimistic acceptance of a vulnerable situation in which the truster believes the trustee will care for the truster’s interests through the bracketing out of uncertainty. Empirically, we therefore delineate trust in terms of the overlooking of uncertainty and possible negative outcomes based on considerations of the motives and/or competencies of others.” [23]	
“A resolve to bear an experienced risk by confiding in the new and unknown. It goes beyond the blind, unquestioning faith that an infant may have of a parent because there is an acknowledgement of past experience that denotes trust as a risk, especially where there are unknown elements. It is a decision to act in concert with others in anticipation of a return. By placing trust in others, we place ourselves in a position of vulnerability, ceding power. Trust given in the anticipation of reciprocity creates a sense of mutual obligation.” [24]	
“The generalized expectation of predictable and benevolent motives and/or behavior from others.” [25]	

Within a group, trust has been proposed by fields outside of medicine (e.g. management, organizational and social psychology, and higher education) to exist at both the individual and group levels of analysis and that conceptualizations of group trust should include individual trust. However, we were unable to find in this review a clear definition of “group trust.”

### Group trust definition

**Fig. 2:**
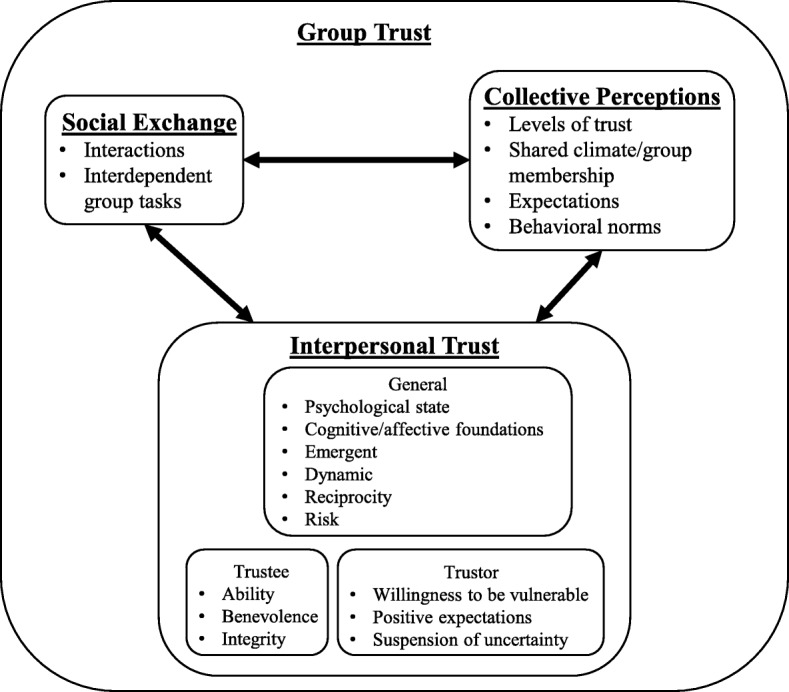
Definition of Group Trust

We propose the following definition of group trust: “a group-directed willingness to accept vulnerability to actions of the members based on the expectation that members will perform a particular action important to the group, encompassing social exchange, collective perceptions, and interpersonal trust.” Fig. 2 outlines the relationship between individual trust and various characterizations of group trust in the literature, which include the three broad categories mentioned in our definition: individual (sometimes referred to as interpersonal) trust, social exchange, and collective perceptions. At the individual level, interpersonal trust occurs in dyadic relationships between members in the group, encompassing general features of trust outlined above along with characteristics of both the trustor and the trustee. One important difference between individual and group trust is that instead of existing in dyadic relationships, group trust implies that these components of trust are shared *among* group members [8]. Scholars note that group trust involves each dyadic relationship within the group along with collective perceptions of trust about the group as a distinct unit [34]. Social exchange, which encompasses individual interactions and interdependent group tasks, is thought to help individuals develop shared perceptions, expectations, and behavioral norms with other group members [35–37]. Moreover, trust at the group level is also felt to be a matter of shared group membership where individuals may be willing to trust other group members to show that the group is important to them and a meaningful part of their identity [38].

### Proposed model

**Fig. 3:**
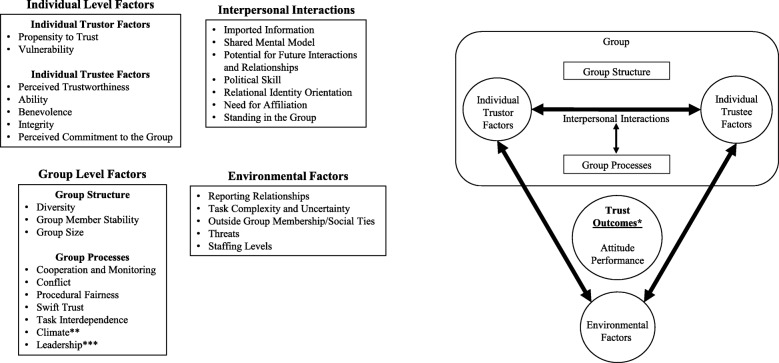
Proposed Group Trust Model. *In certain cases, trust outcomes become inputs into the ongoing system. **Components of a group’s climate include a common group identity, group efficacy, psychological safety, psychological collectivism, and psychological ownership. ***Group leadership includes leadership style, characteristics, and behaviors

The second aim of our study is to explore specific components of groups that may influence how trust can be conceived to work in health professions education group settings, such as competency committees. We propose a preliminary model (Fig. 3) for various elements of trust pertaining to group decisions. Our model includes individual level factors (encompassing both the trustor and the trustee), group level factors (encompassing both group structure and group processes), environmental factors, and importantly interpersonal interactions, which are a key element in situated cognition and have been cited as crucial for competency committees [39]. Below we outline some of the factors in our proposed model (See corresponding Table 3 for a complete description of all the factors, which includes potential implications for competency committees).

**Table 3:** Results

Theme	Sub-Theme	Definition/ Implications for Trust Model	Implication for Competency Committees
Individual-Level Factors: Trustor Factors	Propensity to Trust [15, 17, 18, 28, 33, 40]	A general willingness to trust others that varies with people’s different experiences, personality types, and cultures	Committee members have different propensities to trust other members and the process that the committee uses to make collective decisions. A large degree of propensity to trust asymmetry may adversely affect group level trust.
	Vulnerability [15, 23]	Exposure to the possibility of being harmed, either physically or emotionally	Committee members with higher levels of trust toward other individuals or the group are willing to accept more personal vulnerability than less trusting individuals.
Individual-Level Factors: Trustee Factors	Perceived Trustworthiness [15]	Characteristics and actions of an individual or group that help explain why some parties are more trusted than others	At the individual level, a committee member’s ability, benevolence, and integrity contribute to his/her perceived trustworthiness by other members, which determines how much he/she will be trusted within the group. Collectively at the group level one may also judge these factors from an external perspective (e.g. the training program, patients, etc) to determine the perceived trustworthiness of competency committee decisions and the group itself.
	Ability [15, 41]	A group of skills and competencies that enable an individual to have influence within some specific domain	
	Benevolence [15]	The extent to which a trustee is believed to want to do good to those individuals on the giving end of a trusting relationship (i.e. trustors)	
	Integrity [15]	The trustor’s perception that the trustee adheres to a set of principles that the trustor finds acceptable	
	Perceived Commitment to the Group [41]	Established group members are more likely to trust new members who seem more committed to the group	A committee member’s commitment to the group and how that commitment is perceived within the group may influence how much that individual is trusted by other members.
Individual-Level Factors: Interpersonal Interactions	Imported Information [40]	Preexisting knowledge, stereotypes, and preconceptions stored in group members’ memories	Committee members bring their own personal experiences regarding individual trainees to the committee. This information forms the basis for discussions regarding trainee competence and attainment of specific educational milestones.
	Shared Mental Model [42, 43]	The shared knowledge and organized understanding of the information and resources used by the group, tasks and problems faced by the group, and individual group members’ imported information	Developing a shared model of trainee competence is critical for these committee decisions. Sources of shared information include written evaluations, results of knowledge exams, conversations amongst faculty members with their own experiences with the trainees, etc.
	Potential for Future Interactions and Relationships [33]	A person’s early feelings about group members can create a sense of optimism about the potential of the relationship	New committee members may be more willing to cooperate with other members given the potential of building stronger future relationships.
	Political Skill [44]	A social effectiveness construct defined as the ability to effectively understand others at work and to use such knowledge to influence others to act in ways that enhance one’s personal and/or organizational objectives	A committee member who has particularly strong opinions about a trainee may be more persuasive (and potentially trusted as well) based upon his or her degree of political skill.
	Relational Identity Orientation [45]	One’s conception of his/her relatedness to other individuals	Committee members with a high relational identity orientation may take great steps to build relationships within the group and will likely be more trusted as well.
	Need for Affiliation [33]	An overlapping common factor among group members that motivates them to view each other as trustworthy	This collective need helps a committee build a shared level of trust amongst members and within the group.
	Standing in the Group [38]	A person’s inclusion or membership in a group (e.g. power or hierarchy differentials)	An individual’s position, whether specified (e.g. the committee chair) or unspecified (e.g. based upon seniority, rank, job title outside the committee, etc.) may affect how other members interact with this person. Large perceived differences in this category may adversely affect interactions and therefore trust.
Group Level Factors: Group Structure	Diversity [21, 22, 34, 40, 42, 45]	While cultural diversity has positive and negative influences on group trust, skill diversity has been shown to positively impact intragroup trust and group performance	Members of the committee should have a number of shared traits given that they are associated with the healthcare field and educating trainees, thus cultural diversity should have a smaller influence on these committee decisions. Diversity of opinions and skill diversity are likely beneficial to committee decisions, leading to higher levels of trust.
	Group Member Stability [42]	Positively impacts communication patterns, the social interaction of teams, and interpersonal trust	Committee member stability is important, especially if faculty members are assigned specific trainees to follow longitudinally.
	Group Size [25]	Smaller groups have been shown to be more trustworthy than larger groups	While small groups are perceived to be more trustworthy from an external perspective, training programs need to weigh factors such as the number of trainees and faculty availability when deciding the optimal size of their competency committee(s).
Group Level Factors: Group Processes	Cooperation and Monitoring [8, 17, 28, 33, 46]	Group members who experience high trust tend to cooperate, share information, accept influences from others, and feel personally involved with the group. Monitoring behaviors usually occur when trust between members is low and manifest when members feel a need to be vigilant about the actions and intentions of others.	Trust between committee members will most likely lead to increased cooperation. If trust is low, committee members may increase their monitoring behaviors, which detracts from the collective decision-making process and likely affects the quality of these committee decisions.
	Conflict [26, 31–33, 43, 47, 48]	One of the most important factors influencing group trust. Task conflict is generally felt to be functional while relationship conflict may be dysfunctional. Higher levels of either type of conflict usually lead to diminished group trust	To a certain extent, task conflict in a competency committee may lead to better decisions. When faculty members have different opinions about trainees, the discussion may be livelier. As long as the discussion doesn’t get hijacked by those with strong opinions or degenerate into relationship conflict, variable faculty member opinions and experiences with trainees likely leads to better group decisions.
	Procedural Fairness [16, 42, 49, 50]	For decision-making groups, this includes consideration of member input and influence over a decision. Fairness of procedures used by a group has been associated with increased levels of trust and commitment to group decisions.	Committee decisions are likely to be more trustworthy when the group follows procedures that are judged as fair by faculty members and trainees.
	Swift Trust [33, 40]	Occurs when individuals decide to trust others prior to developing longer term relationships. Swift trust develops as a function of members’ own dispositional tendencies, quickly discernable surface-level cues of others, and imported trust-related information.	Initial/swift trust usually occurs in newly formed committees or with new members (especially if they don’t work with more established members in other contexts). These individuals will have to decide if they are going to trust other members of the group and the committee itself before developing deeper relationships.
	Task Interdependence [8, 34]	The degree to which group members rely on one another and interact in order for the group to accomplish its goals, which may have an effect on group trust and trust between group members	Making collective decisions about trainee competence relies on the “wisdom of the group.” Each faculty member only has a portion of the data based upon their own experiences. The group must compile performance data and members’ experiences to build a collective picture of individual trainee competence.
	Climate [36, 41–45, 51, 52]	Components include a common group identity, group efficacy, psychological safety, psychological collectivism, and psychological ownership	The internal climate of a committee may be influenced by each of these factors. A common group identity, group efficacy, and psychological safety are probably most important for competency committees.
	Leadership [23, 37, 38, 40, 42, 43, 45, 53, 54]	Components include leadership style, characteristics, and behaviors	The committee chair is responsible for implementing the training program’s policies and procedures related to this committee. Committee chairs may be more effective at building individual and collective trust if they are prototypical of the group, utilize an authentic or transformational leadership style, create a safe atmosphere, manage conflict/discussions, and demonstrate relational behaviors.
Environmental Factors	Reporting Relationships [54]	In settings where groups report to diverse outside entities, these reporting relationships can be associated with role conflict, confusing expectations or role ambiguity, excessive demands, and competing priorities	The committee reports to their respective training program, which in turn reports information about resident performance to various accreditation agencies. If a committee is required to report to another entity outside of this chain, it may decrease trust amongst members of the group, especially given the sensitive nature of trainee performance information.
	Task Complexity and Uncertainty [40, 41]	Associated with decreased levels of trust within a group, especially if the group is newly formed	For a competency committee, it may be challenging to determine the attainment of various clinical competence domains for all trainees, especially with gaps in evaluation data or for large training programs. Increasing complexity and uncertainty may adversely affect trust by members in the committee’s processes and decisions.
	Outside Group Membership/ Social Ties [41]	Group members may be worried that membership in other groups can lead new members to act in ways that harm their own group.	Committee members who serve on other hospital committees (e.g. credentials, risk management, etc.) may be perceived as more or less trustworthy from other members. However, this is probably a small contributor to trust within these committees.
	Threats [41]	External dangers or conflict with another group	Time pressures and faculty availability (either for individual meetings or to serve longitudinally on the committee) are potential threats to group level trust within a competency committee. However, an understaffed committee may lead to higher trust between group members.
	Staffing Levels [41]	Understaffed groups may develop stronger trust amongst members than larger groups	
Trust Outcomes	Attitudes [22–24, 27, 33, 36, 40]	May include confidence, resiliency, creativity, group satisfaction and identity, cooperation, and commitment	Individual and group level trust within competency committees may lead to greater cooperation, commitment to the group, satisfaction, and confidence in group decisions.
	Performance [22, 40, 42, 43, 55]	May include group performance, group learning, information sharing, and decision quality	Individual and group level trust within competency committees may lead to better sharing of information, group learning, and quality of decisions.

#### Individual level factors

**Individual trustor factors**

Trust between two group members requires looking at one individual as the “trustor” and the other as the “trustee,” noting this relationship can be reciprocal with the roles shifting between members. Trustor factors include vulnerability and an individual’s propensity to trust, which is believed to vary with people’s different experiences, personality types, and cultures [15]. Showing vulnerability has been shown to be crucial for building trusting group relationships as this demonstrates a willingness to trust other group members and the group itself [23].

**Individual trustee factors**

The three trustee characteristics most often cited in the literature are ability (a group of skills and competencies in a specific domain), benevolence (the extent to which a trustee is believed to want to do good to a trustor on the giving end of a trusting relationship), and integrity (the trustor’s perception that the trustee adheres to a set of principles that the trustor finds acceptable) [15]. In a collective setting, perceived commitment to the group has also been cited as an important individual trait [41].

#### Interpersonal interactions

Interpersonal interactions are an important component of situated cognition, and effective interactions can lead to improved social exchange and cohesion, both of which have been shown to enhance the development of interpersonal trust, especially in new product development teams and technology-supported decision-making groups [21, 42]. For decision-making groups and top management teams, a shared mental model is important and may be conceptualized as the shared knowledge and organized understanding of the information and resources used by the group, tasks and problems faced by the group, and individual group members’ imported information (the preexisting knowledge, stereotypes, preconceptions, and experiences stored in group members’ memories) [40, 43]. The idea of a shared mental model is a critical component of competency committees. While trust between group members is usually felt to enhance information exchange and establish shared understanding, knowledge brought in by new members may adversely affect group collective beliefs and memory structures, especially in well-established groups [42]. Notably, trust can more easily be violated in relationships of shorter rather than longer duration because parties in newly formed relationships are less likely to have had the opportunity to develop mutual understanding and respect [33]. Additionally, a person’s standing in the group, which refers to a person’s inclusion or membership status within a group, may influence how more senior or experienced members interact with newer ones [38].

#### Group level factors

**Group structure**

Regarding a group’s composition, diversity amongst members can have multiple influences on trust within the group. Diversity in this context includes both cultural and skill diversity. In undergraduate psychology students, trust was demonstrated to more likely develop in groups when members perceive each other to have similar attitudes, personality, and intellect than in dissimilar groups [22]. Cultural diversity may, in some contexts, negatively impact group performance and communication effectiveness [40]. However, in contrast to cultural diversity, skill diversity has been shown in an applied psychology meta-analysis to positively impact intrateam trust and team performance [34].

Group member stability and group size are also believed to contribute to a group’s structure. In general, group member stability tends to positively impact communication patterns, the social interaction of teams, and interpersonal trust [42]. From an external perspective, smaller groups (i.e. less than 10 individuals) have generally been shown to be more trustworthy than larger groups (i.e. 10 or more individuals) in undergraduate psychology students [25].

**Group processes**

***Group cooperation and monitoring***

Work outside of medicine suggests that cooperation and monitoring behaviors are commonly impacted by the level of trust within a group. Group members who experience high trust tend to cooperate more, share information, accept influences from other members, and feel personally involved with the group. Monitoring behaviors usually occurs at the opposite end of the spectrum when trust between members is low and manifest when members feel a need to be vigilant about the actions and intentions of others. Groups in which members perceive colleagues as trustworthy are likely to demonstrate fewer monitoring behaviors and more cooperative behaviors based upon psychology research on undergraduate students, teams from European social care institutions, and hospital employees [17, 28, 46].

***Group conflict***

Conflict amongst group members appears to have complex associations. Task (cognitive) conflict tends to arise from individual differences in viewpoints, ideas, or opinions when group members participate in a shared undertaking. Conversely, relationship (affective) conflict tends to arise from interpersonal tensions. Scholars argue that these two types of conflict lead to different outcomes and contend that task conflict can be constructive whereas relationship conflict is usually dysfunctional based upon research from strategic decision-making teams, MBA graduate students, and higher education faculty [26, 31, 32]. Higher levels of conflict (especially relationship conflict) in teams are usually associated with lower trust. On the other hand, too much trust within a group may result in almost no conflict whatsoever regardless of type. Thus, the group may lose out on the positive effects of task conflict [47].

In the management literature, even perceptions of conflict may lead to conflict within a group. How one perceives, defines, and interprets interpersonal discord may be more important than the nature of the conflict itself [33]. When one person distrusts another, that person may interpret ambiguous behaviors as threatening and convey distrust through his or her conduct. The person whose behavior is interpreted as threatening may reciprocate that distrust.

***Group procedural fairness***

Two aspects of decision-making procedures have been shown to create perceptions of fairness: consideration of member input and influence over a decision. Consideration of input refers to the extent a group leader listens to and considers group member input during a decision-making process. Influence refers to the extent group member input affects or is reflected in the final decision. Fair procedures have been associated with positive attitudes toward the group and leader, resulting in group harmony and trust in the leader. Individual members who perceive fair procedures within the group are believed to be more likely to commit to group decisions based upon research from Fortune 500 management teams and Netherlands undergraduate students [16, 49].

***Group task interdependence***

When task interdependence (the degree to which group members must interact and rely on one another to accomplish goals) is high, trust plays a more influential role because teamwork interactions become critical to achieving group goals [8, 34].

***Group climate***

A group’s climate has multiple components and can be defined as shared perceptions of the kinds of behaviors, practices, and procedures that are supported within a group [56]. Climate may include a group’s identity, defined as the degree to which members view the group as “we” versus “I.” A common group identity may help develop trust, especially in diverse work groups, decrease negative attitudes and increase more positive affective responses toward other group members, and reduce mutual tensions and uncertainty [45]. Related to group identity is group efficacy. Efficacy has been defined in undergraduate Canadian business students at either the individual or group level and refers to an entity’s belief in its ability to succeed in specific situations or accomplish a task [44].

Psychological safety, collectivism, and ownership are all distinct and also contribute to a group’s climate. In graduate-level business students, psychologically safe group environments encourage authenticity and risk-taking among group members because there is minimal fear of reprisal, punishment, harassment, or ridicule from others [36]. Groups with higher levels of psychological safety tend to have higher levels of trust [41]. Psychological collectivism refers to a person’s propensity to favor group affiliation and the collective effort of the group over independent and autonomous effort. Compared with members who have low levels of psychological collectivism, a highly collectivistic individual will likely experience greater satisfaction working in a low-trust group environment, be more willing to work with other members, and more readily identify with that group [36]. Finally, psychological ownership refers to a feeling of possessiveness and attachment to a variety of objects within one’s organization. These feelings of ownership lead one to value and take responsibility for those objects. In various industries, individuals who feel ownership may engage in territorial behaviors to communicate and defend their ownership claims, especially in lower trust environments [52].

***Group leadership***

Leaders are believed to play a critical role in the development and maintenance of trust within a group. In healthcare and bank employees, authentic and transformational leadership styles have been shown to improve trust amongst members of a group [37, 53]. Legitimacy (e.g. honesty, fairness, respectfulness) of a group leader may also promote a climate of interpersonal trust in groups [42]. Additionally, prototypical leaders, which are leaders who share essential characteristics of their group, may foster employee trust in coworkers [38].

Leader behaviors may also influence trust. Relational behaviors, those that encourage collaboration and open communication, have been shown to augment trust in top management teams [43]. Other leader behaviors that may foster trust include fairness, particularly for a prototypical group leader [38], the ability to create a safe atmosphere [23], establishing behavioral norms and climate of the group [40], developing unique personal relationships with each group member [45], managing conflict, and creating a superordinate mission for the group [54].

#### Environment factors

Environmental factors also play an important role in situated cognition and may influence trust within a group. External reporting relationships can add complexity to shared tasks. Groups that experience large amounts of pressure and conflict caused by reporting relationships to outside entities may report role conflict, confusing expectations or role ambiguity, and excessive demands resulting in overload and competing priorities [54]. Task complexity and uncertainty can also influence trust amongst a group. The link between task uncertainty and trust has been studied in swift action starting teams, which are highly interdependent teams of skilled individuals in organizations that complete demanding, complex, time-pressured projects and are often formed quickly. High levels of task uncertainty may deplete cognitive resources, so members in these teams may become overly dependent on heuristic-based decision-making and rely more heavily on predispositions than would groups in less uncertain situations [40]. Additionally, group members may value predictability within the group, especially with increasingly unpredictable events outside of the group. As a result, established members of a group might be reluctant to trust new members given the uncertainty that they bring to the group [41]. Additional external factors that may impact a group include individuals’ memberships in other groups and social ties, threats (e.g. external dangers or conflict with another group), and staffing levels (e.g. if a group is understaffed, trust may develop more strongly than if the group were larger because members are forced to work more closely together) [41].

## Discussion

Although multiple definitions of general trust exist in the literature and scholars have explored trust within work teams, we were unable to find a clear definition of “group trust.” Further, trust in group decisions has not been well studied within or outside of health professions education. We define group trust as a group-directed willingness to accept vulnerability to actions of the members based on the expectation that members will perform a particular action important to the group, encompassing social exchange, collective perceptions, and interpersonal trust (Fig. 2).

In a group, we agree with the literature outside of health professions education that trust occurs at multiple levels encompassing both individual level and group level processes simultaneously. For example, in a competency committee, members must be able to reconcile differences of opinion and work with colleagues on the committee to make decisions that are in the best interests of not only their trainees, but ultimately the patients for whom their trainees provide care. Committee members must also be able to trust one another to pull their own weight on the committee, respect other individuals’ opinions, provide constructive input, and make fair judgments.

Higher levels of interpersonal trust will likely lead to a higher degree of trust at the group level. Higher degrees of trust at both levels have been associated with attitudinal outcomes, such as increased cooperation and satisfaction [22, 33, 40], and performance outcomes, such as information sharing and quality of group decisions [22, 42, 43, 55]. Additionally, developing a more thorough understanding of trust in the context of competency committees may help these committees implement more meaningful and effective processes to make collective decisions regarding trainee competence.

Based upon a synthesis of the information uncovered in this scoping review, we propose a model (Fig. 3) to better understand trust at the group level and the various factors that may influence the trust of group decisions. Our model uses situated cognition to incorporate trust at both levels along with other group level factors, environmental factors, and most importantly, the interactions between all of these elements. We also include practical implications for group decision-making, such as competency committees, in Table 3.

While this model has been empirically tested in limited settings [57], future research should further explore our model in group settings to determine the applicability of our proposed definition and factors that we contend influence group trust. We also suggest additional research looking at trust in group decisions from an external stakeholder perspective (e.g. a person who is not a competency committee member) and what, if any differences may exist between this and group trust. For competency committee decisions, this might involve examining how program directors and individual faculty members who are not on a competency committee define trust in this context. Observing additional competency committee meetings with our proposed group trust framework may provide additional insight about how group trust operates in health professions education settings.

Our research is limited by the fact that all of our data came from fields outside of health professions education, and trust in other occupational settings may not be the same as trust in health professions education settings. Similarly, some of the reviewed papers focused on undergraduate and graduate students, and characteristics of these learners may not be the same as trainees in health professions education. However, we attempted to tie together universal characteristics of trust across industries and populations studied to develop a model that may be applicable to health professions education groups and warrants further investigation. Indeed, we also provide specific examples of how the model applies directly to competency committees and the collective decisions they make (Table 3).

## Conclusions

This scoping review should allow educators and leaders to better appreciate what factors may contribute to trust in group settings (e.g. competency committees). As programs establish and refine policies, procedures, and membership of these committees, our proposed definition and model may help to improve the translation of evaluation data and individual opinions into competency decisions. Educators who understand these factors may also help create a collective environment of trust not only on a competency committee, but potentially within their organization as well. Trainees, patients, and other stakeholders trust competency committees to make carefully weighted decisions, and it is important to determine how these committees can demonstrate that this trust is not misplaced.

### Additional files

Additional file 1: Headings of the Extraction Tool. (DOCX 18 kb)

Additional file 2: Included Publications. (DOCX 28 kb)

Additional file 3: ERIC Search Publication Review. (DOCX 22 kb)

Additional file 4: PsycINFO Search Publication Review. (DOCX 117 kb)

Additional file 5: PUBMED Search Publication Review. (DOCX 43 kb)

Additional file 6: SCOPUS Search Publication Review. (DOCX 87 kb)

## Data Availability

All data generated and analyzed during this study are included in this published article and its supplementary information files. Complete data from the extraction tool are available from the corresponding author on reasonable request.

## Appendix

### Search Strategy

**ERIC Search strategy:** title:(trust OR trustworthy OR trustworthiness) AND title:(group OR groups OR team OR terms OR committee OR committee OR july OR judges) AND (decision OR decisions OR success OR success OR outcome OR outcomes).

**PsychINFO Search strategy:** ((trust or trustworth*) and (group or groups or team or teams or committee or committees or jury or juries)).ti. and (decision or decisions or success or successes or outcome or outcomes).af.**Limit:** (english language and (“0100 journal” or “0110 peer-reviewed journal” or “0120 non-peer-reviewed journal” or “0130 peer-reviewed status unknown”)).

**PUBMED Search strategy:** (“Trust”[Majr] OR trust[ti] OR trustworth*[ti]) AND (“Group Processes”[Majr] OR group[ti] OR groups[ti] OR team[ti] OR teams[ti] OR committee[ti] OR committees[ti] OR jury[ti] OR juries[ti]) AND (“Decision Making”[Mesh] OR decision*[tiab] OR success[tiab] OR outcome*[tiab]) AND English[lang].

**SCOPUS Search strategy:** (TITLE(trust OR trustworth*) AND TITLE(group OR groups OR team OR teams OR committee OR committees OR jury OR juries) AND TITLE-ABS-KEY(decision OR decisions OR success OR successes OR outcome OR outcomes)):

**Limit**: “English (language),” “article” (including “article in press”) and “review” (excludes “conference paper,” “book chapter,” “note,” “letter,” “short survey”):

## Abbreviations

EPA: Entrustable Professional Activity
GME: Graduate Medical Education
MBA: Master of Business Administration
