# Supplementary material for: Trust in Group Decisions: a scoping review
Source: BMC Med Educ. 2019 Aug 14;19:309. doi: 10.1186/s12909-019-1726-4 (PMC6693175; doi:10.1186/s12909-019-1726-4)
Supplement: Supplementary file 1 — Headings of the Extraction Tool. (DOCX 18 kb) [file 12909_2019_1726_MOESM1_ESM.docx]

**Additional file 1: Headings of the Extraction Tool**

| **General Article Characteristics** | | | | | |
| --- | --- | --- | --- | --- | --- |
| Reviewer | Year | Author(s) | Who are the authors? (medical; organizational psychologists; business; social scientists; other (please list)) | Title of article | Title of journal |
| Citation information (issues, page number(s)) | Citation type (commentary/editorial; original research paper - peer reviewed; original research paper - not peer reviewed; review paper; other (please list)) | Type of review (narrative; scoping; structured; systematic; systematic with meta-analysis; not stated but I infer the type of review was (please indicate); Other (please indicate)) | Geographic location of the study. If the location of the study (i.e. study population) is not possible or not relevant, select the location of the majority of the authors (Asia; Europe; North America; Oceania; South America; More than one location (please list)) | What type of individuals and/or groups are being studied/cited? (e.g. medical professionals; business professionals; educators; students; jurors, etc). If the individual type is different than the group type, please list both | If the article relates to medical individuals and/or groups, does it mention any specific areas or specialties? If so, please list |
| Is the paper presenting data from an original research study? | Please summarize the main purpose of the study. Direct quotations are preferred over using your own words. If you are quoting, please use quotation marks and indicate the page from which you are quoting | Approach to Data Collection | | | |
|  |  | Qualitative (Please summarize what kind of data was collected e.g. field notes of observations, interviews, focus groups, etc.) | Quantitative (Please summarize what kind of data was collected) | Mixed Methods (Please summarize what kind of data was collected in each study arm) | Other (Please describe) |
|  |  |  |  |  |  |
| **Definitions of Trust** | | | | | |
| Is an explicit definition of trust included? An example could be: "For the purpose of this study, we define trust as…" (please copy and paste the definition(s) of trust present in the paper using quotation marks and indicating page numbers for source quotes as appropriate) | Does the paper provide an explicit or implicit definition of individual trust? An example could be: "For the purpose of this study, we define individual trust as…" (please copy and paste the definition(s) of trust present in the paper using quotation marks and indicating page numbers for source quotes as appropriate) | Does the paper specify a group or team (which consists of more than two individuals) as a unit or level of analysis? If so, please provide quotes (and page numbers) regarding how the paper defines a group or team. If the paper refers to a collection of individuals other than a group or team, please indicate this as well | Does the paper provide information about a group or team making decisions about an individual? If so, please provide quotes (and page numbers) regarding how the paper discusses this | Does the article provide an explicit or implicit definition of group trust? An example could be: "For the purpose of this study, we define group trust as…" (please copy and paste definition(s) of the above mentioned word(s) present in this paper using quotation marks and indicating page numbers for source quotes as appropriate). If the paper refers to group trust as something different (e.g. collective trust, trust at the team/group level, trustworthiness of group decisions, etc), please provide this term as well |  |
|  |  |  |  |  |  |
| Are any theories or models mentioned in the article? If so, please list and provide specific references cited in the paper for each theory listed | | | | | |
|  |  |  |  |  |  |
| Does the article list individual factors that contribute to trust at the individual level? If so, please provide the term(s) under the category specified by the article | | | | | |
| Trustor characteristics (e.g. propensity to trust) | Trustee characteristics (e.g. ability/ competence, benevolence, integrity) | Interpersonal relationships (e.g. types and duration of relationships, community or geographic similarities) | Other (please also provide a category if this is listed in the article) | Comments |  |
|  |  |  |  |  |  |
| Does the article list group factors that contribute to trust at the group level? If so, please provide the term(s) under the category specified by the article | | | | | |
| Composition (e.g. demographics, diversity, degree of individualism) | Relationship structure (i.e. strength of ties in the group) | Leadership (e.g. leader characteristics, type of leader-member exchanges) | Climate (e.g. degree of psychological comfort within the group environment) | Task interdependence (i.e. the degree to which group members rely on one another and must interact in order for the group to accomplish its goals) | Other (please also provide a category if this is listed in the article) |
| Comments |  |  |  |  |  |
|  |  |  |  |  |  |
| Does the article list any team interaction or group process factors that contribute to trust at the group level (examples may include procedural fairness, transparency, inclusion, group/shared mental model, information sharing/distribution of knowledge, time pressures, groupthink, task/cognitive vs relational/affective conflict, cognitive vs affective trust, communication, etc) | | | | | Comments |
|  |  |  |  |  |  |
| Does the article list contextual/situational factors that contribute to trust at the group level? If so, please provide the term(s) under the category specified by the article | | | | | Comments |
|  |  |  |  |  |  |
| Does the article list individual level outcomes that result from trust at the group level? If so, please provide the term(s) under the category specified by the article | | | | | |
| Attitudes (e.g. job satisfaction/ stress, loyalty/ commitment to the organization, satisfaction with the group) | Performance (e.g. open communication, knowledge sharing, proactive idea implementation/ problem solving, cooperation) | Other (please also provide a category if this is listed in the article) | Comments |  |  |
|  |  |  |  |  |  |
| Does the article list group level outcomes that result from group at the team level? If so, please provide the term(s) under the category specified by the article | | | | | |
| Attitudes (e.g. group-level satisfaction, commitment, cohesiveness) | Performance (e.g. innovation, knowledge sharing, group learning/ information processing and organizational citizenship behaviors) | Other (please also provide a category if this is listed in the article) | Comments |  |  |
|  |  |  |  |  |  |
| Please summarize the main findings of the paper. Direct quotations are preferred over using your own words. If you are quoting, please use quotation marks and indicate the from which page you are quoting | | | | | |
|  |  |  |  |  |  |
| Does the article provide any practical implications of defining trust at the group level of analysis? If so, please describe | | | | | |
|  |  |  |  |  |  |
| Does the article provide implications for future research? If so, please list | | | | | |
|  |  |  |  |  |  |
| General comments/notes (anything you think wasn't captured) | | | | | |
