# Supplementary material for: Trust in Group Decisions: a scoping review
Source: BMC Med Educ. 2019 Aug 14;19:309. doi: 10.1186/s12909-019-1726-4 (PMC6693175; doi:10.1186/s12909-019-1726-4)
Supplement: Supplementary file 2 — Included Publications. (DOCX 28 kb) [file 12909_2019_1726_MOESM2_ESM.docx]

**Additional file 2: Included Publications**

| **Citation** | **Type of Article** | **Geographic Origin of the Study** | **Study Population** | **Approach to Data Collection** |
| --- | --- | --- | --- | --- |
| Bianchi EC, Brockner J, van den Bos K et al. Trust in decision-making authorities dictates the form of the interactive relationship between outcome fairness and procedural fairness. Pers Soc Psychol B. 2015;41:19–34. | Original research | United States | New employees in a large global shipping company and community sample | Quantitative - survey |
| Brown G, Crossley C, Robinson S. Psychological ownership, territorial behavior, and being perceived as a team contributor: the critical role of trust in the work environment. Pers Psychol. 2014;67:463-85. | Original research | Singapore | Employees in a variety of occupations | Quantitative – survey |
| Carmeli A, Tishler A, Edmondson A. CEO relational leadership and strategic decision quality in top management teams: the role of team trust and learning from failure. Strateg Organ. 2011;10:31-54. | Original research | Israel | CEOs and employees on top management teams (TMTs) from firms in a variety of industries | Mixed Methods – survey and in-depth qualitative analysis of two TMTs |
| Costa A, Roe R, Taillieu T. Trust within teams: the relation with performance effectiveness. Eur J Work Organ Psy. 2001;10:225-44. | Original research | Netherlands | Teams from three social care institutions | Quantitative – survey |
| Costa A. Work team trust and effectiveness. Pers Rev. 2003;32:605-22. | Original research | Netherlands | Employees of three social care institutions | Quantitative – survey |
| Costa A, Anderson N. Measuring trust in teams: development and validation of a multifaceted measure of formative and reflective indicators of team trust. Eur J Work Organ Psy. 2011;20:119-54. | Original research | Netherlands | Employees of a hospital and three social care organizations | Quantitative – survey |
| Costa A, Fulmer C, Anderson N. Trust in work teams: an integrative review, multilevel model, and future directions. J Organ Behav. 2017;1-16. | Review paper | United Kingdom | N/A | Review paper |
| Curseu P, Schruijer S. Does conflict shatter trust or does trust obliterate conflict? Revisiting the relationships between team diversity, conflict, and trust. Group Dyn-Theor Res. 2010;14:66-79. | Original research | Netherlands | Undergraduate students | Quantitative – survey |
| Dayan M, Benedetto CA. The impact of structural and contextual factors on trust formation in product development teams. Ind Market Manag. 2010;39:691-703. | Original research | United Arab Emirates | New product development teams | Quantitative – survey |
| De Jong B, Dirks K. Beyond shared perceptions of trust and monitoring in teams: implications of asymmetry and dissensus. J Appl Psychol. 2012;97:391-406. | Original research | Netherlands | Board members of various Dutch student associations and employees of a healthcare organization | Quantitative – survey |
| De Jong B, Dirks K, Gillespie N. Trust and team performance: a meta-analysis of main effects, moderators, and covariates. J Appl Psychol. 2016;101:1134-50. | Systematic review and meta-analysis | Netherlands | N/A | Systematic review and meta-analysis |
| DeOrtentiis P, Summers, J, Ammeter A, Douglas C, Ferris G. Cohesion and satisfaction as mediators of the team trust – team effectiveness relationship: an interdependence theory perspective. Career Dev Int. 2013;18:521-43. | Original research | United States | MBA graduate students | Quantitative - survey |
| Ennen NL, Stark E, Lassiter A. The importance of trust for satisfaction, motivation, and academic performance in student learning groups. Soc Psychol Educ. 2015;18:615–33. | Original research | United States | Undergraduate students | Quantitative - survey |
| Ferguson A, Peterson R. Sinking slowly: diversity in propensity to trust predicts downward trust spirals in small groups. J Appl Psychol. 2015;100(4):1012-24. | Original research | United Kingdom | Graduate MBA students | Quantitative – survey |
| Hakanen M, Hakkinen M, Soudunsaari A. Trust in building high-performing teams – conceptual approach. Electron J Bus Ethics Organ Stud. 2015;20:43-53. | Review paper | Finland | N/A | Review paper |
| Han G, Harms P. Team identification, trust, and conflict: A mediation model. Int J Confl Manage. 2010;21:20-43. | Original research | United States | Employees of a Fortune 500 company | Quantitative – survey |
| Korsgaard M, Schweiger D, Sapienza H. Building commitment, attachment, and trust in strategic decision-making teams: the role of procedural justice. Acad Manage J. 1995;38:60-84. | Original research | United States | Management teams of a Fortune 500 company | Quantitative – survey |
| Kweekel L, Gerrits T, Rijnders M, Brown P. The role of trust in CenteringPregnancy (CP): building interpersonal trust relationships in group-based prenatal care in the Netherlands. Birth. 2017;44:41-7. | Original research | Netherlands | Former participants in Centering- Pregnancy (CP) who received prenatal care at one of four midwifery practices | Qualitative – semi-structured interviews and structured observations |
| LaMacchia ST, Louis WR, Hornsey MJ, Leonardelli GJ. In small we trust: lay theories about small and large groups. Pers Soc Psychol B. 2016;42:1321–34. | Original research | Australia | Undergraduate students and community samples | Mixed Methods – quantitative and qualitative surveys |
| Langfred C. The downside of self-management: a longitudinal study of the effects of conflict on trust, autonomy, and task interdependence in self-managing teams. Acad Manage J. 2007;50:885-900. | Original research | United States | MBA graduate students | Quantitative – survey |
| Lowry P, Zhang D, Zhou L, Fu X. Effects of culture, social presence, and group composition on trust in technology-supported decision-making groups. Inform Syst J. 2010;20:297-315. | Original research | United States | Undergraduate students | Quantitative – scenario |
| Lvina E, Maher L, Harris J. Political skill, trust, and efficacy in Teams. J Leadersh Org Stud. 2017;24:95-105 | Original research | Canada | Undergraduate students | Quantitative – survey |
| Mayfield C, Tombaugh J, Lee M. Psychological collectivism and team effectiveness: moderating effects of trust and psychological safety. J Organ Cult Comm Conflict. 2016;20:78-94. | Original research | United States | Graduate and upper-division undergraduate students enrolled in business and education courses | Quantitative – survey |
| Moreland R, Levine J. Socialization and trust in work groups. Group Process Intergr. 2002;5:185-201. | Review paper | United States | N/A | Review paper |
| Naquin C, Kurtzberg T. Team Negotiation and perceptions of trustworthiness: the whole versus the sum of the parts. Group Dyn-Theor Res. 2009;13:133-50. | Original research | United States | Graduate-level business students | Quantitative – survey |
| Olvera J, Llorens S, Acosta H, Salanova M. Transformational leadership and horizontal trust as antecedents of team performance in the healthcare context. Ann Psychol. 2017;33:365-75. | Original research | Spain | Employees from work teams and supervisors of these teams from four healthcare centers | Quantitative – survey |
| Parayitam S, Dooley R. The relationship between conflict and decision outcomes: moderating effects of cognitive- and affect-based trust in strategic decision-making teams. Int J Confl Manage. 2007;18:42-73. | Original research | United States | Hospital employees on strategic decision making teams | Quantitative – survey |
| Peterson R, Behfar K. The dynamic relationship between performance feedback, trust, and conflict in groups: A longitudinal study. Organ Behav Hum Dec. 2003; 92:102-112. | Original research | United States | Graduate MBA students | Quantitative – survey |
| Poltis J. The connection between trust and knowledge management: what are its implications for team performance. J Knowl Manag. 2003;7:55-66. | Original research | Australia | Members of self-managing teams from a large high-technology, aerospace, manufacturing organization | Quantitative – survey |
| Rau D. The influence of relationship conflict and trust on the transactive memory: performance relation in top management teams. Small Gr Res. 2005;36:746-71. | Original research | United States | Members of commercial bank top management teams | Quantitative – survey |
| Robertson M. Trust: the power that binds in team supervision of doctoral students. High Educ Res Dev. 2017;36:1463-75. | Original research | Australia | Late-stage doctoral students and doctoral supervisors | Qualitative – in-depth, semi-structured interviews |
| Selmer J, Jonasson C, Lauring J. Group conflict and faculty engagement: is there a moderating effect of group trust? J High Educ Pol Manage. 2013;35:95-109. | Original research | Denmark | Academic staff members in three large universities | Quantitative – survey |
| Seppala T, Lipponen J, Pirttila-Backman A. Leader fairness and employees’ trust in coworkers: the moderating role of leader group prototypicality. Group Dyn-Theor Res. 2012;16:35-49. | Original research | Finland | Employees in work groups in two different organizations | Quantitative – survey |
| Simons TL, Peterson RS. Task conflict and relationship conflict in top management teams: the pivotal role of intragroup trust. J Appl Psychol. 2000;85:102-11. | Original research | United States | CEOs and management teams of hotel companies | Mixed Methods – surveys and interviews |
| Spector M, Jones G. Trust in the workplace: factors affecting trust formation between team members. J Soc Psychol. 2004; 144: 311-21. | Original research | United States | Professional employees working regularly in an office environment of 19 major corporations | Quantitative – survey |
| Tanghe J, Wisse B, van der Flier H. The role of group member affect in the relationship between trust and cooperation. Brit J Manage. 2010;21:359-74. | Original research | Netherlands | Undergraduate students | Quantitative – survey |
| Terwel BW, Harinck F, Ellemers N, Daamen D. Voice in political decision-making: the effect of group voice on perceived trustworthiness of decision makers and subsequent acceptance of decisions. J Exp Psychol-Appl. 2010;16:173-86. | Original research | Netherlands | Undergraduate students | Quantitative – scenario |
| van der Zee K, Vos M, Luijters K. Social identity patterns and trust in demographically diverse work teams. Soc Sc Inform. 2009;48:175-98. | Review paper | Netherlands | N/A | Review paper |
| Walumba F, Luthans F, Avey J, Oke A. Authentically leading groups: the mediating role of collective psychological capital and trust. J Organ Behav. 2011;32:4-24. | Original research | United States | Employees and their immediate supervisors of a large bank | Quantitative – survey |
| Webber S. Leadership and trust facilitating cross-functional team success. J Manage Dev. 2002;21:201-14. | Review paper | Canada | N/A | Review paper |
| Wildman J, Shuffler M, Lazzara E, Fiore S, Burke C, Salas E, Garven S. Trust development in swift starting action teams: a multilevel framework. Group Organ Manage. 2012;37:137–70. | Review paper | United States | N/A | Review paper |
| Yang I. What makes an effective team? The role of trust (dis)confirmation in team development. Eur Manage J. 2014;32:858-69. | Review paper | France | N/A | Review paper |
