# Supplementary material for: Trust in Group Decisions: a scoping review
Source: BMC Med Educ. 2019 Aug 14;19:309. doi: 10.1186/s12909-019-1726-4 (PMC6693175; doi:10.1186/s12909-019-1726-4)
Supplement: Supplementary file 3 — ERIC Search Publication Review. (DOCX 22 kb) [file 12909_2019_1726_MOESM3_ESM.docx]

**Additional file 3: ERIC, 13 April 2018 (titles), 16 April 2018 (abstracts), 12 June 2018 (re-review abstracts)**

**Search strategy:** title:(trust OR trustworthy OR trustworthiness) AND title:(group OR groups OR team OR terms OR committee OR committee OR july OR judges) AND (decision OR decisions OR success OR success OR outcome OR outcomes): **19 results, 11 “exclude,” 8 “include in abstract review”**

**16 April 2018: 8 “include in abstract review, 1 “exclude,” 7 “include in article review”**

**12 June 2018 (re-review): 2 additional “exclude,” 5 “include in article review” (1 duplicate)**

**9 Oct 2018 review: 1 additional “exclude,” 4 “include in article review)**

**ABSTRACT REVIEW (8 articles)**

**INCLUDE IN ARTICLE REVIEW (This abstract was reviewed on the PUBMED sheet)** Voice in Political Decision-Making: The Effect of Group Voice on Perceived Trustworthiness of Decision Makers and Subsequent Acceptance of Decisions
Terwel, Bart W.; Harinck, Fieke; Ellemers, Naomi; Daamen, Dancker D. L. – Journal of Experimental Psychology: Applied, 2010 (Peer reviewed)
Descriptors: Decision Making, Politics, Credibility, Trust (Psychology)

**INCLUDE IN ARTICLE REVIEW (coded)** The Importance of Trust for Satisfaction, Motivation, and Academic Performance in Student Learning Groups
Ennen, Nicole L.; Stark, Emily; Lassiter, Andrea – Social Psychology of Education: An International Journal, 2015 (Peer reviewed)
Descriptors: Trust (Psychology), Academic Achievement, Cooperative Learning, Psychology

Educators are continuing to investigate ways to improve student learning through collaboration. This study examined one avenue of increasing student group effectiveness: trust. A model of trust in student workgroups was proposed, where trust mediates the relationships between perceived similarity and individual outcomes (grades and satisfaction). Participants in this study included 252 psychology students at a Midwestern university who participated in semester-long group work in the classroom. The findings indicated that students who perceived themselves as similar to their group members were more likely to trust the group. For the outcome measures, trust was positively related to grades; students who had higher levels of trust towards their group members received higher grades than those with lower levels of trust. In addition, trust was strongly and positively related to satisfaction with one's group and motivation to work in groups in the future. Additionally, trust emerged as a mediator between perceived similarity and satisfaction, but trust did not mediate links between perceived similarity and academic performance. Finally, an exploratory analysis comparing group environments indicated that face-to-face groups may have higher levels of trust than virtual groups. This study adds to current literature by examining an antecedent of trust (i.e., perceived similarity), by linking trust to a performance-based outcome in student groups (i.e., grades), and by supporting previous lab-based findings linking trust to satisfaction and motivation using actual student learning groups.

**INCLUDE IN ARTICLE REVIEW (coded)** Trust: The Power That Binds in Team Supervision of Doctoral Students
Robertson, Margaret J. – Higher Education Research and Development, 2017 (Peer reviewed)
Descriptors: Teamwork, Doctoral Programs, Supervision, Qualitative Research

Team supervision of doctoral students adds new dimensions and complexities to relationships within the teams that impact functionality of the team. Trust emerged as a significant theme in recent qualitative research into the quality of team supervision of doctoral students. Trust was cited as a key component in successful team collaborations, and the missing component in dysfunctional teams. Definitions refer to trust as the belief of truthfulness, reliability or faith in another person's abilities. My hypothesis is that trust operates as a form of power in team supervision. It is a form of power that enables voice, resilience and creativity in teams. This article concludes that placing trust in others in supervisory teams is a deliberate decision by candidates and supervisors. It is a decision to engage in a team context despite known risks and may be understood as a gamble on the reliability of others in the expectation of reciprocity. By conceptualising trust as a form of power, strategies that engender and maintain trust may be utilised more intentionally.

**INCLUDE IN ARTICLE REVIEW (coded)** Group Conflict and Faculty Engagement: Is There a Moderating Effect of Group Trust?
Selmer, Jan; Jonasson, Charlotte; Lauring, Jakob – Journal of Higher Education Policy and Management, 2013 (Peer reviewed)
Descriptors: Trust (Psychology), Conflict, College Faculty, Collegiality

In educational settings, substantial scholarly interest has focused on student engagement as an antecedent for educational development and positive school outcomes. Very limited research, however, has focused on the engagement of academic staff members. This may be a crucial oversight because engagement has been argued to lead to more satisfied, more productive and healthier staff. In this study, based on a sample consisting of 489 members of multicultural university departments, we set out to investigate the relationship between trust, conflict and academic staff engagement. More specifically we assessed the effect of group trust, group relational conflict and group task conflict on indicators of behavioural, cognitive and emotional engagement. Our findings show a strong positive association between group trust and all academic staff engagement variables as well as a strong negative association between group relational conflict and all staff engagement variables. Task conflict was negatively associated with indicators of staff cognitive engagement. However, surprisingly, group trust did not have any moderating effect. Implications for educational organisation managers and policy makers are discussed in detail. (Contains 3 tables.)

**INCLUDE IN ARTICLE REVIEW (waiting to code - Virtual Teams - 7)** A Cross-Disciplinary Literature Review: Examining Trust on Virtual Teams
Berry, Gregory R. – Performance Improvement Quarterly, 2011
Descriptors: Electronic Learning, Business Communication, Trust (Psychology), Virtual Classrooms

Effective and efficient teams communicate, collaborate, and perform, even if these teams are not co-located. Although much is known about enabling effectiveness on face-to-face teams, considerably less is known about similarly enabling effectiveness on virtual teams. Yet the use of virtual teams is common and will likely become more commonplace as organizations continue to update and use increasingly sophisticated technology. Cohort and individual trust between team members is a significant component on most effective teams, virtual or face-to-face. This paper examines research on virtual teams and trust across disciplines, including management, e-learning, business communication, decision making, human resource management, psychology, and IT, to identify major characteristics that increase our knowledge of how to establish and maintain trust in the virtual environment and on virtual teams. (Contains 2 tables.)

**EXCLUDE (This paper seems more focused on leadership training and school administrators. It also seems very institution-specific and not applicable to CCCs - 2)** Instructional Strategies Designed to Develop Trust and Team Building Skills in School Leaders
Freeman, Virgil – International Journal of Educational Leadership Preparation, 2006 (Peer reviewed)
Descriptors: Leadership Training, Leadership Qualities, Team Training, Educational Strategies

This paper will provide readers with insight into "Team Building and Decision-Making" curriculum methods and strategies utilized in leadership for our administrators. The content is based on "The Five Dysfunctions of a Team: A Leadership Fable" by Lencioni (2002). The writer is working on developing a unique cascade of activities designed to reduce team dysfunctions through trust and skill building.

**EXCLUDE (Trust and the context it is used in this setting is not really similar to a CCC, this is also more of an informational paper that only looks very briefly at trust. 12 June 2018 - 2)** Give Teams a Running Start: Take Steps to Build Shared Vision, Trust, and Collaboration Skills
Kise, Jane A. G. – Journal of Staff Development, 2012 (Peer reviewed)
Descriptors: Cooperation, Faculty Development, Communities of Practice, Academic Achievement

Consider for a moment how launching a professional learning community is similar to starting a race. Athletes know the danger of false starts--moving before the starting signal. Until recently, a false start meant that all racers returned to the blocks to begin again, their adrenalin gone, their concentration broken. Because these effects could influence race results, the rules changed. Races continue, and competitors who false start learn only at the end that they've been disqualified. When professional learning communities have a false start, no one blows a whistle, but members' initial energy for collaboration can dissipate when they run into all-too-common barriers such as lack of clarity around vision and purpose, trust issues, or insufficient time available for the scope of the undertaking, to name a few. These barriers to effective collaboration are real, yet school leaders who aren't aware of the multistage nature of professional learning community initiatives often launch them before working to remove these impediments. Targeting three key barriers--lack of shared vision, trust, and collaboration skills--can remove hurdles while at the same time beginning, or re-energizing, the work of professional learning communities.

**EXCLUDE (The concept of a self-managed school is not very similar to a CCC, especially this article, which I reviewed the full text and the concept of trust does not apply to our setting. 12 June 2018 - 3)** The Power of Trust: Teams and Collective Action in Self-Managed Schools.
Henkin, Alan B.; Dee, Jay R. – Journal of School Leadership, 2001 (Peer reviewed)
Descriptors: Case Studies, Cooperation, Elementary Education, Parent Participation

Trust is a significant factor in collective action. A composite case illustrates the value of trust as a critical element of effective teamwork in a self-managed school context. Trust depends on individuals' predilections, intensive communication and interaction patterns, team composition, guiding principles, and teammates' emotional bonding. (Contains 57 references.) (MLH)

**EXCLUDE (these articles have already been excluded based upon the title review):**

**EXCLUDE (population studied and self-selection for a team assignment not really applicable or transferrable to CCCs - 4)** Social Cues of (Un)Trustworthy Team Members
Neu, Wayne A. – Journal of Marketing Education, 2015 (Peer reviewed)
This study investigates the way in which and the extent to which students engage in social categorization during the process of self-selecting team members for a team assignment. The discovery-oriented method of grounded theory was used. Data were gathered from a sample of 38 undergraduate marketing and management students using the Zaltman…
Descriptors: Cues, Trust (Psychology), Undergraduate Students, Business Administration Education

**EXCLUDE (dissertation - 6)** Knowledge Sharing in Virtual Teams: The Impact on Trust, Collaboration, and Team Effectiveness
Alsharo, Mohammad K. – ProQuest LLC, 2013
Virtual teams are utilized by organizations to gather experts to collaborate online in order to accomplish organizational tasks. However, the characteristics of these teams create challenges to effective collaboration and effective team outcome. Collaboration is an essential component of teamwork, the notion of forming teams in organizations is…
Descriptors: Trust (Psychology), Teamwork, Computer Mediated Communication, Cooperation

**EXCLUDE (dissertation - 6)** The Development of Team Trust over Time and Its Effect on Performance When Using Michaelsen's Team-Based Learning
Preast, Vanessa A. – ProQuest LLC, 2012
Proponents of Michaelsen's Team-Based Learning (TBL) have claimed this teaching method quickly produces highly effective teams which are characterized by high trust among team members. Presumably, the high trust boosts performance because members feel less inhibited during discussions involving sharing personal views and challenging…
Descriptors: Teamwork, Cooperative Learning, Teaching Methods, Performance

**EXCLUDE (dissertation - 6)** Understanding the Adaptive Use of Virtual World Technology Capabilities and Trust in Virtual Teams
Owens, Dawn – ProQuest LLC, 2012
In an environment of global competition and constant technological change, the use of virtual teams has become commonplace for many organizations. Virtual team members are geographically and temporally dispersed, experience cultural diversity, and lack shared social context and face-to-face encounters considered as irreplaceable for building and…
Descriptors: Teamwork, Computer Mediated Communication, Educational Technology, Interaction

**EXCLUDE (dissertation - 6)** The Changes in Relational Trust during the First Year of a Distributed Leadership Implementation: A Descriptive Study on the Changes of Trust among Distributed Leadership Teams
Rios, Francisco Javier Larrain – ProQuest LLC, 2017
This study examined the effects of a school improvement project involving Distributed leadership (DL), a perspective for studying or developing organization leadership through the interaction of organizational members and activities. This research was part of a larger DL Project taking place in York City School District, PA, which sought to…
Descriptors: Trust (Psychology), Participative Decision Making, Change, Leadership

**EXCLUDE (speech/meeting paper - 6)** Trust in Academic Leaders and Committee Operation.
Dufty, N. F. – Journal of Tertiary Educational Administration, 1980
One of the problems in the governance of academic institutions is described as the presence of a complex network of committees with a set of powerful individuals. Powerful individuals emerge because of personal qualities and because some decision-making modes must be established for decisions that cannot wait for committees. (Author/MLW)
Descriptors: Administrative Organization, College Administration, College Faculty, Committees

**EXCLUDE (population studied, subjects - college academic leadership, and age of the article not really applicable or transferrable to CCCs - 3)** Trust in Academic Leaders and Committee Operation.
Duffy, N. F. – Journal of Tertiary Educational Administration, 1980
A survey in an Australian technical institute focused on the relationship between faculty's trust in academic leadership on campus and the operation of committees, in the context of institutional governance and administration. Attitudes toward administrators and committees, satisfaction with decisions, information sources, and perceived channels…
Descriptors: Administrators, College Administration, Committees, Credibility

**EXCLUDE (short-term group therapy not really applicable to transferrable to CCCs - 5)** Dimensions of Interpersonal Trust and Group Variables in Short-Term Counseling.
Walker, Betty A.; Robinson, Rick – Educational Research Quarterly, 1979 (Peer reviewed)
Two dimensions of the Rotter Interpersonal Trust Scale (ITS) were used to investigate the relationship between interpersonal trust and three variables in short-term group therapy: group cohesiveness, group status, and immediate outcome. Groups of a heterogeneous mix--according to ITS scores--had significantly higher cohesiveness scores.…
Descriptors: Counseling Effectiveness, Credibility, Group Counseling, Group Dynamics

**EXCLUDE (cash games not really applicable to transferrable to CCCs - 6)** EASY MONEY: An Exploration of Trust in Teams.
Thiagarajan, Sivasailam – Simulation & Gaming, 1997 (Peer reviewed)
Cash games are simulation activities which feature real cash prizes and explore interpersonal skills and concepts. EASY MONEY is one such game, featuring an investment activity which focuses on trust among team members in ambiguous decision-making situations. Directions for administering the game and suggestions for applying a standard six-phase…
Descriptors: Cooperative Learning, Decision Making, Group Behavior, Group Unity

**EXCLUDE (opinion paper - 6)** Management Team Builds Trust between Board, Superintendent.
Brooks, Maryanne – Thrust for Educational Leadership, 1978
A good management team can build trust and confidence between the board and the superintendent, as well as the site administrators and staff, because everybody has a chance to know what is going on, why, and help contribute to finding answers, rather than being on the sidelines. (Author)
Descriptors: Administrative Organization, Board Administrator Relationship, Board of Education Policy, Decision Making

**EXCLUDE (IGE = individually guided elementary education, not really applicable or transferrable to CCCs - 3)** Objectives, Competencies, and Trust--They're All Essential For Effective Group Functioning
Bechtol, William M.; And Others – Journal of Teacher Education, 1976 (Peer reviewed)
Teachers in IGE should emphasize and explain the goals, instructional objectives, and philosophy of the unit team. (MM)
Descriptors: Decision Making, Elementary School Teachers, Individual Differences, Interpersonal Relationship
