# Supplementary material for: Trust in Group Decisions: a scoping review
Source: BMC Med Educ. 2019 Aug 14;19:309. doi: 10.1186/s12909-019-1726-4 (PMC6693175; doi:10.1186/s12909-019-1726-4)
Supplement: Supplementary file 4 — PsycINFO Search Publication Review. (DOCX 117 kb) [file 12909_2019_1726_MOESM4_ESM.docx]

**Additional file 4: PsycINFO, 8 April 2018**

**Search strategy:** ((trust or trustworth*) and (group or groups or team or teams or committee or committees or jury or juries)).ti. and (decision or decisions or success or successes or outcome or outcomes).af.: **238 Results**

**limit** 1 to (english language and ("0100 journal" or "0110 peer-reviewed journal" or "0120 non-peer-reviewed journal" or "0130 peer-reviewed status unknown")): **180 Results, 43 “exclude,” 137 “include in abstract review”**

**20 April 2018: 137 “include in abstract review,” 84 “exclude,” 53 “include in article review”**

**11 July 2018: 1 additional “exclude” after full text review**

**12 July 2018: 1 additional “exclude” after full text review**

**16 July 2018: 1 additional “exclude” after full text review**

**10 Aug 2018: 1 additional “exclude” after full text review**

**9 Oct 2018: 6 “waiting to code - Virtual Teams”**

**ABSTRACT REVIEW (137 articles)**

1. **EXCLUDE (Although interesting from my perspective, an eight-mission peachkeeping simulation seems a bit of a stretch in comparison to a longitudinal group making decisions about medical trainee competence - 4)**
Team perceived trustworthiness in a complex military peacekeeping simulation. [References].
Lee, Adrienne Y; Bond, Gary D; Russell, Deborah C; Tost, Jeremy; Gonzalez, Carlo; Scarbrough, Pamela S.
Military Psychology. Vol.22(3), 2010, pp. 237-261.
[Journal; Peer Reviewed Journal]
Year of Publication
2010
Publication Month/Season
Jul

AB Antecedents to trust (propensity to trust, perceived trustworthiness) and trust behaviors were examined in relation to team performance in a complex eight-mission military peacekeeping simulation. Teams were colocated or distributed and stayed in the same or transferred to a different context at task transfer. In Experiment 1, an ability and competence factor accounted for most of the variance in perceived trustworthiness and greater perceptions of teammates' abilities/competences predicted posttransfer performance. One's perception of how others perceived one's ability/competence increased over missions. In Experiment 2, propensity to trust did not predict performance; however, trust in others' and one's own ability/competence and trust behaviors predicted performance. At task and/or context transfer, teams produced more monitoring and less cooperating language in their communication.

2. **INCLUDE IN ARTICLE REVIEW (waiting to code - Virtual Teams - 7)**
The mediating effect of interpersonal trust on virtual team's collaboration. [References].
David, Kauffmann; Golan, Carmi.
International Journal of Knowledge Management. Vol.13(3), 2017, pp. 20-37.
[Journal; Peer Reviewed Journal]
Year of Publication
2017
Publication Month/Season
Jul-Sep

This article examines the relationship between task-communication and five collaborative processes by exploring the mediating effect of interpersonal trust in a virtual team's environment. A multiple mediation model was developed to examine this relationship where cognitive-based trust and affective-based trust are defined as mediation variables between task-communication and the five processes of collaboration. The main results of this study show a significant correlation with a large effect size between task communication, trust and collaboration. Also, interpersonal trust is playing an important role as a mediating element in the relationship between task communication and collaboration. This is where the emotional side of trust is no less important than the rational side, if not even more, in some collaborative processes.

3. **EXCLUDE (This article looks at semi-virtual collaboration groups, individual trust, and individual performance (as determined by a grade). This does some to be very applicable to CCCs. - 7)**
Investigating the individual trust and school performance in semi-virtual collaboration groups. [References].
Cheng, Xusen; Fu, Shixuan; Han, Yajing; Zarifis, Alex.
Information Technology & People. Vol.30(3), 2017, pp. 691-707.
[Journal; Peer Reviewed Journal]
Year of Publication
2017

Purpose: The purpose of this paper is to investigate the relationship between individual trust of students in computer supported semi-virtual collaboration groups and student's performance in school. Design/methodology/approach: Longitudinal questionnaires and interviews are conducted during the case study. By analyzing the data from the questionnaires and the grade earned by the students, the sample students are ranked with respect to the trust level and individual performance. Furthermore, the Wilcoxon signed-rank test is used to compare individual trust level and performance in the computer supported semi-virtual collaborative environment. Findings: The distribution of an individual's trust level is roughly consistent with the distribution of the individual's performance in the collaboration. Besides, the relationship between a student's trust level and the student's performance is positively correlated. Research limitations/implications: This study integrates the issues of trust, school performance, and collaboration in an educational context. Furthermore, the conclusions drawn from this paper extend the literature of multiple disciplines including education, management, and psychology. Practical implications: The conclusions could apply in the fields of education and management since the analysis revealed the relationship between an individual's trust level and their performance. Originality/value: This study contributes to the field of trust and collaboration research with a link to trust development and performance. The study also provides an insight into how to successfully improve the performance of student semi-virtual collaboration groups.

4. **INCLUDE IN ARTICLE REVIEW (This abstract was reviewed on the SCOPUS sheet)**
Virtual team effectiveness: The role of knowledge sharing and trust. [References].
Alsharo, Mohammad; Gregg, Dawn; Ramirez, Ronald.
Information & Management. Vol.54(4), 2017, pp. 479-490.
[Journal; Peer Reviewed Journal]
Year of Publication
2017
Publication Month/Season
Jun

5. **INCLUDE IN ARTICLE REVIEW (This abstract was reviewed on the ERIC document)**
Trust: The power that binds in team supervision of doctoral students. [References].
Robertson, Margaret J.
Higher Education Research & Development. Vol.36(7), 2017, pp. 1463-1475.
[Journal; Peer Reviewed Journal]
Year of Publication
2017
Publication Month/Season
Nov

6. **EXCLUDE (This article looks at intellectual property rights as antecedents of trust, which will not be applicable to a CCC - 4)**
The dynamics of intellectual property rights for trust, knowledge sharing and innovation in project teams. [References].
Olaisen, Johan; Revang, Oivind.
International Journal of Information Management. Vol.37(6), 2017, pp. 583-589.
[Journal; Peer Reviewed Journal]
Year of Publication
2017
Publication Month/Season
Dec

The research question is: How can intellectual property rights (IPRs) influence trust, attitudes, commitment, knowledge sharing, and innovation in inter-organisational project teams? The four strategically selected team cases include eight global knowledge-intensive industrial oil service companies in Norway. The methodology included 24 in-depth interviews done in 2016. The study finds that formal intellectual property rights are key to building up and keeping trust in the team and also for building up the right attitudes within the team. The IPRs increased the innovativeness in the team and incremental innovations. The IPRs fostered a unique knowledge sharing in these four teams enabling them to work towards innovative solutions and delivering in time. Formal IPR's foster informal trust and expertise sharing and by that also the inter- organizational cooperation. The confidence and knowledge sharing strengthen the possibility for future collaboration and innovations both on an individual level and on a corporate level. The theoretical implication of our findings is that IPRs increase the trust, commitment, and attitudes within the team providing knowledge sharing and innovativeness for improved solutions and results. IPRs are positive for collaboration, and they are complementary governance mechanisms. The practical implication is that IPRs must be defined and accepted before the corporations start up the inter-organizational teamwork. The contract typology should in the start up be sensitizing giving directions and security and in the end definitive.

7. **EXCLUDE (The concepts of social and commercial entrpreneurship, which seem to be the outcomes of this study, really aren’t applicable to CCCs - 2)**
Informal institutions and their comparative influences on social and commercial entrepreneurship: The role of in-group collectivism and interpersonal trust. [References].
Pathak, Saurav; Muralidharan, Etayankara.
Journal of Small Business Management. Vol.54(Suppl 1), 2016, pp. 168-188.
[Journal; Peer Reviewed Journal]
Year of Publication
2016
Publication Month/Season
Oct

We use insights from institutional theory to study how societal collectivism and societal trust facilitate or constrain the emergence of social entrepreneurship (SE) and commercial entrepreneurship (CE). Using 58,918 individual-level responses obtained from the Global Entrepreneurship Monitor (GEM) survey of 27 countries and supplementing with country-level data obtained from the World Values Survey (WVS) and the Global Leadership and Organizational Behavior Effectiveness (GLOBE) study, our results from multilevel analyses demonstrate that while societal collectivism decreases the likelihood of CE, it increases that of SE. Further, while societal trust influences both SE and CE positively, the strength of this positive influence is felt more strongly on SE than CE.

9. **INCLUDE IN ARTICLE REVIEW (This abstract was reviewed on the SCOPUS document)**
Trust in work teams: An integrative review, multilevel model, and future directions. [References].
Costa, Ana Cristina; Fulmer, C. Ashley; Anderson, Neil R.
Journal of Organizational Behavior. Vol.39(2), 2018, pp. 169-184.
[Journal; Peer Reviewed Journal]
Year of Publication
2018
Publication Month/Season
Feb

10. **EXCLUDE (Although humor can sometimes come up during a CCC meeting and is a novel idea to consider for this study, the population studied in this article (financial organizations) and outcomes (employee inclusion and organizational citizenship behavior) aren’t as applicable to CCCs. This is a touchy subject and recommend not using this to make inferences about use of humor in the setting of CCCs - 2)**
Humor in teams: Multilevel relationships between humor climate, inclusion, trust, and citizenship behaviors. [References].
Tremblay, Michel.
Journal of Business and Psychology. Vol.32(4), 2017, pp. 363-378.
[Journal; Peer Reviewed Journal]
Year of Publication
2017
Publication Month/Season
Aug

Purpose: This study examines the cross-level influence of positive and offensive leader humor climates on employee inclusion and citizenship behaviors, and the moderating effect of trust in such relationships. Design/Methodology/Approach: We collected data from a sample of 225 respondents nested within 23 teams from a Canadian financial organization. A multilevel confirmatory analysis was used to provide evidence that variables of this study are distinct and a HLM analysis to test the hypotheses. Findings: We find that employees' perception of inclusion is influenced much more by an offensive humor climate than by a positive one. The results also suggest that the perception of inclusion plays a significant intermediary role in the influence of humor climates on citizenship behavior. Finally, trust in leaders acts as an important contingent condition in the effectiveness of a humor climate. Implications: Use of humor does not always pay. Offensive humor by supervisor is a risky strategy that may undermine the beneficial effects of positive humor climate, increase employee exclusion and weaker individual performance. Originality/Value: Our study shows the utility of using micro- and macro-approaches, and more specifically, the relevance of adopting an integrative multilevel view of the effect of a humor environment in predicting individual inclusion and citizenship behaviors.

11. **INCLUDE IN ARTICLE REVIEW (EXCLUDE, This article was reviewed on the SCOPUS document, see discussion as to why it was excluded on that sheet)**
Trust toward a group of strangers as a function of stereotype-based social identification. [References].
Kong, Dejun Tony.
Personality and Individual Differences. Vol.120 2018, pp. 265-270.
[Journal; Peer Reviewed Journal]
Year of Publication
2018
Publication Month/Season
Jan

14. **EXCLUDE (This article looks at what seems to be a complicated interplay of intergroup emotions/reconciliation and emotion regulation. This is does not seem to be directly applicable to CCCs at least in the context presented - 2)**
Putting emotion regulation in context: The (missing) role of power relations, intergroup trust, and groups' need for positive identities in reconciliation processes. [References].
Shnabel, Nurit; Ullrich, Johannes.
Psychological Inquiry. Vol.27(2), 2016, pp. 124-132.
[Journal; Peer Reviewed Journal]
Year of Publication
2016
Publication Month/Season
Apr

Comments on an article by Sabina Cehajic-Clancy, Amit Goldenberg, James J. Gross, and Eran Halperin (see record 2016-21477-001). The target article by Clancy et al. presents an innovative theoretical synthesis of the literature on intergroup emotions and on emotion regulation. Focusing on the appraisal phase of the modal model of emotion offer a comprehensive review of social-psychological interventions, the goal of which is to regulate group members' emotions in the interest of intergroup reconciliation. In the target article, Clancy et al. define reconciliation as the "postconflict resolution process of removing psychological barriers such as negative emotions and beliefs about former/current enemy groups with the goal of creating or restoring positive and sustainable intergroup relations" a definition borrowed from work on interpersonal reconciliation. Clancy et al. conclude in their target article that "discussions with a focus on the past marked by conflict might inhibit potentially positive consequences of contact or perspective-taking due to specific frustrated emotional needs related to the past". The commentators agree with Clancy et al. that research on the role of emotion regulation in reconciliation processes is highly important.

15. **INCLUDE IN ARTICLE REVIEW (coded)**
Transformational leadership and horizontal trust as antecedents of team performance in the healthcare context. [References].
Olvera, Juana; Llorens, Susana; Acosta, Hedy; Salanova, Marisa.
Anales de Psicologia. Vol.33(2), 2017, pp. 365-375.
[Journal; Peer Reviewed Journal]
Year of Publication
2017
Publication Month/Season
May

The present study analyzes the mediator role of work-team trust (i.e., horizontal trust) in the relationship between transformational leadership, as a social resource, and team performance (i.e., intra- and extra-role performance), as suggested by the HERO model (HEalthy & Resilient Organizations Model; Salanova, Llorens, Cifre, & Martinez, 2012). The sample corresponds to 388 workers nested in 54 work teams from four organizations in the healthcare sector. Horizontal trust and transformational leadership were assessed by the work teams, and performance was assessed by the supervisors of these teams. Structural Equations models reveal, as expected, that horizontal trust has a fully mediating role between transformational leadership perceived at the team level and team performance assessed by the supervisor.

17. **EXCLUDE (Studies on team trust in a coach and performance seems a bit of a stretch for CCCs - 2)**
When trust in the leader matters: The moderated-mediation model of team performance and trust. [References].
Mach, Merce; Lvina, Elena.
Journal of Applied Sport Psychology. Vol.29(2), 2017, pp. 134-149.
[Journal; Peer Reviewed Journal]
Year of Publication
2017
Publication Month/Season
Apr

This study contributes to the sport and team literature by exploring the conditions in which trust in a leader translates into trust in a team and subsequent team performance. Findings from 709 athletes on 74 basketball teams demonstrated that trust in the coach represents a critical antecedent of team trust, especially when the team's past performance has been poor. We also found a combined effect of the level and consensus in trust on team performance. Practical implications suggest that a coach needs to ensure that every player, rather than just some or even the majority of individual team members, trusts him or her and the team.

18. **INCLUDE IN ARTICLE REVIEW (coded)**
Political skill, trust, and efficacy in teams. [References].
Lvina, Elena; Maher, Liam P; Harris, John N.
Journal of Leadership & Organizational Studies. Vol.24(1), 2017, pp. 95-105.
[Journal; Peer Reviewed Journal]
Year of Publication
2017
Publication Month/Season
Feb

Political skill, frequently understood as a social skill at work, is argued to be a valuable resource not only at the individual level but also for the teams. Using hierarchical linear modeling and data from 525 students, organized into 115 teams, we demonstrate that political skill at the individual level shapes individual perceptions of team efficacy and trust in team. Both the level and the composition of political skill within the team are found to be critical for these team emergent states, albeit they play out differently for team members who are high versus low in political skill.

20. **EXCLUDE (This study looks at global virtual teams with at least 2 distinct subgroups. This is not likely to be applicable to CCCs - 7)**
Global virtual team performance: The effect of coordination effectiveness, trust, and team cohesion. [References].
Paul, Ravi; Drake, John R; Liang, Huigang.
IEEE Transactions on Professional Communication. Vol.59(3-4), 2016, pp. 186-202.
[Journal; Peer Reviewed Journal]
Year of Publication
2016
Publication Month/Season
Sep-Dec

Research problem: Subgroup formation in global virtual teams could negatively impact team performance due to difficulties in coordination, trust, and team cohesion. Research questions: What role do trust and team cohesion play in the relationship between coordination effectiveness and team performance of global virtual teams with two distinct subgroups? Literature review: Prior research suggests that coordination effectiveness on team performance is most strongly impacted by coordination of knowledge. This effectiveness is mediated by trust and team cohesion. However, we have a poor understanding of trust and team cohesion dynamics on intergroup relationships in global virtual teams. Methodology: A survey was conducted with 14 teams with a total of 112 participants in the US and India. The teams were tasked with evaluating customer-relationship-management best practices for a global environment. Results and discussion: We evaluated how the process of effective coordination for teams composed of two colocated subgroups is mediated by individual perceptions of out-group trust and overall team cohesion. Our findings show that individual trust and team cohesion share a reciprocal impact on each other, suggesting that effective coordination in virtual teams can create a positive feedback loop with trust and cohesion, improving overall project performance. Implications for theory and practice include the virtuous cycle that trust and cohesion create in global virtual team coordination and the necessity of establishing appropriate project coordination systems and processes to promote both aspects and, thus, achieve excellent project performance for colocated subgroup.

21. **INCLUDE IN ARTICLE REVIEW (This abstract was reviewed on the PUBMED document)**
The role of trust in CenteringPregnancy: Building interpersonal trust relationships in group-based prenatal care in the Netherlands. [References].
Kweekel, Liselotte; Gerrits, Trudie; Rijnders, Marlies; Brown, Patrick.
Birth: Issues in Perinatal Care. Vol.44(1), 2017, pp. 41-47.
[Journal; Peer Reviewed Journal]
Year of Publication
2017
Publication Month/Season
Mar

23. **EXCLUDE (This abstract was reviewed on the SCOPUS document)**
A trust induced recommendation mechanism for reaching consensus in group decision making. [References].
Liu, Yujia; Liang, Changyong; Chiclana, Francisco; Wu, Jian.
Knowledge-Based Systems. Vol.119 2017, pp. 221-231.
[Journal; Peer Reviewed Journal]
Year of Publication
2017
Publication Month/Season
Mar

26. **EXCLUDE (Social comparison processes and perceptions of performance in virtual teams does not really apply to CCCs - 7)**
How differences in perceptions of own and team performance impact trust and job satisfaction in virtual teams. [References].
Romeike, Philipp Daniel; Nienaber, Ann-Marie; Schewe, Gerhard.
Human Performance. Vol.29(4), 2016, pp. 291-309.
[Journal; Peer Reviewed Journal]
Year of Publication
2016
Publication Month/Season
Aug

Employees frequently engage in social comparison processes and tend to perceive their own performance as superior compared to that of their peers. We expect this to be particularly salient in virtual teams where employees receive few cues upon which the comparison with other members of their team can be based. With reliance on social comparison and social exchange theory, we propose that such "perceived overperformance" has negative effects on job satisfaction, which is mediated by trust in the team. We confirm this with a sample of field-service employees (n = 753) using structural equation modeling with bootstrapping. We corroborated our findings in focus groups, which suggest the need for performance indicators that are easily communicated to and comprehended by employees to maintain trust and satisfaction.

28. **EXCLUDE (The Olson and Putnam hypotheses discussed in this article do not really apply to CCCs, or at least it would be a stretch to correlate this to an HPE setting - 2).**
Groups and trust: Experimental evidence on the Olson and Putnam hypotheses. [References].
Antoni, Giacomo Degli; Grimalda, Gianluca.
Journal of Behavioral and Experimental Economics. Vol.61 2016, pp. 38-54.
[Journal; Peer Reviewed Journal]
Year of Publication
2016
Publication Month/Season
Apr

Mancur Olson and Robert Putnam provide two conflicting views on the effect of involvement with voluntary associations on their members. Putnam argues that associations instill in their members habits of cooperation, solidarity and public spiritedness. Olson emphasizes the tendency of groups to pursue private interests and lobby for preferential policies. We carry out the first field experiment involving a sample of members of different association types from different age groups and education levels, as well as a demographically comparable sample of non-members. This enables us to examine the differential patterns of behavior followed by members of Putnam-type and Olson-type associations. Coherently with both the Putnam's and Olson's view, we find that members of Putnam-type (Olson-type) associations display more (no more) generalized trust than non-members. However, when we examine trustworthy behavior we find the opposite pattern, with members of Olson-type (Putnam-type) associations more (no more) trustworthy than non-members. No systematic effect for the intensity of participation in associations emerges. We analyze the issue of self-selection through a structural equation model. This supports the view that membership has a significant effect on prosociality.

29. **EXCLUDE (Although this is an interesting study and one that may be useful to look at, I think the fact that it was conducted on work groups in a manufacturing plant in China limit generalizability. I think the occupational setting and cultural differences make the results less applicable to CCCs - 2).**
A field examination of the moderating role of group trust in group efficacy formation. [References].
Lee, Dongseop; Stajkovic, Alexander D; Sergent, Kayla.
Journal of Occupational and Organizational Psychology. Vol.89(4), 2016, pp. 856-876.
[Journal; Peer Reviewed Journal]
Year of Publication
2016
Publication Month/Season
Dec

Although support for the positive effect of group efficacy on group performance is copious, our understanding of how group efficacy forms is scant. Much remains unanswered about how the four efficacy antecedents, defined by social cognitive theory as enacted mastery, vicarious learning, social persuasion, and affect, concurrently influence group efficacy. Complementing the homology assumption of social cognitive theory, the authors propose and test a differential moderation model of group trust, in which group trust interacts synergistically or compensatorily with the four antecedents of group efficacy depending on the information content they provide. The results, based on 100 work groups in a manufacturing plant in China, showed that past group performance and group social persuasion were positively related to group efficacy, but that group vicarious learning and group positive affect were not. A significant interaction with group trust was found for group vicarious learning and for group positive affect. Finally, group efficacy was positively related to subsequent group performance, even after controlling for the four efficacy antecedents and group trust. Theoretical and practical implications are discussed.

30. **INCLUDE IN ARTICLE REVIEW (This abstract was reviewed on the SCOPUS document)**
In small we trust: Lay theories about small and large groups. [References].
La Macchia, Stephen T; Louis, Winnifred R; Hornsey, Matthew J; Leonardelli, Geoffrey J.
Personality and Social Psychology Bulletin. Vol.42(10), 2016, pp. 1321-1334.
[Journal; Peer Reviewed Journal]
Year of Publication
2016
Publication Month/Season
Oct

31. **EXCLUDE (This abstract was reviewed on the SCOPUS document)**
Building trust in a postconflict society: An integrative model of cross-group friendship and intergroup emotions. [References].
Kenworthy, Jared Bowden; Voci, Alberto; Ramiah, Ananthi Al; Tausch, Nicole; Hughes, Joanne; Hewstone, Miles.
Journal of Conflict Resolution. Vol.60(6), 2016, pp. 1041-1070.
[Journal; Peer Reviewed Journal]
Year of Publication
2016
Publication Month/Season
Sep

32. **EXCLUDE (I think we have other studies selected that cover this topic. This article looks at incubator-based entrepreneurial teams in Austria, which limits applicability and generalizability to CCCs - 2).**
Innovative entrepreneurial teams: The give and take of trust and conflict. [References].
Khan, Mohammad Saud; Breitenecker, Robert J; Gustafsson, Veronika; Schwarz, Erich J.
Creativity and Innovation Management. Vol.24(4), 2015, pp. 558-573.
[Journal; Peer Reviewed Journal]
Year of Publication
2015
Publication Month/Season
Dec

Entrepreneurship research lacks empirical evidence about interactions between entrepreneurial team members. This paper examines the role of trust (cognitive and affective) and conflict (task and relationship) on team performance (effectiveness and efficiency) of innovative entrepreneurial teams. Data originated from 88 incubator-based entrepreneurial teams in Austria. Results indicate that cognitive trust is the cornerstone of innovative entrepreneurial team performance. To maximize efficiency, such teams would rely on high cognitive trust and low task conflict. Nonetheless, the guidelines for being effective centre around high cognitive trust coupled with low task and relationship conflict.

33. **INCLUDE IN ARTICLE REVIEW (coded)**
Trust and team performance: A meta-analysis of main effects, moderators, and covariates. [References].
De Jong, Bart A; Dirks, Kurt T; Gillespie, Nicole.
Journal of Applied Psychology. Vol.101(8), 2016, pp. 1134-1150.
[Journal; Peer Reviewed Journal]
Year of Publication
2016
Publication Month/Season
Aug

Cumulating evidence from 112 independent studies (N = 7,763 teams), we meta-analytically examine the fundamental questions of whether intrateam trust is positively related to team performance, and the conditions under which it is particularly important. We address these questions by analyzing the overall trust-performance relationship, assessing the robustness of this relationship by controlling for other relevant predictors and covariates, and examining how the strength of this relationship varies as a function of several moderating factors. Our findings confirm that intrateam trust is positively related to team performance, and has an above-average impact (rho = .30). The covariate analyses show that this relationship holds after controlling for team trust in leader and past team performance, and across dimensions of trust (i.e., cognitive and affective). The moderator analyses indicate that the trust-performance relationship is contingent upon the level of task interdependence, authority differentiation, and skill differentiation in teams. Finally, we conducted preliminary analyses on several emerging issues in the literature regarding the conceptualization and measurement of trust and team performance (i.e., referent of intrateam trust, dimension of performance, performance objectivity). Together, our findings contribute to the literature by helping to (a) integrate the field of intrateam trust research, (b) resolve mixed findings regarding the trust-performance relationship, (c) overcome scholarly skepticism regarding the main effect of trust on team performance, and (d) identify the conditions under which trust is most important for team performance.

34. **INCLUDE IN ARTICLE REVIEW (waiting to code - Virtual Teams - 7)**
Does trust matter more in virtual teams? A meta-analysis of trust and team effectiveness considering virtuality and documentation as moderators. [References].
Breuer, Christina; Huffmeier, Joachim; Hertel, Guido.
Journal of Applied Psychology. Vol.101(8), 2016, pp. 1151-1177.
[Journal; Peer Reviewed Journal]
Year of Publication
2016
Publication Month/Season
Aug

Team trust has often been discussed both as requirement and as challenge for team effectiveness, particularly in virtual teams. However, primary studies on the relationship between trust and team effectiveness have provided mixed findings. The current review summarizes existing studies on team trust and team effectiveness based on meta-analytic methodology. In general, we assumed team trust to facilitate coordination and cooperation in teams, and therefore to be positively related with team effectiveness. Moreover, team virtuality and documentation of interactions were considered as moderators of this relationship because they should affect perceived risks during teamwork. While team virtuality should increase, documentation of interaction should decrease the relationship between team trust and team effectiveness. Findings from 52 studies with 54 independent samples (representing 12,615 individuals in 1,850 teams) confirmed our assumptions. In addition to the positive overall relationship between team trust and team effectiveness criteria (rho = .33), the relationship between team trust and team performance was stronger in virtual teams (rho = .33) as compared to face-to-face teams (rho = .22), and weaker when team interactions were documented (rho = .20) as compared to no such documentation (rho = .29). Thus, documenting team interactions seems to be a viable complement to trust-building activities, particularly in virtual teams.

35. **INCLUDE IN ARTICLE REVIEW (EXCLUDE after review of the full text - 11 July 2018. This article focuses on employees working on R&D teams for a large IT company in China and looks at functional diversity with an outcome of team innovation. The cultural/work differences and outcomes have minimal overlap with CCCs).**
When and how does functional diversity influence team innovation? The mediating role of knowledge sharing and the moderation role of affect-based trust in a team. [References].
Cheung, Siu Yin; Gong, Yaping; Wang, Mo; Zhou, Le (Betty); Shi, Junqi.
Human Relations. Vol.69(7), 2016, pp. 1507-1531.
[Journal; Peer Reviewed Journal]
Year of Publication
2016
Publication Month/Season
Jul

Findings from prior research on the relationship between functional diversity and team innovation have been inconclusive. This study aims to reconcile the mixed findings in the literature by investigating how functional diversity may influence team innovation and when such influence may or may not occur. The view of teams as information processors suggests that functionally diverse teams may capitalize on their knowledge benefits to produce innovations through knowledge sharing. However, knowledge sharing and subsequent team innovation do not necessarily occur in functionally diverse teams. Drawing on the motivated information processing in groups theory, we propose that affect-based trust in a team moderates the effects of functional diversity on team innovation (via knowledge sharing). The results based on a sample of 96 research and development teams indicate that functional diversity had a negative indirect relationship with team innovation via knowledge sharing when affect-based trust in a team was low, and this relationship became less negative as the level of affect-based trust in a team increased. The relationship was not significant when affect-based trust in a team was high.

36. **EXCLUDE (The population studied and “interactional justice climate” does not really seem applicable or readily transferable to CCCs - 2)**
The moderating role of performance in the link from interactional justice climate to mutual trust between managers and team members. [References].
Martinez-Tur, Vicente; Gracia, Esther; Moliner, Carolina; Molina, Agustin; Kuster, Ines; Vila, Natalia; Ramos, Jose.
Psychological Reports. Vol.118(3), 2016, pp. 870-888.
[Journal; Peer Reviewed Journal]
Year of Publication
2016
Publication Month/Season
Jun

The main goal of this study was to examine the interaction between team members' performance and interactional justice climate in predicting mutual trust between managers and team members. A total of 93 small centers devoted to the attention of people with intellectual disability participated in the study. In each center, the manager (N = 93) and a group of team members (N = 746) were surveyed. On average, team members were 36.2 years old (SD = 9.3), whereas managers were 41.2 years old (SD = 8.8). The interaction between interactional justice climate and performance was statistically significant. Team members' performance strengthened the link from interactional justice climate to mutual trust.

37. **EXCLUDE (This study looks at multicultural and unicultural semi-virtual collaboration teams. Although this may have some applicability to CCCs, I have a feeling this study is more focused on computer science given the journal in which it’s published - 7).**
Investigating individual trust in semi-virtual collaboration of multicultural and unicultural teams. [References].
Cheng, Xusen; Fu, Shixuan; Sun, Jianshan; Han, Yajing; Shen, Jia; Zarifis, Alex.
Computers in Human Behavior. Vol.62 2016, pp. 267-276.
[Journal; Peer Reviewed Journal]
Year of Publication
2016
Publication Month/Season
Sep

This study aims to investigate individual's trust development for semi-virtual collaboration teams with multicultural and unicultural background. We aim to explore whether the trust levels in multicultural and unicultural semi-virtual groups will be the same, how trust develops over time and what the corresponding factors to the trust development are. In order to answer the questions, a longitudinal case study was conducted in unicultural and multicultural teams. We have taken survey for 144 participants over three stages, as well as interviewed 64 participants. Results of the analysis of the survey data firstly show that no significant difference exists between multicultural and unicultural groups. Then, two factors, collaboration process and clear task help explain this phenomenon. However, the trust development of multicultural groups shows instability and keeps decreasing over time, while unicultural groups behave differently. We found that language, values and habitual behavior lead to the differences in these two types of groups.

38. **EXCLUDE (This article looks at age in the setting of dyadic relationships with clients, the concept of boundary spanners, and the population studied is the consulting industry. I don’t think this article is readily transferable or applicable to CCCs - 4)**
Being trusted: How team generational age diversity promotes and undermines trust in cross-boundary relationships. [References].
Williams, Michele.
Journal of Organizational Behavior. Vol.37(3), 2016, pp. 346-373.
[Journal; Peer Reviewed Journal]
Year of Publication
2016
Publication Month/Season
Apr

We examine how demographic context influences the trust that boundary spanners experience in their dyadic relationships with clients. Because of the salience of age as a demographic characteristic as well as the increasing prevalence of age diversity and intergenerational conflict in the workplace, we focus on team age diversity as a demographic social context that affects trust between boundary spanners and their clients. Using social categorization theory and theories of social capital, we develop and test our contextual argument that a boundary spanner's experience of being trusted is influenced by the social categorization processes that occur in dyadic interactions with a specific client and, simultaneously, by similar social categorization processes that influence the degree to which the client team as a whole serves as a cooperative resource for demographically similar versus dissimilar boundary spanner-client dyads. Using a sample of 168 senior boundary spanners from the consulting industry, we find that generational diversity among client team members from a client organization undermines the perception of being trusted within homogeneous boundary spanner-client dyads while it enhances the perception of being trusted within heterogeneous dyads. The perception of being trusted is an important aspect of cross-boundary relationships because it influences coordination and the costs associated with coordination.

39. **EXCLUDE (The population studied and the concepts of intergroup anxiety and group status doesn’t transfer readily to CCCs - 2)**
From cultural discordance to support for collective action: The roles of intergroup anxiety, trust, and group status. [References].
Brylka, Asteria; Mahonen, Tuuli Anna; Schellhaas, Fabian M. H; Jasinskaja-Lahti, Inga.
Journal of Cross-Cultural Psychology. Vol.46(7), 2015, pp. 897-915.
[Journal; Peer Reviewed Journal]
Year of Publication
2015
Publication Month/Season
Aug

Support for collective action, an initial step in challenging social inequalities, can have important implications for intergroup relations in contemporary plural societies. The present study, conducted among majority Finns (n = 274) and Russian immigrants in Finland (n = 228), investigated the relationship between perceived cultural discordance (i.e., disagreement between majority and minority groups about the importance of cultural maintenance for minority members) and support for collective action benefiting the minority. Specifically, it was expected that the association between cultural discordance and collective action would be at least partially mediated by intergroup anxiety and outgroup trust, and that both the direct and indirect effects of cultural discordance would depend on group status (i.e., majority vs. minority). The results showed that among majority group members, the association was negative and mediated by high intergroup anxiety and low outgroup trust, whereas among minority group members, the association was positive and mediated by only low outgroup trust.

42. **EXCLUDE (Although diversity and may apply in a CCC setting, the statistical methods used in this study and that fact that it looks at cross-cultural students’ work projects may limit the applicability to CCCs - 4)**
Team performance in cross cultural project teams: The moderated mediation role of consensus, heterogeneity, faultlines and trust. [References].
Mach, Merce; Baruch, Yehuda.
Cross Cultural Management. Vol.22(3), 2015, pp. 464-486.
[Journal; Peer Reviewed Journal]
Year of Publication
2015

Purpose: The purpose of this paper is to test the conditional effect of team composition on team performance; specifically, how collective team orientation, group consensus, faultline configurations and trust among team members explain the objective performance of project teams in cross-cultural contexts. Design/methodology/approach: Employing path analytical framework and bootstrap methods, the authors analyze data from a sample of 73 cross cultural project teams. Relying on ordinary least-squares regression, the authors estimate the direct and indirect effects of the moderated mediation model. Findings: The findings demonstrate that the indirect effect of collective team orientation on performance through team trust is moderated by team member consensus, diversity heterogeneity and faultlines' strength. By contrast, high dispersion among members, heterogeneous team configurations and strong team faultlines lead to low levels of trust and team performance. Research limitations/implications: The specific context of the study (cross-cultural students' work projects) may influence external validity and limit the generalization of the findings as well as the different compositions of countries-of-origin. Practical implications: From a practical standpoint, these results may help practitioners understand how the emergence of trust contributes to performance. It will also help them comprehend the importance of managing teams while bearing in mind the cross-cultural contexts in which they operate. Social implications: In order to foster team consensus and overcome the effects of group members' cross-cultural dissimilarities as well as team faultlines, organizations should invest in improving members' dedication, cooperation and trust before looking to achieve significant results, specially in heterogeneous teams and cross-cultural contexts. Originality/value: The study advances organizational group research by showing the combined effect of team configurations and collective team orientation to overall team performance and by exploring significant constructs such as team consensus, team trust and diversity faultline strength to examine their possible moderated mediation role in the process.

43. **EXCLUDE (This abstract was reviewed on the SCOPUS document)**
Communication and trust are key: Unlocking the relationship between leadership and team performance and creativity. [References].
Boies, Kathleen; Fiset, John; Gill, Harjinder.
The Leadership Quarterly. Vol.26(6), 2015, pp. 1080-1094.
[Journal; Peer Reviewed Journal]
Year of Publication
2015
Publication Month/Season
Dec

44. **INCLUDE IN ARTICLE REVIEW (waiting to code - Virtual Teams - 7)**
Trust evolvement in hybrid team collaboration: A longitudinal case study. [References].
Cheng, Xusen; Yin, Guopeng; Azadegan, Aida; Kolfschoten, Gwendolyn.
Group Decision and Negotiation. Vol.25(2), 2016, pp. 267-288.
[Journal; Peer Reviewed Journal]
Year of Publication
2016
Publication Month/Season
Mar

Trust is referred to as a key facilitator in team collaboration as it is an important condition for information sharing. In this paper, we investigate factors associated with the establishment of trust in hybrid teams that collaborate virtually as well as face-to-face. Furthermore, we deliver an instrument to understand trust development in teams. We describe exploratory results of the instrument by running experiments with teams of collaborating students in China and Netherlands. Quantitative and qualitative analysis has been used to analyze these data. Finally, in the analysis of the experiments we describe initial patterns of trust development in groups from both individual and group perspectives, in two different cultural contexts.

45. **INCLUDE IN ARTICLE REVIEW (coded)**
Trust in building high-performing teams-Conceptual approach. [References].
Hakanen, Mila; Hakkinen, Mia; Soudunsaari, Aki.
Electronic Journal of Business Ethics and Organization Studies. Vol.20(2), 2015, pp. 43-53.
[Journal; Peer Reviewed Journal]
Year of Publication
2015

Team building is one of the key factors of success in business. This study highlights the key elements of building winning teams, where trust is one essential building block and top-level sports teams serve as instructive examples. This study highlights earlier findings that revealed a strong connection between trust and high-performing teams. High-performing teams need talented people but also constant trust-based cooperation. Trust supports cooperative behaviour. Studies of top-level sports teams also emphasize the role of trust when building high-performing teams. This study is implemented using conceptual research in order to organize information related to the complex phenomenon of high-performing teams. The conceptual approach was chosen instead of empirical research due the lack of prior holistic research into high-performing teams including perspectives on trust and sports teams. A theoretical framework is proposed by analysing key characteristics and defining high-performing teams and related concepts. The framework highlights dimensions such as clear vision, trust and communication. Team member skill, motivation and responsibilities were highlighted, and respect and support were also addressed. Leadership is a critical dimension that includes clear roles, standards and goals. The proposed framework for building high-performing teams offers the basis for subsequent empirical research.

46. **INCLUDE IN ARTICLE REVIEW (EXCLUDE after review of the full text - 12 July 2018. This article focuses on the relationship between individual affective traits and prosocial behavior. Although trust is a considered a moderator in this relationship, the outcome of prosocial behavior seems like a construct that is of little consequence to CCCs in comparison to several other outcomes. - 2)**
Affective balance, team prosocial efficacy and team trust: A multilevel analysis of prosocial behavior in small groups. [References].
Cuadrado, Esther; Tabernero, Carmen.
PLoS ONE. Vol.10(8), 2015, ArtID e0136874.
[Journal; Peer Reviewed Journal]
Year of Publication
2015
Publication Month/Season
Aug

Little research has focused on how individual- and team-level characteristics jointly influence, via interaction, how prosocially individuals behave in teams and few studies have considered the potential influence of team context on prosocial behavior. Using a multilevel perspective, we examined the relationships between individual (affective balance) and group (team prosocial efficacy and team trust) level variables and prosocial behavior towards team members. The participants were 123 students nested in 45 small teams. A series of multilevel random models was estimated using hierarchical linear and nonlinear modeling. Individuals were more likely to behave prosocially towards in-group members when they were feeling good. Furthermore, the relationship between positive affective balance and prosocial behavior was stronger in teams with higher team prosocial efficacy levels as well as in teams with higher team trust levels. Finally, the relevance of team trust had a stronger influence on behavior than team prosocial efficacy.

48. **INCLUDE IN ARTICLE REVIEW (This abstract was reviewed on the ERIC document)**
The importance of trust for satisfaction, motivation, and academic performance in student learning groups. [References].
Ennen, Nicole L; Stark, Emily; Lassiter, Andrea.
Social Psychology of Education. Vol.18(3), 2015, pp. 615-633.
[Journal; Peer Reviewed Journal]
Year of Publication
2015
Publication Month/Season
Sep

49. **EXCLUDE (This article looks at a moderated mediation model, team feedback, and team learning in a virtual setting. This study of virtual teams seems less applicable to CCCs than some of the other studies we have included - 7)**
How team feedback and team trust influence information processing and learning in virtual teams: A moderated mediation model. [References].
Penarroja, Vicente; Orengo, Virginia; Zornoza, Ana; Sanchez, Jesus; Ripoll, Pilar.
Computers in Human Behavior. Vol.48 2015, pp. 9-16.
[Journal; Peer Reviewed Journal]
Year of Publication
2015
Publication Month/Season
Jul

This study examines a moderated mediation model in which team trust moderates the indirect effect of team feedback on team learning through group information elaboration in virtual teams. An experimental study in a laboratory was conducted with 54 teams randomly assigned to a team feedback condition or a control condition. Results provided empirical support to the moderated mediation model. We found that the indirect effect of team feedback on team learning via group information elaboration occurred in virtual teams with a high level of team trust. However, this indirect effect was not statistically significant in virtual teams with lower levels of team trust. Additionally, we also found that group information elaboration and team learning were positively related in virtual teams. Therefore, our findings suggest that team feedback is effective to improve group information elaboration and learning in virtual teams when team trust is high.

50. **EXCLUDE (This abstract was reviewed on the SCOPUS document)**
Social cues of (un)trustworthy team members. [References].
Neu, Wayne A.
Journal of Marketing Education. Vol.37(1), 2015, pp. 36-53.
[Journal; Peer Reviewed Journal]
Year of Publication
2015
Publication Month/Season
Apr

51. **EXCLUDE (This article presents some complicated concepts including trust congruence, task and contextual performance, work group motivation and mediated relationships that make the applicability to CCCs unclear - 4).**
Are we on the same page? The performance effects of congruence between supervisor and group trust. [References].
Carter, Min Z; Mossholder, Kevin W.
Journal of Applied Psychology. Vol.100(5), 2015, pp. 1349-1363.
[Journal; Peer Reviewed Journal]
Year of Publication
2015
Publication Month/Season
Sep

Taking a multiple-stakeholder perspective, we examined the effects of supervisor-work group trust congruence on groups' task and contextual performance using a polynomial regression and response surface analytical framework. We expected motivation experienced by work groups to mediate the positive influence of trust congruence on performance. Although hypothesized congruence effects on performance were more strongly supported for affective rather than for cognitive trust, we found significant indirect effects on performance (via work group motivation) for both types of trust. We discuss the performance effects of trust congruence and incongruence between supervisors and work groups, as well as implications for practice and future research.

53. **INCLUDE IN ARTICLE REVIEW (coded)**
Sinking slowly: Diversity in propensity to trust predicts downward trust spirals in small groups. [References].
Ferguson, Amanda J; Peterson, Randall S.
Journal of Applied Psychology. Vol.100(4), 2015, pp. 1012-1024.
[Journal; Peer Reviewed Journal]
Year of Publication
2015
Publication Month/Season
Jul

This paper examines the phenomenon of trust spirals in small groups. Drawing on literature on the spiral reinforcement of trust, we theorize that diversity in propensity to trust has affective and cognitive consequences related to trust (i.e., feelings of frustration and perceptions of low similarity), reducing the level of experienced intragroup trust early in a group's development. Reduced experienced trust then fuels relationship conflict and lowers trust even further over time, ultimately having a negative effect on group performance. These ideas are tested using a sample of MBA student groups surveyed at 3 time periods over 4 months. Results confirm our hypothesis that diversity in propensity to trust is sufficient to trigger a downward trust spiral and poor performance in small groups.

54. **EXCLUDE (This abstract was reviewed on the SCOPUS document)**
Is trust always better than distrust? The potential value of distrust in newer virtual teams engaged in short-term decision-making. [References].
Lowry, Paul Benjamin; Schuetzler, Ryan M; Giboney, Justin Scott; Gregory, Thomas A.
Group Decision and Negotiation. Vol.24(4), 2015, pp. 723-752.
[Journal; Peer Reviewed Journal]
Year of Publication
2015
Publication Month/Season
Jul

58. **EXCLUDE (The population studied and the idea of internal locus of control don’t readily transfer or apply to CCCs - 2)**
Entrepreneurial team locus of control: Diversity and trust. [References].
Khan, Mohammad Saud; Breitenecker, Robert J; Schwarz, Erich J.
Management Decision. Vol.52(6), 2014, pp. 1057-1081.
[Journal; Peer Reviewed Journal]
Year of Publication
2014

Purpose - The purpose of this paper is to examine how internal locus of control (LOC) as a well-established entrepreneurial personality trait at team level impacts team performance (effectiveness and efficiency) in Austria. In addition, it investigates the interaction effects of LOC diversity and affective trust on the internal LOC-performance relationship. Design/methodology/approach - Data originated from 44 entrepreneurial teams based in nine business incubators in Austria. Partial least squares (PLS) structural equation modelling was used to estimate the model. Findings - Results indicate that higher internal LOC at team level promotes entrepreneurial team effectiveness and efficiency. However, team efficiency is increased when such teams possess a high internal LOC and low LOC diversity. Affective trust is identified as a crucial component in enhancing entrepreneurial team effectiveness, especially when the team has a high internal LOC. Originality/value - This study extends research on internal LOC at team level by investigating it as a predictor of entrepreneurial team effectiveness and efficiency. Second, it systematically analyses if and how diversity in internal LOC affects team performance in an entrepreneurial team context. The paper takes a pioneering step by testing a key methodological contribution of addressing the inherent bias in measuring diversity of small teams. Finally, it is one of the first studies to show not only the importance of affect in general, but also the trust based on affect for entrepreneurial team dynamics.

59. **EXCLUDE (This study looks at virtual teams in a multinational company in Malaysia, which limits applicability to CCCs - 7)**
The mediating effect of knowledge sharing on the relationship between trust and virtual team effectiveness. [References].
Pangil, Faizuniah; Chan, Joon Moi.
Journal of Knowledge Management. Vol.18(1), 2014, pp. 92-106.
[Journal; Peer Reviewed Journal]
Year of Publication
2014

Purpose - The limited proximal communication between virtual team members can create a lack of trust among members which can significantly reduce the effectiveness of these teams. Hence, this study was conducted to examine the relationship between trust and virtual team effectiveness by looking into the mediating effect of knowledge sharing. Design/methodology/approach - This is a cross-sectional study conducted in a multinational company in Malaysia. Questionnaires were distributed to individuals working in a virtual environment. The questionnaire required the respondents to answer questions regarding three different types of trust (i.e. personality-based trust, institutional-based trust and cognitive-based trust), their knowledge sharing behavior, and their evaluation of the effectiveness of the virtual teams that they are involved with. Findings - Results of hierarchical regression analysis indicated that knowledge sharing and all the three types of trust are significantly related to virtual team effectiveness. However, only personality-based trust and institutional-based trust are significantly related to knowledge sharing, but knowledge sharing only partially mediates the relationship between these two types of trust and team effectiveness. Research limitations/implications - The population of this study are virtual teams working for an organization, hence the generalizability of the findings to other settings is unknown. Originality/value - Trust has been founded to be a significant predictor of virtual team effectiveness. However, this paper shows the effect of different types of trust and the role of knowledge sharing in mediating the relationship between trust and virtual team effectiveness.

61. **EXCLUDE (This study looks at trust games, and has a focus on economics, which limits the applicability to CCCs - 7)**
Group status, minorities and trust. [References].
Tsutsui, Kei; Zizzo, Daniel John.
Experimental Economics. Vol.17(2), 2014, pp. 215-244.
[Journal; Peer Reviewed Journal]
Year of Publication
2014
Publication Month/Season
Jun

We present the results of an experiment measuring the impact of low group status and relative group size on trust, trustworthiness and discrimination. Subjects interact with insiders and outsiders in trust games and periodically enter markets where they can trade group membership. Low status and minority subjects have low morale: that is, they comparatively dislike being low status and being minority subjects. Group discrimination against low status and minority subjects is unchanged. However, low status subjects are deferential to high status subjects in terms of comparatively higher trust, and minority subjects are deferential to majority subjects in terms of comparatively higher trustworthiness.

62. **EXCLUDE (This study of virtual teams seems less applicable to CCCs than some of the other studies we have included, especially given the fact that it likely looks at the marketing industry - 7)**
The impact of trust on virtual team effectiveness. [References].
El-Kassrawy, Yasser A.
International Journal of Online Marketing. Vol.4(1), 2014, pp. 11-28.
[Journal; Peer Reviewed Journal]
Year of Publication
2014

Given the important role of information technology, virtuality has become crucial issue in contemporary organizations. Virtual teams are comprised of members who are located in more than one physical location. They need to be effectively collaborating to harness their full performance capabilities in order to compete in the highly competitive environments. However, virtual team effectiveness is affected by determinants of trust which include three types; personality, cognitive and institutional-based trust. Therefore, this paper examines the impact of trust determinants on virtual team effectiveness represented in virtual team satisfaction and performance. Through a survey of 125 virtual team members who had experienced at least two years in this field, the results indicated that determinants of trust positively influence virtual team satisfaction and virtual team performance. The authors' structural equations modeling findings also support our hypothetical predictions that personality- based trust, cognitive- based trust and institutional- based trust have a dramatic impact on both of virtual team satisfaction and virtual team performance. Moreover, institutional- based trust is the uppermost driver of virtual team effectiveness. This study provides novel insights into virtual team behaviours, managerial and research implications for effective virtual team.

63. **EXCLUDE (This study looks at multisource feedback and trust in leaders, not the group - 4)**
A new approach to analyzing the Achilles' heel of multisource feedback programs: Can we really trust ratings of leaders at the group level of analysis? [References].
Markham, Steven E; Smith, Janice Witt; Markham, Ina S; Braekkan, Kristian F.
The Leadership Quarterly. Vol.25(6), 2014, pp. 1120-1142.
[Journal; Peer Reviewed Journal]
Year of Publication
2014
Publication Month/Season
Dec

This research addresses the Achilles' heel of all multi-source leadership feedback programs regardless of whether they use 360degree, 270degree, or 180degree ratings. Namely, should all feedback be universally aggregated by feedback groups and reported to respondents? A sample of raters from the Center for Creative Leadership (N = 15,500 including subordinates, peers, and others) was grouped to align with each raters' matching focal subject (J = 1,550). Two scales from CCL's Benchmarks instrument (Building and Mending Relationships and Career Management) were used to: (1) predict a derailment factor (Problems with Interpersonal Relationships) and (2) determine the operative level of analysis and its aggregation characteristics using Within and Between Analysis (WABA). In many cases, the variables under consideration did not aggregate optimally. Using a technique based upon each group's coefficient of variation as a measure of internal consensus, between 3% and 25% of groups should not have their averages reported. Alternative approaches are discussed.

65. **EXCLUDE (Gathering social network data to investigate structural patterns of procedural justice in teams doesn’t align with how we will be looking at CCCs - 4)**
The role of leadership and trust in creating structural patterns of team procedural justice: A social network investigation. [References].
Liu, Dong; Hernandez, Morela; Wang, Lei.
Personnel Psychology. Vol.67(4), 2014, pp. 801-845.
[Journal; Peer Reviewed Journal]
Year of Publication
2014
Publication Month/Season
Win

We adopt a social network perspective to investigate the distinct structural patterns (i.e., centralization and density) of procedural justice (PJ) in teams and the antecedent factors that create them. Across 2 longitudinal field studies in which we gathered social network data from 1,008 workers on 138 teams (Study 1) in China and 672 workers on 125 teams (Study 2) in the United States, we found that differentiation in leader-member exchange relationships significantly influenced the centralization and density of PJ within a team by affecting the level of intrateam trust. Specifically, the more differentiated leader treatment team members received, the lower the level of trust within a team, which resulted in more concentrated (high centralization) and fewer (low density) social interactions among members regarding team PJ. Furthermore, differentiated leader treatment of team members was especially damaging to intrateam trust and, in turn, the structural patterns of team PJ when team members were in close proximity and highly sensitive to equity issues.

71. **INCLUDE IN ARTICLE REVIEW (This abstract was reviewed on the SCOPUS document)**
What makes an effective team? The role of trust (dis)confirmation in team development. [References].
Yang, Inju.
European Management Journal. Vol.32(6), 2014, pp. 858-869.
[Journal; Peer Reviewed Journal]
Year of Publication
2014
Publication Month/Season
Dec

72. **INCLUDE IN ARTICLE REVIEW (coded)**
Psychological ownership, territorial behavior, and being perceived as a team contributor: The critical role of trust in the work environment. [References].
Brown, Graham; Crossley, Craig; Robinson, Sandra L.
Personnel Psychology. Vol.67(2), 2014, pp. 463-485.
[Journal; Peer Reviewed Journal]
Year of Publication
2014
Publication Month/Season
Sum

In this field study, we develop and test a theory regarding the role of trust in the work environment as a critical condition that determines the relationship between psychological ownership, territoriality, and being perceived as a team contributor. We argue that, dependent upon the context of trust in the work environment, psychological ownership may lead to territorial behaviors of claiming and anticipatory defending and that, dependent upon the context of trust, territorial behavior may lead coworkers to negatively judge the territorial employee as less of a team contributor. A sample of working adults reported on their psychological ownership and territorial behavior toward an important object at work, and a coworker of each provided evaluations on the level of trust in the work environment and rated the focal individual's contributions to the team. Findings suggest that a work environment of trust is a "double-edged sword": On the one hand, a high trust environment reduces the territorial behavior associated with psychological ownership; on the other hand, when territorial behavior does occur in high trust environments, coworkers rate the territorial employee's contributions to the team significantly lower. We discuss the nature and management of territorial behavior in light of these findings.

74. **INCLUDE IN ARTICLE REVIEW (waiting to code - Virtual Teams - 7)**
Preventing undesirable effects of mutual trust and the development of skepticism in virtual groups by applying the knowledge and information awareness approach. [References].
Engelmann, Tanja; Kolodziej, Richard; Hesse, Friedrich W.
International Journal of Computer-Supported Collaborative Learning. Vol.9(2), 2014, pp. 211-235.
[Journal; Peer Reviewed Journal]
Year of Publication
2014
Publication Month/Season
Jun

Empirical studies have proven the effectiveness of the knowledge and information awareness approach of Engelmann and colleagues for improving collaboration and collaborative problem-solving performance of spatially distributed group members. This approach informs group members about both their collaborators' knowledge structures and their collaborators' information. In the current study, we investigated whether this implicit approach reduces undesirable effects of mutual trust and mutual skepticism. Trust is an important influencing factor with regard to behavior and performance of groups. High mutual trust can have a negative impact on group effectiveness because it reduces mutual control and, as a result, the detection of the others' mistakes. In an empirical study, 20 triads collaborating with the knowledge and information awareness approach were compared with 20 triads collaborating without this approach. The members of a triad were spatially distributed and participated in a computer-supported collaboration. The results demonstrated that the availability of the knowledge and information awareness approach overrides the negative impact of too much mutual trust and counteracts the development of mutual skepticism. This study contributes to further clarifying the impact of trust on effectiveness and efficiency of virtual groups depending upon different situational contexts.

75. **EXCLUDE (This study of virtual teams seems less applicable to CCCs than some of the other studies we have included, especially given the fact that it likely looks at groups of students - 7)**
Exploring individual trust factors in computer mediated group collaboration: A case study approach. [References].
Cheng, Xusen; Macaulay, Linda.
Group Decision and Negotiation. Vol.23(3), 2014, pp. 533-560.
[Journal; Peer Reviewed Journal]
Year of Publication
2014
Publication Month/Season
May

Trust has become more and more important in the context of mixed use of longitudinal face-to-face and computer mediated group collaboration using Group Support System tools. Previous research has investigated trust factors in different dimensions. This paper takes the perspective of individual trust and aims to explore the new trust factors and also their detailed second level trust sub-factors in computer mediated collaboration over time. We have taken the interviews using the student groups during the two year-long collaboration project based case studies. We have validated the previous factors and found seven new trust factors and thirty one sub-factors which are associated with the main factors. Furthermore, based on the new factors, this paper has also designed an innovative trust traffic light model with suggested steps which could be easily used to help analyze the trust factors development over time for future longitudinal studies.

78. **EXCLUDE (This study looks at gender differences groups with implications in the setting of economic transactions. I think this has limited transferability to CCCs based upon how the economic context of this study - 2)**
Gender differences in trust and trustworthiness: Individuals, single sex and mixed sex groups. [References].
Chaudhuri, Ananish; Paichayontvijit, Tirnud; Shen, Lifeng.
Journal of Economic Psychology. Vol.34 2013, pp. 181-194.
[Journal; Peer Reviewed Journal]
Year of Publication
2013
Publication Month/Season
Feb

We explore gender differences in trust and trustworthiness between male and female individuals and groups consisting of two members of the same sex to understand if single sex groups behave differently from individuals of the same sex. We find some differences in the early rounds such as: (i) all-male (all-female) groups are more trusting than male (female) individuals and (ii) female individuals are most reciprocal compared to other entities. However, such early differences dissipate over time. We find that groups-whether single sex or mixed-behave very similar to each other and that there are little or no significant differences either in trust or trustworthiness between male and female groups. Our results have implications for the study of gender differences in economic transactions.

79. **INCLUDE IN ARTICLE REVIEW (coded)**
Cohesion and satisfaction as mediators of the team trust-Team effectiveness relationship: An interdependence theory perspective. [References].
DeOrtentiis, Philip S; Summers, James K; Ammeter, Anthony P; Douglas, Ceasar; Ferris, Gerald R.
The Career Development International. Vol.18(5), 2013, pp. 521-543.
[Journal; Peer Reviewed Journal]
Year of Publication
2013

Purpose: With extant research on the relationship between trust and effectiveness being inconclusive, the present study attempts to create a foundational investigation that examines the role of multiple mediators in the team trust-team effectiveness relationship. The authors identified the two emergent states of cohesion and satisfaction as intervening variables in the team trust-team effectiveness relationship, and tested this multi-mediation proposed model, within an interdependence theory perspective. Design/methodology/approach: MBA students at a large university in the southwestern USA were administered two waves of paper-based surveys, which were assigned to project groups. Individuals had been assigned to groups with the intent of maximizing diversity of age, gender, functional background, industry experience, and undergraduate major for each team (this is a goal of the MBA program, not the researchers). The first wave was administered one week after the groups had been formed and after all group members had met in their groups at least once. The second wave of surveys was administered at the end of the semester after the groups had been working together for three months and had completed their final projects. Findings: The results of the multiple mediation analysis found support for the hypotheses that cohesion and satisfaction serve as dual mediators of the trust-team effectiveness relationship. Originality/value: This study examined how trust operates through other variables to affect team performance. Two important variables that have been shown to be affected by trust are cohesion and satisfaction. Utilizing interdependence theory, the relationship of team trust and team performance was investigated through the intervening variables of cohesion and satisfaction, as both have been shown to possess properties that potentially represent different aspects of the interdependent relationship between team members. Therefore, this study examines how trust impacts team performance though the dual mediators of cohesion and satisfaction, in efforts to develop a more informed and theoretically grounded understanding of team performance and effectiveness processes.

80. **EXCLUDE (The study population and outcome of the study (the extent to which students valued the attachment to their class team and school) based upon “embeddedness” has limited transferability/applicability to CCCs - 2)**
Effects of school team embeddedness on team trust and potency. [References].
Chang, Jung-Nung; Cheng, Chia-Yi.
Quality & Quantity: International Journal of Methodology. Vol.47(4), 2013, pp. 1851-1868.
[Journal; Peer Reviewed Journal]
Year of Publication
2013
Publication Month/Season
Jun

This research surveyed a total of 323 local and foreign university students in Taiwan to examine the relationships of on- and off-the-team embeddedness with team behaviors by using the job embeddedness scale (JES) developed and validated by Lee et al. (Acad Manag J 47(5):711-722, 2004) and Mitchell et al. (Acad Manag J 44(6):1102-1121, 2001). The two composites of team embeddedness were measured with a total of 28 items, which asked students the extent to which they valued the attachment to their class team and school. For both local and foreign students, this research found that on-the-team embeddedness would affect team trust and team potency significantly while team trust would cause a positive direct effect on team potency. For foreign students, however, off-the-team embeddedness would not affect team potency significantly until team trust was introduced as a mediator. Moreover, significant differences were found in the relationships of nationality and years in university with off-the-team embeddedness. This study provides original evidence for the application of JES to school team relationships, and the link between school embeddedness and team effectiveness.

81. **EXCLUDE (This abstract was reviewed on the SCOPUS document)**
Team members' perceptions of online teamwork learning experiences and building teamwork trust: A qualitative study. [References].
Tseng, Hung Wei; Yeh, Hsin-Te.
Computers & Education. Vol.63 2013, pp. 1-9.
[Journal; Peer Reviewed Journal]
Year of Publication
2013
Publication Month/Season
Apr

82. **EXCLUDE (I think the concept of perceived group victimhood, which was evaluated in this study, has minimal overlap with CCCs - 2)**
Blinding trust: The effect of perceived group victimhood on intergroup trust. [References].
Rotella, Katie N; Richeson, Jennifer A; Chiao, Joan Y; Bean, Meghan G.
Personality and Social Psychology Bulletin. Vol.39(1), 2013, pp. 115-127.
[Journal; Peer Reviewed Journal]
Year of Publication
2013
Publication Month/Season
Jan

Four studies investigate how perceptions that one's social group has been victimized in society-that is, perceived group victimhood (PGV)-influence intergroup trust. Jewish and politically conservative participants played an economic trust game ostensibly with "partners" from their ingroup and/or a salient outgroup. Across studies, participants dispositionally or primed to be high in PGV revealed greater trust behavior with ingroup than outgroup partners. Control participants and those dispositionally low in PGV did not display such bias. Study 3 revealed, moreover, that high PGV enhanced ingroup trust even after an overt betrayal by an ingroup partner. Results were not explained by fluctuations in group identification, highlighting the novel, independent role of PGV in shaping an important aspect of intergroup relations-that is, trust. Implications of PGV for intergroup relations are discussed.

83. **EXCLUDE (Although interesting and applicable to my continued fight with the WR IRB, I don’t this the details of this study are applicable to CCCs - 2)**
Extending the olive branch: Enhancing communication and trust between research ethics committees and qualitative researchers. [References].
McMurphy, Suzanne; Lewis, Jacqueline; Boulos, Pierre.
Journal of Empirical Research on Human Research Ethics. Vol.8(4), 2013, pp. 28-36.
[Journal; Peer Reviewed Journal]
Year of Publication
2013
Publication Month/Season
Oct

Using data from a study of qualitative researchers' experiences with ethics review and our own lens as both researchers and REB/IRB members, we explore the ethics review process and provide recommendations for improvements. Our findings suggest that the review process would benefit from a strengthened trust relationship between REB/IRBs and qualitative researchers that would require a commitment from both sides. Regarding REBs/IRBs, increased transparency of the review process, consistent application of federal guidelines, and a more collaborative review approach may improve the trust of qualitative researchers. Regarding researchers, approaching ethics review as a form of academic peer review, similar to other types of assessments of scholarly products such as grants and publications, may promote the integration of ethics review as an intrinsic part of the research process. Recognizing that responsibility for ethical research is a shared goal of both researchers and REB/IRBs, improved collaboration and constructive interaction can assist in understanding each other's perspective and work toward the development of mutual trust and respect.

84. **INCLUDE IN ARTICLE REVIEW (This abstract was reviewed on the SCOPUS document)**
e-Leadership and trust management: Exploring the moderating effects of team virtuality. [References].
Jawadi, Nabila.
International Journal of Technology and Human Interaction (IJTHI). Vol.9(3), 2013, pp. 18-35.
[Journal; Peer Reviewed Journal]
Year of Publication
2013
Publication Month/Season
Jul-Sep

87. **INCLUDE IN ARTICLE REVIEW (waiting to code - Virtual Teams - 7)**
The effects of virtuality level on task-related collaborative behaviors: The mediating role of team trust. [References].
Penarroja, Vicente; Orengo, Virginia; Zornoza, Ana; Hernandez, Ana.
Computers in Human Behavior. Vol.29(3), 2013, pp. 967-974.
[Journal; Peer Reviewed Journal]
Year of Publication
2013
Publication Month/Season
May

This study aims to analyze the mediating role of team trust in the relationship between virtuality level and task-related collaborative behaviors. Three types of task-related collaborative behaviors were studied, namely team coordination, team cooperation, and team information exchange. Drawing upon theory and research on virtual teams and trust, we hypothesized that team trust partially mediated the effects of virtuality level on team coordination, team cooperation, and team information exchange. A laboratory experiment was carried out with 65 four-person teams randomly assigned to three communication media with different virtuality levels (face-to-face, video conference and computer-mediated communication). The results showed that team trust partially mediated the relationship between virtuality level and team coordination, and fully mediated this relationship with team cooperation and team information exchange.

90. **EXCLUDE (This study looks a transformational leadership and outcomes such as job satisfaction and team performance in academic work teams. I think there is limited applicability to CCCs focused more on group decision-making - 2)**
Transformational leadership, job satisfaction, and team performance: A multilevel mediation model of trust. [References].
Braun, Susanne; Peus, Claudia; Weisweiler, Silke; Frey, Dieter.
The Leadership Quarterly. Vol.24(1), 2013, pp. 270-283.
[Journal; Peer Reviewed Journal]
Year of Publication
2013
Publication Month/Season
Feb

In spite of calls for deliberate differentiation between individual and team levels of analysis, leadership research based on well-grounded theory referring to multiple levels is scarce. We seek to fill this gap by analyzing the relations between transformational leadership, trust in supervisor and team, job satisfaction, and team performance via multilevel analysis. Results are based on a sample of 360 employees from 39 academic teams. Transformational leadership was positively related to followers' job satisfaction at individual as well as team levels of analysis and to objective team performance. The relation between individual perceptions of supervisors' transformational leadership and job satisfaction was mediated by trust in the supervisor as well as trust in the team. Yet, trust in the team did not mediate the relationship between team perceptions of supervisors' transformational leadership and team performance. Implications for theory and research of leadership at multiple levels as well as for practice are discussed.

91. **EXCLUDE (This study of virtual teams seems less applicable to CCCs than some of the other studies we have included, especially given the fact that it likely looks at groups of students along with leader emergence as one of the main outcomes of this study - 7)**
Big Five personality factors and leader emergence in virtual teams: Relationships with team trustworthiness, member performance contributions, and team performance. [References].
Cogliser, Claudia C; Gardner, William L; Gavin, Mark B; Broberg, J. Christian.
Group & Organization Management. Vol.37(6), 2012, pp. 752-784.
[Journal; Peer Reviewed Journal]
Year of Publication
2012
Publication Month/Season
Dec

Using a sample of 243 undergraduate business students assigned to 71 virtual teams, this study explored the relationships between the Big Five personality factors, leader emergence, team trustworthiness, peer rated member performance contributions, and team performance. As predicted, agreeableness and conscientiousness were positively related to the task- and social-oriented dimensions of leader emergence, respectively. Contrary to expectations, emotional stability was not related to either dimension of leadership emergence. Evidence of the predicted relationships between emergent leadership and peer ratings of member contributions to team performance was obtained for task- but not social-oriented behaviors. At the team level, aggregated social-oriented leadership behaviors predicted aggregate perceptions of team trustworthiness. Only aggregated task-oriented emergent leadership behaviors predicted virtual team performance.

92. **INCLUDE IN ARTICLE REVIEW (This abstract was reviewed on the SCOPUS document)**
The role of trust climate in virtual teams. [References].
Brahm, Taiga; Kunze, Florian.
Journal of Managerial Psychology. Vol.27(6), 2012, pp. 595-614.
[Journal; Peer Reviewed Journal]
Year of Publication
2012

94. **EXCLUDE (This study of virtual teams seems less applicable to CCCs than some of the other studies we have included, especially given the fact that it likely looks at behavioral controls (which is not well defined in the abstract - 7)**
Trust is in the eye of the beholder: A vignette study of postevent behavioral controls' effects on individual trust in virtual teams. [References].
Dennis, Alan R; Robert, Lionel P Jr.; Curtis, Aaron M; Kowalczyk, Stacy T; Hasty, Bryan K.
Information Systems Research. Vol.23(2), 2012, pp. 546-558.
[Journal; Peer Reviewed Journal]
Year of Publication
2012
Publication Month/Season
Jun

Research in face-to-face teams shows conflicting results about the impact of behavioral controls on trust; some research shows that controls increase the salience of good behavior, which increases trust while other research shows that controls increase the salience of poor behavior that decreases trust. The only study in virtual teams, which examined poorly functioning teams, found that controls increased the salience of poor behavior, which decreased trust. We argue that in virtual teams behavioral controls amplify the salience of all behaviors (positive and negative) and that an individual's selective perception bias influences how these behaviors are interpreted. Thus the link from behavioral controls to trust is more complex than first thought. We conducted a 2 x 2 experiment, varying the use of behavioral controls (controls, no controls) and individual team member behaviors (reneging behaviors designed to reduce trust beliefs and fulfilling behaviors designed to increase trust beliefs). We found that behavioral controls did amplify the salience of all behaviors; however, contrary to what we expected, this actually weakened the impact of reneging and fulfilling behaviors on trust. We believe that completing a formal evaluation increased empathy and the awareness of context in which the behaviors occurred and thus mitigated extreme perceptions. We also found that behavioral controls increased the selective perception bias which induced participants to see the behaviors their disposition to trust expected rather than the behaviors that actually occurred.

95. **EXCLUDE (This study looks at the effects of positive group affective tone and team creativity, which I don’t feel is directly applicable or transferable to CCCs - 4)**
Positive group affective tone and team creativity: Negative group affective tone and team trust as boundary conditions. [References].
Tsai, Wei-Chi; Chi, Nai-Wen; Grandey, Alicia A; Fung, Sy-Chi.
Journal of Organizational Behavior. Vol.33(5), 2012, pp. 638-656.
[Journal; Peer Reviewed Journal]
Year of Publication
2012
Publication Month/Season
Jul

Drawing on multiple group-level theories, we explored boundary conditions of the relationship between positive group affective tone (PGAT) and team creativity. We collected data from members and leaders of 68 research and development teams and performed hierarchical linear modeling analyses to test our hypotheses. Consistent with the "group-centrism" perspective, we found that PGAT was beneficial for team creativity only when team trust was low; when trust was high, PGAT had a negative relationship with team creativity. In accord with the "dual-tuning" perspective, the positive effect of PGAT on creativity was present only when team trust was low but negative group affective tone was high. We discussed the theoretical and practical implications.

96. **INCLUDE IN ARTICLE REVIEW (coded)**
Trust development in swift starting action teams: A multilevel framework. [References].
Wildman, Jessica L; Shuffler, Marissa L; Lazzara, Elizabeth H; Fiore, Stephen M; Burke, C. Shawn; Salas, Eduardo; Garven, Sena.
Group & Organization Management. Vol.37(2), 2012, pp. 137-170.
[Journal; Peer Reviewed Journal]
Year of Publication
2012
Publication Month/Season
Apr

Swift starting action teams (STATs) are increasingly prevalent in organizations, and the development of trust is often a critical issue for their effectiveness. However, current theory and research do not provide a clear picture regarding how trust toward the team (i.e., the team as the target) is developed in these settings. The primary contribution of this article is to present a theoretical framework describing how individual-level trust toward one's team is developed in STAT contexts. This article integrates several existing trust theories into one comprehensive context-specific multilevel theory of how trust develops in STATs from cognitive, affective, behavioral, and contextual perspectives. This framework furthers our understanding of the unique antecedents of initial trust in STATs, how trust attitudes are adjusted over the short amount of time the team interacts, and how the team context influences this developmental process.

97. **INCLUDE IN ARTICLE REVIEW (EXCLUDE after full text review 16 July 2018. This article looks at the uncertainty management theory framework to predict reward fairness expectations of team members at the point of team formation and at team project completion. It also looks particularly at the use of reward allocation rules to ensure fairness. This does not seem to be applicable to CCCs)**
An inter- and intraindividual perspective of the substitutability of fairness rules for trust within teams. [References].
Merriman, Kimberly K; Maslyn, John; Farmer, Steven M.
Journal of Applied Social Psychology. Vol.42(4), 2012, pp. 850-873.
[Journal; Peer Reviewed Journal]
Year of Publication
2012
Publication Month/Season
Apr

The present study longitudinally assesses fairness allocation rule importance and equity allocation preference under conditions of evolving team trust. We predicted an interchangeable relationship between trust and allocation rules using an uncertainty management theory framework (Lind & Van den Bos, 2002; Van den Bos & Lind, 2002). From an interindividual perspective, lower initial trust toward team members predicted a higher degree of importance for the use of preferred allocation rules and greater use of the equity heuristic. An intraindividual change in trust predicted an inverse change in use of the equity heuristic, but not the expected change in allocation rule importance. Implications of these results for future research and practice are discussed.

98. **INCLUDE IN ARTICLE REVIEW (This abstract was reviewed on the SCOPUS document and linked to citation 100 below)**
" Leader fairness and employees' trust in coworkers: The moderating role of leader group prototypicality": Correction to Seppala, Lipponen, and Pirttila-Backman (2012).
Seppala, Tuija; Lipponen, Jukka; Pirttila-Backman, Anna-Maija.
Group Dynamics: Theory, Research, and Practice. Vol.16(1), 2012, pp. 49.
[Journal; Peer Reviewed Journal]
Year of Publication
2012
Publication Month/Season
Mar

99. **EXCLUDE (This abstract was reviewed on the SCOPUS document)**
Two experimental tests of trust in in-group strangers: The moderating role of common knowledge of group membership. [References].
Platow, Michael J; Foddy, Margaret; Yamagishi, Toshio; Lim, Li; Chow, Aurore.
European Journal of Social Psychology. Vol.42(1), 2012, pp. 30-35.
[Journal; Peer Reviewed Journal]
Year of Publication
2012
Publication Month/Season
Feb

100. **INCLUDE IN ARTICLE REVIEW (This abstract was reviewed on the SCOPUS document and linked to citation 98 above)**
Leader fairness and employees' trust in coworkers: The moderating role of leader group prototypicality. [References].
Seppala, Tuija; Lipponen, Jukka; Pirttila-Backman, Anna-Maija.
Group Dynamics: Theory, Research, and Practice. Vol.16(1), 2012, pp. 35-49.
[Journal; Peer Reviewed Journal]
Year of Publication
2012
Publication Month/Season
Mar

101. **INCLUDE IN ARTICLE REVIEW (coded)**
Beyond shared perceptions of trust and monitoring in teams: Implications of asymmetry and dissensus. [References].
De Jong, Bart A; Dirks, Kurt T.
Journal of Applied Psychology. Vol.97(2), 2012, pp. 391-406.
[Journal; Peer Reviewed Journal]
Year of Publication
2012
Publication Month/Season
Mar

Past research has implicitly assumed that only mean levels of trust and monitoring in teams are critical for explaining their interrelations and their relationships with team performance. In this article, the authors argue that it is equally important to consider the dispersion in trust and monitoring that exists within teams. The authors introduce "trust asymmetry" and "monitoring dissensus" as critical dispersion properties of trust and monitoring and hypothesize that these moderate the relationships between mean monitoring, mean trust, and team performance. Data from a cross-lagged panel study and a partially lagged study support the hypotheses. The first study also offered support for an integrative model that includes mean and dispersion levels of both trust and monitoring. Overall, the studies provide a comprehensive and clear picture of how trust and monitoring emerge and function at the team level via mean and dispersion.

103. **EXCLUDE (Outgroup liking and relationships between groups really isn’t applicable to CCCs - 4)**
When increased group identification leads to outgroup liking and cooperation: The role of trust. [References].
Montoya, R. Matthew; Pittinsky, Todd L.
The Journal of Social Psychology. Vol.151(6), 2011, pp. 784-806.
[Journal; Peer Reviewed Journal]
Year of Publication
2011
Publication Month/Season
Nov

Two studies explored the influence of group identification and the functional relations between groups on outgroup liking. In a laboratory study, Study 1 (N = 112) found that outgroup liking was highest when group identification was high and relations between groups were cooperative, but outgroup liking was lowest when group identification was high and relations were competitive. In a field replication of Study 1, Study 2 (N = 181) similarly found more liking with high group identification and cooperative relations between groups. Additional analyses revealed that the Identification x Relations interactions found in Studies 1 and 2 were mediated by outgroup trust. We discuss how trust is an important factor for predicting outgroup bias for both high and low group identification.

104. **EXCLUDE (The population studied and the outcome of social capital is really not applicable to CCCs - 2)**
Group-based trust, trustworthiness and voluntary cooperation: Evidence from experimental and survey data in china. [References].
Qin, Xiangdong; Shen, Junyi; Meng, Xindan.
The Journal of Socio-Economics. Vol.40(4), 2011, pp. 356-363.
[Journal; Peer Reviewed Journal]
Year of Publication
2011
Publication Month/Season
Aug

Trust, trustworthiness and cooperation are crucial in achieving social goals, and are thus essential components of social capital. This paper reports the results of a series of lab and artefactual field experiments carried out in Shanghai to evaluate some of the key indicators of social capital in China. The groups selected for the study are middle school and undergraduate university students and community residents. The experiments comprise two public goods games, a gambling game and a trust game. The overall level of trust is negatively related to age, although trusting behavior is also affected by other factors, such as risk-taking.

106. **INCLUDE IN ARTICLE REVIEW (coded)**
Measuring trust in teams: Development and validation of a multifaceted measure of formative and reflective indicators of team trust. [References].
Costa, Ana Cristina; Anderson, Neil.
European Journal of Work and Organizational Psychology. Vol.20(1), 2011, pp. 119-154.
[Journal; Peer Reviewed Journal]
Year of Publication
2011
Publication Month/Season
Feb

This article describes the development and validation of a multidimensional instrument designed to measure trust within teams. Trust is conceptualized as a latent variable resulting from distinct but related (formative) indicators, i.e., propensity to trust and perceived trustworthiness, which lead to (reflective) indicators, i.e., behaviours cooperation and monitoring between team members. The instrument was tested in two different samples. The first sample included 98 hospital professionals (14 teams) and provided data for the exploratory factor analysis of this instrument (Study 1). The second sample included responses from 112 teams (395 individuals) from three social-care institutions and was used to apply confirmatory factor analysis (Study 2). Data attesting to the underlying factor structure, internal homogeneity, construct validity, and consensual power of agreement within teams and discriminant power across teams of the instrument are presented. The final 21-item, four-factor version of this measure demonstrates good psychometric properties, with acceptable levels of reliability and validity. We conclude that the scales form a parsimonious, valid, and efficient instrument to assess trust in teams. Potential applications of this measure in research and practice are described and the implications of these findings for future research are discussed.

107. **EXCLUDE (This article focuses on different leadership styles of teams in the financial industry. Although leadership is an important component of our model/study, I don’t this study applies directly to CCCs - 2)**
Cognition-based and affect-based trust as mediators of leader behavior influences on team performance. [References].
Schaubroeck, John; Lam, Simon S. K; Peng, Ann Chunyan.
Journal of Applied Psychology. Vol.96(4), 2011, pp. 863-871.
[Journal; Peer Reviewed Journal]
Year of Publication
2011
Publication Month/Season
Jul

We develop a model in which cognitive and affective trust in the leader mediate the relationship between leader behavior and team psychological states that, in turn, drive team performance. The model is tested on a sample of 191 financial services teams in Hong Kong and the U.S. Servant leadership influenced team performance through affect-based trust and team psychological safety. Transformational leadership influenced team performance indirectly through cognition-based trust. Cognition-based trust directly influenced team potency and indirectly (through affect-based trust) influenced team psychological safety. The effects of leader behavior on team performance were fully mediated through the trust in leader variables and the team psychological states. Servant leadership explained an additional 10% of the variance in team performance beyond the effect of transformational leadership. We discuss implications of these results for research on the relationship between leader behavior and team performance, and for efforts to enhance leader development by combining knowledge from different leadership theories.

108. **INCLUDE IN ARTICLE REVIEW (This abstract was reviewed on the SCOPUS document)**
Authentically leading groups: The mediating role of collective psychological capital and trust. [References].
Walumbwa, Fred O; Luthans, Fred; Avey, James B; Oke, Adegoke.
Journal of Organizational Behavior. Vol.32(1), 2011, pp. 4-24.
[Journal; Peer Reviewed Journal]
Year of Publication
2011
Publication Month/Season
Jan

109. **EXCLUDE (This abstract was reviewed on the SCOPUS document)**
Trust, performance, and the communication process in ad hoc decision-making virtual teams. [References].
Altschuller, Shoshana; Benbunan-Fich, Raquel.
Journal of Computer-Mediated Communication. Vol.16(1), 2010, pp. 27-47.
[Journal; Peer Reviewed Journal]
Year of Publication
2010
Publication Month/Season
Oct

110. **INCLUDE IN ARTICLE REVIEW (This article was reviewed on the SCOPUS document)**
The impact of structural and contextual factors on trust formation in product development teams. [References].
Dayan, Mumin; Di Benedetto, C. Anthony.
Industrial Marketing Management. Vol.39(4), 2010, pp. 691-703.
[Journal; Peer Reviewed Journal]
Year of Publication
2010
Publication Month/Season
May

111. **INCLUDE IN ARTICLE REVIEW (waiting to code - Virtual Teams - 7)**
Fostering trust in virtual project teams: Towards a design framework grounded in a TrustWorthiness ANtecedents (TWAN) schema. [References].
Rusman, Ellen; van Bruggen, Jan; Sloep, Peter; Koper, Rob.
International Journal of Human-Computer Studies. Vol.68(11), 2010, pp. 834-850.
[Journal; Peer Reviewed Journal]
Year of Publication
2010
Publication Month/Season
Nov

Several collaboration problems in virtual project teams that work in knowledge-intensive contexts can be attributed to a hampered process of interpersonal trust formation. Solutions to trust formation problems need to be based on an understanding of how interpersonal trust forms in face-to-face project teams as well as on insight into how this process differs in virtual teams. Synthesizing literature from various disciplines, we propose a model for the formation of interpersonal trust between project team members. Taking this model as a starting point, we analyse how virtual settings may alter or even obstruct the process of trust formation. One method to improve the formation of interpersonal trust in virtual settings is to facilitate the assessment of trustworthiness. This can be done by making information available about individual virtual project team members. Previous research in virtual project teams focussed principally on the medium by which information is spread, for example, by phone, mail, or videoconferencing. Most researchers failed to take the specific content of the information into account, although there is general agreement that personal, non-task-related information is important to foster trust. For this, we propose to use the antecedents of trustworthiness, which until now have mainly been used as a framework to measure trust, as a design framework instead. This framework of antecedents can also be used to determine which type of information is relevant to assess each other's trustworthiness. We review existing literature on the antecedents of trustworthiness and extend the well-accepted antecedents of 'ability', 'benevolence' and 'integrity' with several other antecedents, such as 'communality' and 'accountability'. Together, these form the TrustWorthiness ANtecedents (TWAN) schema. We describe how these antecedents can be used to determine which information is relevant for team members assessing others' trustworthiness. In future research we will first verify this extended cognitive schema of trustworthiness (TWAN) empirically and then apply it to the design of artefacts or guidelines, such as a personal identity profile to support the assessment of trustworthiness in virtual project teams.

112. **INCLUDE IN ARTICLE REVIEW (coded)**
Team identification, trust and conflict: A mediation model. [References].
Han, Guohong; Harms, P. D.
International Journal of Conflict Management. Vol.21(1), 2010, pp. 20-43.
[Journal; Peer Reviewed Journal]
Year of Publication
2010

Purpose: The current study aims to address the relationship between trust, team identification, and team conflict. Specifically, it aims to examine whether trust in peers mediates the relationship between team identification and team conflict. Design/methodology/approach: This is an empirical paper based on two field studies. In Study 1, 241 employees in a US Fortune 500 company distributed in various and mostly R&D teams were surveyed. In Study 2, 205 employees in a health care organization in the Midwest were surveyed. Findings: Team identification was related to lower levels of both task conflict and relationship conflict. This relationship, however, is mediated by the employees' trust in their peers. Research limitations/implications: This finding addresses concerns about the mechanisms by which employee attitudes contribute to work behaviors. Practical implications: This study highlights the importance of cultivating team members' sense of "we" rather than a sense of "I" in the team context, reinforced the crucial role of trust in organizational context. Further, by shedding light on the process by which teams come into conflict, our results suggest a means by which managers and organizations can work towards creating optimal levels of conflict in their work teams. Originality/value: As far as it is known, this is the first field study that has examined the mediating role of trust between team identity and team conflict.

113. **EXCLUDE (This article was reviewed on the SCOPUS document)**
Testing a moderated mediational model of workgroup incivility: The roles of organizational trust and group regard. [References].
Miner-Rubino, Kathi; Reed, Whitney D.
Journal of Applied Social Psychology. Vol.40(12), 2010, pp. 3148-3168.
[Journal; Peer Reviewed Journal]
Year of Publication
2010
Publication Month/Season
Dec

114. **EXCLUDE (This article looks at professional elite athletes and the teams to which they belong. This likely has minimal applicability to CCCs - 2)**
The differential effect of team members' trust on team performance: The mediation role of team cohesion. [References].
Mach, Merce; Dolan, Simon; Tzafrir, Shay.
Journal of Occupational and Organizational Psychology. Vol.83(3), 2010, pp. 771-794.
[Journal; Peer Reviewed Journal]
Year of Publication
2010
Publication Month/Season
Sep

This study examines team performance as affected by various trusting relationships: trust between team members and the team's trust in their direct manager and in top management. Data for the study were collected from a survey of 690 professional elite athletes (belonging to 59 different sports clubs) playing in the regular, top professional Spanish leagues. The model was tested at the team level. Findings reveal that team member trust with respect to the different foci has both a direct and indirect effect on team performance, and that team player trust and cohesion play a mediating role. This study illustrates the dynamic relationship within teams, and, as such, trust among teammates mediates the relationship between trust in the coach as well as team cohesion in determining team performance. The implications for managing teams in other contexts are also evaluated.

115. **EXCLUDE (The outcome of creativity in the context of a study looking at undergraduate students is not very applicable to CCCs - 2)**
Antecedents of team creativity: An examination of team emotional intelligence, team trust and collaborative culture. [References].
Barczak, Gloria; Lassk, Felicia; Mulki, Jay.
Creativity and Innovation Management. Vol.19(4), 2010, pp. 332-345.
[Journal; Peer Reviewed Journal]
Year of Publication
2010
Publication Month/Season
Dec

Teams represent a dominant approach to getting work done in a business environment. Creativity enables teams to solve problems and leverage opportunities through the integration of divergent thoughts and perspectives. Prior research indicates that a collaborative culture, which affects how team members interact and work together, is a critical antecedent of team creativity. This study explores other antecedents of team creativity, namely, team emotional intelligence and team trust, and investigates the relationships among these precursors to creative effort. Using a survey of 82 student teams at a large university in the northeast United States, our findings suggest that team emotional intelligence promotes team trust. Trust, in turn, fosters a collaborative culture which enhances the creativity of the team. Cognitive trust also moderates the relationship between collaborative culture and team creativity. Implications of these results for managers and academics are discussed.

116. **EXCLUDE (This article focuses on engineering project teams in the automotive industry along with leadership. I don’t think this study is as applicable as some of the other we have included - 2)**
Leadership and trust: Their effect on knowledge sharing and team performance. [References].
Lee, Pauline; Gillespie, Nicole; Mann, Leon; Wearing, Alexander.
Management Learning. Vol.41(4), 2010, pp. 473-491.
[Journal; Peer Reviewed Journal]
Year of Publication
2010
Publication Month/Season
Sep

Team leaders who facilitate knowledge sharing and engender trust contribute to team effectiveness. While the separate effects of leadership, trust and knowledge sharing on team performance are well documented, few scholars have investigated the specific links between these factors. This study examines the relationship between the leader as the knowledge builder, trust in the leader and in the team, knowledge sharing and team performance. Surveys were collected from 34 engineering project teams (n=166 team members, 30 team leaders) and 18 managers in a large automotive organization. The results indicate that by building the team's expertise, leaders enhance team members' willingness to rely on and disclose information in the team, which in turn increases team knowledge sharing. Team knowledge sharing significantly predicted leaders' and managers' ratings of team performance. The theoretical and practical implications of the findings are discussed.

117. **EXCLUDE (The population studied, in this case tax consulting teams, are not likely to have a significant amount of overlap with CCCs - 2)**
How does trust affect the performance of ongoing teams? The mediating role of reflexivity, monitoring, and effort. [References].
de Jong, Bart; Elfring, Tom.
Academy of Management Journal. Vol.53(3), 2010, pp. 535-549.
[Journal; Peer Reviewed Journal]
Year of Publication
2010
Publication Month/Season
Jun

In this study, we investigate how trust affects the performance of ongoing teams. We propose a multiple mediator model in which different team processes act as mediating mechanisms that transmit the positive effects of trust to team performance. Drawing on a data set of ongoing tax consulting teams, we found support for the mediated effects of trust via team monitoring and team effort. Our results did not support the mediating role of "team reflexivity." These findings contribute to understanding how trust operates within ongoing teams in a way that is distinct from what is known from studies of short-term teams.

118. **INCLUDE IN ARTICLE REVIEW (This abstract was reviewed on the PUBMED document)**
Voice in political decision-making: The effect of group voice on perceived trustworthiness of decision makers and subsequent acceptance of decisions. [References].
Terwel, Bart W; Harinck, Fieke; Ellemers, Naomi; Daamen, Dancker D. L.
Journal of Experimental Psychology: Applied. Vol.16(2), 2010, pp. 173-186.
[Journal; Peer Reviewed Journal]
Year of Publication
2010
Publication Month/Season
Jun

119. **INCLUDE IN ARTICLE REVIEW (coded)**
The role of group member affect in the relationship between trust and cooperation. [References].
Tanghe, Jacqueline; Wisse, Barbara; van der Flier, Henk.
British Journal of Management. Vol.21(2), 2010, pp. 359-374.
[Journal; Peer Reviewed Journal]
Year of Publication
2010
Publication Month/Season
Jun

It is widely acknowledged that trust greatly affects work group functioning. Whereas trust may facilitate cooperation, distrust may impede it. Insight into when distrusters may be prompted to cooperate may therefore be of importance. Empirical studies point to several moderators of the effect of trust on cooperation. Unfortunately, these studies largely ignored the potential role of group member affect. Our study shows that group members' affective displays (particularly the activation level of the displays) have a substantial impact on the relationship between trust and cooperation. First, a scenario experiment (n = 80) revealed that low trusting individuals were more willing to cooperate when confronted with group members who display high (versus low) activation affective states, whereas for more high trusting individuals cooperation was not contingent on other group members' affective displays. Second, a laboratory experiment (n = 78), employing a social dilemma paradigm, replicated these findings and indicated that this effect is explained by the extent to which others are expected to cooperate. The discussion focuses on theoretical implications and managerial ramifications. Our study testifies to the significant role that affect may play in keeping up cooperation in organizations and work groups when trust is withering.

120. **EXCLUDE (I don’t think the definition of trust in this setting is consistent with our study. This article focuses more on implementing EBM guidelines for healthcare groups to improve sepsis outcomes - 1)**
Surviving sepsis: A trust-wide approach. A multi-disciplinary team approach to implementing evidence-based guidelines. [References].
Gerber, Karin.
Nursing in Critical Care. Vol.15(3), 2010, pp. 141-151.
[Journal; Peer Reviewed Journal]
Year of Publication
2010
Publication Month/Season
May-Jun

Aim: To share an experience of examining the true extent of the number of patients with severe sepsis being admitted, and the overall compliance with existing treatment guidelines in a district general hospital (DGH). Background: Because of its aggressive, multi-factorial nature, sepsis is a rapid killer. Mortality associated with severe sepsis remains unacceptably high: 30-50%. When shock is present, mortality is reported to be even higher: 50-60%. The rapid diagnosis and management of sepsis is vital to successful treatment. The International Surviving Sepsis Campaign (SSC) was developed to help meet the challenges of sepsis and to improve its management, diagnosis and treatment. The overall aim is to reduce mortality from sepsis by 25% by 2009. Data sources and methods: Data on the number of patients admitted with severe sepsis to the DGH were previously unknown. The aim of the baseline audits was to determine the true extent of the problem and baseline mortality rates, resulting in an action plan to provide evidence-based care to patients with sepsis regardless of where in the hospital they were located. Results: It was found that 11% of the patients audited presented with signs of severe sepsis and demonstrated elements of poor compliance with some elements of existing treatment guidelines as set out by the resuscitation component of the Surviving Sepsis Care Bundle. Conclusion: As an international campaign introduced predominantly within critical care, within this DGH the SSC teams' innovative approach has resulted in: 1. Better educated staff; 2. Objectives agreed within multi-disciplinary teams; 3. The appropriate assessment of resources; 4. Standardization of practice in terms of patients presenting with severe sepsis.

121. **INCLUDE IN ARTICLE REVIEW (This abstract was reviewed on the SCOPUS document)**
Effects of culture, social presence, and group composition on trust in technology-supported decision-making groups. [References].
Lowry, Paul Benjamin; Zhang, Dongsong; Zhou, Lina; Fu, Xiaolan.
Information Systems Journal. Vol.20(3), 2010, pp. 297-315.
[Journal; Peer Reviewed Journal]
Year of Publication
2010
Publication Month/Season
May

122. **INCLUDE IN ARTICLE REVIEW (coded)**
Does conflict shatter trust or does trust obliterate conflict? Revisiting the relationships between team diversity, conflict, and trust. [References].
Curseu, Petru Lucian; Schruijer, Sandra G. L.
Group Dynamics: Theory, Research, and Practice. Vol.14(1), 2010, pp. 66-79.
[Journal; Peer Reviewed Journal]
Year of Publication
2010
Publication Month/Season
Mar

This article explores the interplay between trust and conflict as antecedents of team effectiveness. In the first cross-sectional study, two alternative path models are tested in a sample of 174 teams (897 participants) with the emergent states of task conflict, relationship conflict, and trust acting as mediators between team demographic diversity (gender and nationality) on the one hand and perceived team effectiveness on the other. In one model trust is considered as an antecedent for the two types of conflict, while in the other the two types of conflict precede the emergence of trust. Although the fit indices for the model in which trust is considered the antecedent of conflict were slightly better, both models fitted the data well. The interdependence of trust and conflict was further explored in a second longitudinal study (49 teams), and the results showed that trust emerging in the initial team interaction phases is a good predictor for the emergence of both task and relationship conflict in further stages of team development.

123. **EXCLUDE (This abstract was reviewed on the SCOPUS document)**
An exploratory study of trust dynamics in work-oriented virtual teams. [References].
Kuo, Feng-yang; Yu, Chia-ping.
Journal of Computer-Mediated Communication. Vol.14(4), 2009, pp. 823-854.
[Journal; Peer Reviewed Journal]
Year of Publication
2009
Publication Month/Season
Jul

125. **EXCLUDE (We have included some other studies that look at this concept, and I vote we exclude this one given the population studied (Taiwanese corporate teams) - 2)**
Shared work values and team member effectiveness: The mediation of trustfulness and trustworthiness. [References].
Chou, Li-Fang; Wang, An-Chih; Wang, Ting-Yu; Huang, Min-Ping; Cheng, Bor-Shiuan.
Human Relations. Vol.61(12), 2008, pp. 1713-1742.
[Journal; Peer Reviewed Journal]
Year of Publication
2008
Publication Month/Season
Dec

Using a sample of 411 members and their respective leaders from 72 Taiwanese corporate teams,we conducted a cross-level study and found that 1) teammates' shared work values were positively related to team member performance and satisfaction with cooperation; 2) trustworthiness, or how a member was trusted by his or her teammates, mediated the relationship between shared work values and team member performance; and 3) trustfulness, or how a member trusted his or her teammates, mediated the relationship between shared work values and satisfaction with cooperation. Results provided support for the shared mental model theory and the directional nature of interpersonal trust.

126. **EXCLUDE (This study looks at a multicultural workforce in a pharmaceutical company, and trust in this setting. I don’t think this is directly applicable or transferable to CCCs - 4)**
The role of trust in core team employees: A three-nation study. [References].
More, Keren V; Tzafrir, Shay S.
Cross Cultural Management. Vol.16(4), 2009, pp. 410-433.
[Journal; Peer Reviewed Journal]
Year of Publication
2009

Purpose: The paper aims to fill a substantive gap in the trust literature by analyzing the mediating role of trust across different national groups. It presents a theoretical advantage, from the human resource management perspective, is that the research model was examined within the context of a global pharmaceutical firm characterized by a multicultural workforce. This allowed for controlling organizational culture. Design/methodology/approach: The research hypotheses were tested using data regarding only core team employees. Survey data obtained from employees of a pharmaceutical company in Israel, the UK, and Hungary. After the teams in each of the organization's three sites were mapped out, 205 employees working on 62 teams responded to the questionnaire resulting in a good quality of 61 percent response rate. Findings: The results of zero-order co-relational and confirmatory factor analysis revealed the two trust foci to be interrelated, but distinct, constructs. This finding suggests that employees can develop trust in specific individuals, such as superiors, and generalized representatives, such as the organization. The mixed models analysis suggest that there are significant differences between the Israeli, UK, and Hungarian employees for three of the research variables: trust in the organization, turnover intentions, and organizational citizenship behavior (OCB). Originality/value: The paper is one of the first cross-national empirical studies of its kind to demonstrate the role of trust as a mediator between organizational justice and employee work attitudes and behaviors. In addition, it is one of the first cross-national studies on trust that controlled both industrial characteristics and organizational culture.

129. **EXCLUDE (This study of virtual teams seems less applicable to CCCs than some of the other studies we have included. It is also close to 10 years old - 7)**
An examination of the roles of trust and functional diversity on virtual team performance ratings. [References].
Peters, Linda; Karren, Ronald J.
Group & Organization Management. Vol.34(4), 2009, pp. 479-504.
[Journal; Peer Reviewed Journal]
Year of Publication
2009
Publication Month/Season
Aug

This study investigates the relationship among trust, functional diversity, and team performance in a virtual environment. The authors conducted research on more than 200 team members representing 33 virtual teams and found that both trust and functional diversity had a direct impact on team performance using team member ratings; however, neither had a direct impact on team performance using external manager ratings. Instead, trust was found to moderate the functional diversity to performance relationship using the managers' ratings. Differences in the development of trust between virtual teams and face-to-face teams are also discussed, as are the implications that the results of this study may have on organizations, managers, and team members.

130. **INCLUDE IN ARTICLE REVIEW (coded)**
Team negotiation and perceptions of trustworthiness: The whole versus the sum of the parts. [References].
Naquin, Charles E; Kurtzberg, Terri R.
Group Dynamics: Theory, Research, and Practice. Vol.13(2), 2009, pp. 133-150.
[Journal; Peer Reviewed Journal]
Year of Publication
2009
Publication Month/Season
Jun

In a team negotiation context, the authors empirically explored how judgments of team-level trust are derived from individual-level trust. Basing their argument on both the negativity bias and the discontinuity effect, the authors posit that people will focus most on the least trustworthy individual member of a team when making judgments about collective team-level trust. Findings from two studies demonstrate that perceptions of team trust are indeed lower than the average ratings of individual trust and are statistically equivalent to the least trusted member. In addition, compared with average individual trust levels, perceptions of collective team trust were found to be more predictive of (a) impasse rates in distributive negotiations and (b) the level of joint gain in integrative negotiations.

131. **EXCLUDE (This abstract was reviewed on the SCOPUS document)**
The roles of group importance and anxiety in predicting depersonalized ingroup trust. [References].
Kenworthy, Jared B; Jones, Jennifer.
Group Processes & Intergroup Relations. Vol.12(2), 2009, pp. 227-239.
[Journal; Peer Reviewed Journal]
Year of Publication
2009
Publication Month/Season
Mar

132. **EXCLUDE (This abstract was reviewed on the SCOPUS document)**
Organizational context and face-to-face interaction: Influences on the development of trust and collaborative behaviors in computer-mediated groups. [References].
Hill, N. Sharon; Bartol, Kathryn M; Tesluk, Paul E; Langa, Gosia A.
Organizational Behavior and Human Decision Processes. Vol.108(2), 2009, pp. 187-201.
[Journal; Peer Reviewed Journal]
Year of Publication
2009
Publication Month/Season
Mar

133. **EXCLUDE (The population studied in this article likely has minimal transferability and applicability to CCCs - 2)**
Authentic leadership and positive psychological capital: The mediating role of trust at the group level of analysis. [References].
Clapp-Smith, Rachel; Vogelgesang, Gretchen R; Avey, James B.
Journal of Leadership & Organizational Studies. Vol.15(3), 2009, pp. 227-240.
[Journal; Peer Reviewed Journal]
Year of Publication
2009
Publication Month/Season
Feb

This study investigates the relationship between authentic leadership, trust, positive psychological capital (PsyCap), and performance at the group level of analysis. Data were collected from a small Midwestern chain of retail clothing stores, a context in which the needs for both authentic leadership and a positive sales staff are integral to the firm's performance. Constructs were aggregated to the store (group) level to test relationships between perceptions of authentic leadership, trust in management, positive psychological capital, and performance. Trust in management was found to mediate the relationship between PsyCap and performance and to partially mediate the relationship between authentic leadership and performance. Future discussions and implications are discussed.

134. **EXCLUDE (This abstract was reviewed on the SCOPUS document)**
Intergroup trust and reciprocity in strategic interactions: Effects of group decision-making mechanisms. [References].
Song, Fei.
Organizational Behavior and Human Decision Processes. Vol.108(1), 2009, pp. 164-173.
[Journal; Peer Reviewed Journal]
Year of Publication
2009
Publication Month/Season
Jan

135. **EXCLUDE (I’m not sure this article is in English and based on the abstract, this sounds like more of an opinion piece. If it is a research study, the structure of the research is not well articulated - 7)**
Trust and team coordination in critical situations. [References].
Pitariu, Adrian H.
Psihologia Resurselor Umane Revista Asociatiei de Psihologie Indusstriala si Organizationala. Vol.6(1), 2008, pp. 19-24.
[Journal; Peer Reviewed Journal]
Year of Publication
2008

In this paper I explore the process of team coordination. I propose a model by which team coordination emerges as a function of team mental models and team trust. Furthermore, I introduce a hierarchical approach to team mental models, and propose a framework that provides a better understanding of team processes and opens new avenues of research in the area of team cognition. I conclude with implications for future research and practice.

136. **EXCLUDE (The topic of cognitive and affective trust has been looked at in other articles we are including. The population of this study (student project teams over the course of a semester) limit the transferability and applicability to CCCs - 2)**
Development of cognitive and affective trust in teams: A longitudinal study. [References].
Webber, Sheila Simsarian.
Small Group Research. Vol.39(6), 2008, pp. 746-769.
[Journal; Peer Reviewed Journal]
Year of Publication
2008
Publication Month/Season
Dec

The present research examines the development of two dimensions of trust, cognitive and affective, in student project teams over the course of a semester. Empirical examination of the evolution of multidimensional trust and its unique antecedents was explored. The results show that early trust emerges as a one-dimensional factor early in the life span of a team; cognitive and affective trust emerge as separate components over time; unique and distinct predictors positively and negatively affect early trust, cognitive trust, and affective trust; and affective trust has a stronger positive relationship with team performance than cognitive trust.

137. **INCLUDE IN ARTICLE REVIEW (This abstract was reviewed on the SCOPUS document)**
Exploring the effects of trust, task interdependence and virtualness on knowledge sharing in teams. [References].
Staples, D. Sandy; Webster, Jane.
Information Systems Journal. Vol.18(6), 2008, pp. 617-640.
[Journal; Peer Reviewed Journal]
Year of Publication
2008
Publication Month/Season
Nov

139. **EXCLUDE (The idea of dyadic and group-level discovery in a leadership setting does not seem very applicable to CCCs - 2)**
Increasing trust, psychological safety, and team performance through dyadic leadership discovery. [References].
Roussin, Christopher J.
Small Group Research. Vol.39(2), 2008, pp. 224-248.
[Journal; Peer Reviewed Journal]
Year of Publication
2008
Publication Month/Season
Apr

In this article, the author uses a case-based argument to explore the idea that team members have grounded rationality, which may be actively learned by team leaders through a process of leadership discovery. The analysis reveals evidence that leaders using dyadic discovery methods learn more and more effectively customize leadership behaviors to increase trust, psychological safety, and team performance. A comparison of dyadic and group-level discovery cases lends support to the theory that dyadic-level discovery is psychologically safer and more consistently effective than is group-level discovery in most work team settings. This is an important finding, as many "action" and "organization development" methods presently rely on group-level interventions as a means of uncovering individual-level rationality.

140. **EXCLUDE (This abstract was reviewed on the SCOPUS document)**
Trust and reciprocity behavior and behavioral forecasts: Individuals versus group-representatives. [References].
Song, Fei.
Games and Economic Behavior. Vol.62(2), 2008, pp. 675-696.
[Journal; Peer Reviewed Journal]
Year of Publication
2008
Publication Month/Season
Mar

141. **EXCLUDE (This study of virtual teams seems less applicable to CCCs than some of the other studies we have included, especially given the fact that it looks at project teams and in-group/out-group theories of subgroups - 7)**

Comparing traditional and virtual group forms: Identity, communication and trust in naturally occurring project teams. [References].
Webster, J; Wong, W. K. P.
The International Journal of Human Resource Management. Vol.19(1), 2008, pp. 41-62.
[Journal; Peer Reviewed Journal]
Year of Publication
2008
Publication Month/Season
Jan

This study compares three types of project teams in a global high-tech organization: traditional (co-located), virtual (completely distributed), and 'semi-virtual' or hybrid (containing both local and remote members). We use in-group/out-group theories of subgroups to help explain the findings. Specifically, local members of semi-virtual teams report much more positive perceptions of their local than their remote members, while traditional and virtual team members appear similar. We conclude by drawing implications for practice, such as the avoidance of semi-virtual teams whenever possible and the development of strong team identities.

142. **INCLUDE IN ARTICLE REVIEW (EXCLUDE after full text review on 10 Aug 2018. In this setting, heedful interrelating is referenced to understand group performance in situations that require nearly continuous operational reliability, such as flight decks. The authors contend, however, that heedful interrelating is also a relevant concept in organizations where task interdependence is high and task programmability is low. While this may apply to CCCs, the article is very challenging to follow, and I have a hard time relating it to our research study)**
Heed, a missing link between trust, monitoring and performance in knowledge intensive teams. [References].
Bijlsma-Frankema, K; de Jong, Bart; van de Bunt, Gerhard.
The International Journal of Human Resource Management. Vol.19(1), 2008, pp. 19-40.
[Journal; Peer Reviewed Journal]
Year of Publication
2008
Publication Month/Season
Jan

This longitudinal study aimed to explain performance differences of knowledge intensive project teams. Team level data gathered at three measurement moments were used. Antecedents of performance studied were: trust in team members, trust in supervisors, monitoring by team members and monitoring by supervisors. Heedful interrelating, a concept developed by Weick and Roberts (1993) was expected to mediate between trust in team members, monitoring by team members and team performance. Correlation analysis and structural equation modelling were employed to analyse the data. The results show that heedful interrelating of team members, built on a combination of trust and monitoring by team members and trust in supervisors is an important factor in promoting team performance.

143. **EXCLUDE (This abstract was reviewed on the SCOPUS document)**
Trust between individuals and groups: Groups are less trusting than individuals but just as trustworthy. [References].
Kugler, Tamar; Bornstein, Gary; Kocher, Martin G; Sutter, Matthias.
Journal of Economic Psychology. Vol.28(6), 2007, pp. 646-657.
[Journal; Peer Reviewed Journal]
Year of Publication
2007
Publication Month/Season
Dec

145. **INCLUDE IN ARTICLE REVIEW (This abstract was reviewed on the SCOPUS document)**
The relationship between conflict and decision outcomes: Moderating effects of cognitive- and affect-based trust in strategic decision-making teams. [References].
Parayitam, Satyanarayana; Dooley, Robert S.
International Journal of Conflict Management. Vol.18(1), 2007, pp. 42-73.
[Journal; Peer Reviewed Journal]
Year of Publication
2007

146. **INCLUDE IN ARTICLE REVIEW (coded)**
The downside of self-management: A longitudinal study of the effects of conflict on trust, autonomy, and task interdependence in self-managing teams. [References].
Langfred, Claus W.
Academy of Management Journal. Vol.50(4), 2007, pp. 885-900.
[Journal; Peer Reviewed Journal]
Year of Publication
2007
Publication Month/Season
Aug

The very flexibility and adaptability that make self-managing teams effective can also be limiting and dysfunctional. I propose that self-managing teams may unintentionally restructure themselves inefficiently in response to conflict. Although detrimental consequences of conflict are normally considered as process-related, I explore possible structure-related effects. Specifically, I suggest that increased team conflict is associated with lower intrateam trust, which in turn may influence team structure by (1) reducing individual autonomy and (2) loosening task interdependencies in teams. This combination makes for a less than ideal team design. Longitudinal data from 35 self-managing teams support these expectations.

147. **EXCLUDE (The relationship and variation among leadership, team trust, and team performance I feel have been evaluated with other studies we have included. This study also looks specifically at the service and manufacturing industry, which limits applicability to CCCs - 2)**
The relationship of leadership, team trust and team performance: A comparison of the service and manufacturing industries. [References].
Shen, Ming-Jian; Chen, Ming-Chia.
Social Behavior and Personality. Vol.35(5), 2007, pp. 643-658.
[Journal; Peer Reviewed Journal]
Year of Publication
2007

The objective of this study was to investigate and compare the relationships and variations among leadership, team trust and team performance in the service and manufacturing industries. The results of using structural equation modeling to conduct hypotheses testing show that leadership has a positive effect on team trust and team performance, and that team trust also has a positive effect on team performance. By using MANOVA analysis to test for significant variances in leadership, team trust and team performance in the service and manufacturing industries, a significant variance was discovered in the testing of instructed leadership, relational trust and institutional trust in both industries.

148. **EXCLUDE (The group population studied in this article limit applicability and transferability to CCCs - 2)**
Trust and justice in the formation of joint consultative committees. [References].
Dietz, Graham; Fortin, Marion.
The International Journal of Human Resource Management. Vol.18(7), 2007, pp. 1159-1181.
[Journal; Peer Reviewed Journal]
Year of Publication
2007
Publication Month/Season
Jul

The paper identifies six phases in the creation of new joint staff-management consultative arrangements such as works councils or staff forums. Trust and justice theories are used to analyse the processes involved in initiating, designing, setting up and maintaining such a forum. The resulting framework considers both institutional and interpersonal aspects, and is intended to present researchers with a structure and an agenda for investigating the nature and consequences of the processes involved. The framework also provides initial guidelines to practitioners involved with establishing new consultative arrangements.

149. **EXCLUDE (This abstract was reviewed on the SCOPUS document)**
Social comparison-based thoughts in groups: Their associations with interpersonal trust and learning outcomes. [References].
Molleman, Eric; Nauta, Aukje; Buunk, Bram P.
Journal of Applied Social Psychology. Vol.37(6), 2007, pp. 1163-1180.
[Journal; Peer Reviewed Journal]
Year of Publication
2007
Publication Month/Season
Jun

150. **EXCLUDE (Although interesting, I don’t think age is going to be a major variable in our study, and this studied looked at trust from an economic perspective, which limits applicability to CCCs - 7)**
Trust and trustworthiness across different age groups. [References].
Sutter, Matthias; Kocher, Martin G.
Games and Economic Behavior. Vol.59(2), 2007, pp. 364-382.
[Journal; Peer Reviewed Journal]
Year of Publication
2007
Publication Month/Season
May

We examine the degree of trust and trustworthiness in an experimental trust game with 662 participants from six different age groups, ranging from 8-year-olds to retired persons. Although both trust and trustworthiness have been identified as fundamental pillars for efficient economic interactions, economic research has devoted little attention to measuring their strength in different age groups. In our experiment subjects interact with members of the same age group. We find that trust increases almost linearly from early childhood to early adulthood, but stays rather constant within different adult age groups. Trustworthiness prevails in all age groups.

152. **EXCLUDE (This study of virtual teams seems less applicable to CCCs than some of the other studies we have included, especially given the fact that it is over 12 years old - 7)**
Virtual Teams and Group Member Dissimilarity: Consequences for the Development of Trust. [References].
Krebs, Scott A; Hobman, Elizabeth V; Bordia, Prashant.
Small Group Research. Vol.37(6), 2006, pp. 721-741.
[Journal; Peer Reviewed Journal]
Year of Publication
2006
Publication Month/Season
Dec

The consequences of demographic dissimilarity for group trust in work teams was examined in a virtual (computer-mediated) and a face-to-face (FTF) environment. Demographic dissimilarity (based on age, gender, country of birth, enrolled degree) was predicted to be negatively associated with group trust in the FTF environment but not in the computer-mediated environment. Participants worked in small groups on a creative task for 3 consecutive days. In the computer-mediated environment, participants worked on the task for an hour per day. In the FTF environment, participants worked on the task for 20 minutes per day. Partial support was found for the effectiveness of computer-mediated groups in reducing the negative consequences of dissimilarity. Age dissimilarity was negatively related to trust in FTF groups but not in computer-mediated groups. Birthplace dissimilarity was positively related to trust in computer-mediated groups. Implications for the successful management of virtual teams are discussed.

154. **EXCLUDE (This study of virtual teams seems less applicable to CCCs than some of the other studies we have included, especially given the fact that it is over 12 years old - 7)**
All in due time: The development of trust in computer-mediated and face-to-face teams. [References].
Wilson, Jeanne M; Straus, Susan G; McEvily, Bill.
Organizational Behavior and Human Decision Processes. Vol.99(1), 2006, pp. 16-33.
[Journal; Peer Reviewed Journal]
Year of Publication
2006
Publication Month/Season
Jan

This study examines the development of trust and cooperation in computer-mediated and face-to-face teams. Fifty-two, three-person teams worked on a mixed-motive task over a 3-week period using computer-mediated or face-to-face interaction. Results showed that trust started lower in computer-mediated teams but increased to levels comparable to those in face-to-face teams over time. Furthermore, this pattern of results also held for teams that switched from face-to-face to electronic media and vice versa. Content analysis showed that high levels of inflammatory remarks were associated with slow trust development in computer-mediated teams. The results challenge prevailing assumptions about how trust develops in distributed teams and suggest modifications to established theories of computer-mediated communication.

155. **EXCLUDE (This study of virtual teams seems less applicable to CCCs than some of the other studies we have included, especially given the population studied (university students) and the fact that it is over 13 years old - 7)**
The rules of virtual groups: Trust, liking, and performance in computer-mediated communication. [References].
Walther, Joseph B; Bunz, Ulla.
Journal of Communication. Vol.55(4), 2005, pp. 828-846.
[Journal; Peer Reviewed Journal]
Year of Publication
2005
Publication Month/Season
Dec

Research on virtual groups reflects concerns about the development of trust and liking and about the performance of partners who do not see each other or work proximally. Previous studies have explored behaviors leading to subjectively experienced trust and/or liking, or trusting behaviors associated with group output, but have not linked behaviors, subjective affect, and output quality. Deriving principles from the social information processing theory of computer-mediated communication, this research identified and tested six communication rules for virtual groups. Employing a quasi-experimental procedure to maximize the variance in rule-following behavior, some distributed groups in a cross-university course were assigned to follow rules as pan of their grades on group assignments conducted using computer-mediated communication from which messages were collected and later coded. Through self-reported measures of rule following and affect, results reveal correlations between each rule with trust and liking. Less consistent are the relationships between rule following, specific observed behaviors, and actual performance quality. Interpretations suggest that a powerful set of collaboration rules has been identified or that the mere following of any rules and norms reduces uncertainty and enhances trust in distributed work teams.

156. **INCLUDE IN ARTICLE REVIEW (coded)**
The connection between trust and knowledge management: What are its implications for team performance. [References].
Politis, John D.
Journal of Knowledge Management. Vol.7(5), 2003, pp. 55-66.
[Journal; Peer Reviewed Journal]
Year of Publication
2003

The latest buzzwords in organizational change and development literature are "knowledge management" and "knowledge transfer", which proponents claim are successful ways of improving and enhancing employees' performance. Moreover, trust and the ability of employees to work in an autonomous manner are often cited as being essential for the effectiveness of self-managed teams. Little however, is known on the effect of interpersonal trust on knowledge management (acquisition) of team members, and the consequences for team performance. A survey of 49 self-managing teams was carried out to investigate the relationship between the dimensions of interpersonal trust, knowledge acquisition, and team performance. Overall, findings support that most interpersonal trust dimensions are positively related to the variables of knowledge acquisition. The results also showed that the effects of interpersonal trust on team performance to a large extent are mediated by the intervening variables of knowledge acquisition.

158. **INCLUDE IN ARTICLE REVIEW (coded)**
Work team trust and effectiveness. [References].
Costa, Ana Cristina.
Personnel Review. Vol.32(5), 2003, pp. 605-622.
[Journal; Peer Reviewed Journal]
Year of Publication
2003

This article aims to explore the nature and functioning of trust in work teams. Trust is defined as a multi-component variable with distinct but related dimensions. These include propensity to trust, perceived trustworthiness, co-operative and lack of monitoring behaviours. A model was tested relating trust with perceived task performance, team satisfaction, and two dimensions of organisational commitment, i.e. attitudinal and continuance. Survey data from 112 teams (n = 395) was collected in three social care institutions in The Netherlands. The results are supportive of a multi-component structure for trust and of its importance to the functioning of teams and organisations. Work team trust appeared strongly related with team member's attitudes towards the organisation. Trust between team members was positively associated with attitudinal commitment and negatively with continuance commitment. Trust was also positively related with perceived task performance and with team satisfaction. In addition, perceived task performance appeared strongly related to team satisfaction.

159. **INCLUDE IN ARTICLE REVIEW (This abstract was reviewed on the SCOPUS document)**
Leadership and trust facilitating cross-functional team success. [References].
Webber, Sheila Simsarian.
Journal of Management Development. Vol.21(3), 2002, pp. 201-214.
[Journal; Peer Reviewed Journal]
Year of Publication
2002

160. **EXCLUDE (This abstract was reviewed on the SCOPUS document)**
Group process & trust in group discussion. [References].
Wojnar, Linda; Uden, Lorna.
International Journal of Information and Communication Technology Education. Vol.1(1), 2005, pp. 55-68.
[Journal; Peer Reviewed Journal]
Year of Publication
2005

161. **EXCLUDE (This study of virtual teams seems less applicable to CCCs than some of the other studies we have included, especially given the fact that it is over 13 years old - 7)**
Managing distance in a global virtual team: The evolution of trust through technology-mediated relational communication. [References].
Henttonen, Kaisa; Blomqvist, Kirsimarja.
Strategic Change. Vol.14(2), 2005, pp. 107-119.
[Journal; Peer Reviewed Journal]
Year of Publication
2005
Publication Month/Season
Mar-Apr

1. Virtual teams offer the potential for the efficient combination of a dispersed workforce and the potential for leveraging diffuse knowledge and skills effectively for collaborative innovation. Information technology plays an important role in virtual teams, but virtual teamwork also involves significant social redesign. Trust is argued to be an important component in team development and effectiveness, and within this paper we explore the role and development of trust in the early stages of a virtual team. 2. On the basis of findings from a web-based questionnaire and interviews with global virtual team members in a major telecommunications company it appears that communications technology supports relationship building in tasks related to information sharing and storing and relational communication to a lesser degree. The antecedents of trust in the virtual team identified are similar to the antecedents of trust in a traditional collocated team context, and it appears that virtual team leaders and members approach virtual teams as an extension of traditional teamwork. 3. On the basis of the research results it is argued that relational communication and psychosocial factors such as trust, commitment and communication play an important role in the functioning of virtual teams. It is also suggested that where virtual team leaders and members attempt to approach virtual teams as an extension of traditional teamwork, many of the potential benefits may not be realized while much of the expense related to virtual teamwork remains.

162. **INCLUDE IN ARTICLE REVIEW (coded)**
The Influence of Relationship Conflict and Trust on the Transactive Memory: Performance Relation in Top Management Teams. [References].
Rau, Devaki.
Small Group Research. Vol.36(6), 2005, pp. 746-771.
[Journal; Peer Reviewed Journal]
Year of Publication
2005
Publication Month/Season
Dec

Recent research on top management teams examines the effects of team processes on performance. This study attempts to add to this literature by examining how social political relations such as conflict and trust among members of a top management team moderate the performance effects of the team's cognitive processes, as represented by its transactive memory. The results indicate that the location dimension of transactive memory positively influences performance for teams with low levels of relationship conflict but has no significant effect for teams with high levels of relationship conflict.

163. **EXCLUDE (This article looks at reciprocal trust between interacting teams. This really isn’t the focus of our study and the population studied (teams of systems analysts and design students) limit applicability and transferability to CCCs - 2).**
The reciprocal nature of trust: A longitudinal study of interacting teams. [References].
Serva, Mark A; Fuller, Mark A; Mayer, Roger C.
Journal of Organizational Behavior. Vol.26(6), 2005, pp. 625-648.
[Journal; Peer Reviewed Journal]
Year of Publication
2005
Publication Month/Season
Sep

This research develops and investigates the concept of reciprocal trust between interacting teams. Reciprocal trust is defined as the trust that results when a party observes the actions of another and reconsiders one's trust-related attitudes and subsequent behaviors based on those observations. Twenty-four teams of systems analysis and design students were involved in a 6-week controlled field study focused on the development of an information systems project. Each team was responsible for both developing a system (development role) and for supervising the development of a system by another team (management role). Risk-taking actions exhibited by one team in an interacting pair were found to predict the other team's trustworthiness perceptions and subsequent trust. The level of trust formed in turn predicted the team's subsequent risk-taking behaviors with respect to the other team. This pattern of reciprocal trust repeated itself as the teams continued to interact over the duration of the project, thus supporting our model of reciprocal trust. Findings also indicate that trust and trust formation can occur at the team level.

164. **EXCLUDE (This abstract was reviewed on the SCOPUS document)**
Short Communication: A social identity approach to trust: Interpersonal perception, group membership and trusting behaviour. [References].
Tanis, Martin; Postmes, Tom.
European Journal of Social Psychology. Vol.35(3), 2005, pp. 413-424.
[Journal; Peer Reviewed Journal]
Year of Publication
2005
Publication Month/Season
May-Jun

165. **INCLUDE IN ARTICLE REVIEW (coded)**
Trust in the Workplace: Factors Affecting Trust Formation Between Team Members. [References].
Spector, Michele D; Jones, Gwen E.
The Journal of Social Psychology. Vol.144(3), 2004, pp. 311-321.
[Journal; Peer Reviewed Journal]
Year of Publication
2004
Publication Month/Season
Jun

The authors used survey data from 127 professional-level employees working in 8 industries to assess the effects of respondent's trusting stance and (a) the trustee's organization membership (internal or external), (b) the hierarchical relationship (supervisor or peer), and (c) the gender of the trustee, on initial trust level for a new project team member. The authors found that trusting stance was positively related to initial trust level. The authors also found an interaction effect between respondent gender and trustee gender on initial trust. Specifically, male initial trust level was higher for a new male team member and lower for a new female team member. The present study provided additional understanding of the formation of initial trust levels and its importance for team functioning.

166. **EXCLUDE (This abstract was included on the SCOPUS document)**
Toward Contextualized Theories of Trust: The Role of Trust in Global Virtual Teams. [References].
Jarvenpaa, Sirkka L; Shaw, Thomas R; Staples, D. Sandy.
Information Systems Research. Vol.15(3), 2004, pp. 250-267.
[Journal; Peer Reviewed Journal]
Year of Publication
2004
Publication Month/Season
Sep

167. **EXCLUDE (We’ve included several studies on self-management teams, and I don’t think this one is as applicable to CCCs as some of the others we have included - 2)**
Too Much of a Good Thing? Negative Effects of High Trust and Individual Autonomy in Self-Managing Teams. [References].
Langfred, Claus W.
Academy of Management Journal. Vol.47(3), 2004, pp. 385-399.
[Journal; Peer Reviewed Journal]
Year of Publication
2004
Publication Month/Season
Jun

A high level of trust can make the members of self-managing work teams reluctant to monitor one another. If low monitoring combines with high individual autonomy, team performance can suffer. Data from 71 self-managing teams of MBA students demonstrated this effect. High trust was associated with higher team performance when individual autonomy was low but with lower performance when individual autonomy was high. Additional analysis showed a moderated mediating role of monitoring and autonomy in the relationship between trust and performance.

168. **INCLUDE IN ARTICLE REVIEW (coded)**
The dynamic relationship between performance feedback, trust, and conflict in groups: A longitudinal study. [References].
Peterson, Randall S; Behfar, Kristin Jackson.
Organizational Behavior and Human Decision Processes. Vol.92(1-2), 2003, pp. 102-112.
[Journal; Peer Reviewed Journal]
Year of Publication
2003
Publication Month/Season
Sep-Nov

Moderate task conflict has generally been associated with higher group performance, and relationship conflict associated with lower performance. Past studies have most often discussed their findings as though differences in level of intragroup conflict cause differences in group performance-rather than testing the additional possibility that reported group conflict is a reaction to feedback on past group performance. This paper explores the dynamic relationships between intragroup conflict and performance with a longitudinal design. Results from 67 groups suggest that initial performance feedback to groups can have significant consequences for future team interaction. We find evidence to suggest that, (a) negative initial group performance feedback results in later increases in both task and relationship conflict, but that (b) groups with high early intragroup trust are buffered from experiencing the worst of future relationship conflict.

169. **EXCLUDE (The abstract is not very well written, sentences start with Suggests.,, This does not look like a high tier journal with meaningful content that would contribute to our study - 2)**
The relationship between trust and team performance. [References].
Erdem, Ferda; Ozen, Janset; Atsan, Nuray.
Work Study: A Journal of Productivity Science. Vol.52(6-7), 2003, pp. 337-340.
[Journal; Peer Reviewed Journal]
Year of Publication
2003

Suggests that teamworking is a critical success factor for most organizations. The study reported in this paper sought to investigate the relationship between the level of trust between members of a workteam and the performance of that team. The study involved 148 members of 28 teams across four organisations. Suggests that though there is a relationship between trust and performance (which was found to be particularly strong within two of the organizations), there are other factors at play. Suggests a number of factors which require further investigation.

170. **INCLUDE IN ARTICLE REVIEW (coded)**
Socialization and trust in work groups. [References].
Moreland, Richard L; Levine, John M.
Group Processes & Intergroup Relations. Vol.5(3), 2002, pp. 185-201.
[Journal; Peer Reviewed Journal]
Year of Publication
2002
Publication Month/Season
Jul

Several theoretical analyses of trust in organizations have been offered, but the social context in which that trust operates is often ignored. Our analysis examines trust in work groups, with a focus on changes in such groups over time. Socialization is an important form of temporal change in work groups. We examine issues of trust that can arise for full members of groups with new or marginal members. One such issue is how much a group's full members can trust its new and marginal members, who belong to the group, but are not fully accepted by it. A related issue is how much full members can trust each other around any new or marginal members, whose thoughts, feelings, and behavior must be carefully monitored and shaped before they gain (or regain) the group's acceptance. After analyzing both of these issues in some detail, we close by identifying several other issues of trust that can arise in work groups as they change over time.

171. **EXCLUDE (This article was reviewed on the SCOPUS document)**
Communication and trust in global virtual teams. [References].
Jarvenpaa, Sirkka L; Leidner, Dorothy E.
Journal of Computer-Mediated Communication. Vol.3(4), 1998, pp. No Pagination Specified.
[Journal; Peer Reviewed Journal]
Year of Publication
1998
Publication Month/Season
Jun

172. **EXCLUDE (This article focuses on transformational leadership. Although this may be important in a work or project team, the context it is used in this study and the idea of an iron cage makes this less applicable to transferable to CCCs - 2)**
Transformational leadership or the iron cage: Which predicts trust, commitment and team efficacy? [References].
Arnold, Kara A; Barling, Julian; Kelloway, E. Kevin.
Leadership & Organization Development Journal. Vol.22(7), 2001, pp. 315-320.
[Journal; Peer Reviewed Journal]
Year of Publication
2001

This paper investigates the differential effects of transformational leadership and the "iron cage" on trust, commitment and team efficacy at the team level. Transformational leadership has been shown to have positive effects on trust, commitment and team efficacy. However, it could be argued that these results are not due to the leadership but to the idea that the team has developed strong norms that constrain their behavior and "force" them to perform. The rival hypothesis that the iron cage results in trust, commitment and team efficacy is tested using hierarchical regression analysis. We find that transformational leadership in teams predicts trust, commitment and team efficacy over and beyond the perceptions of the iron cage. The iron cage adds to the prediction of commitment only. Results suggest that while encouraging strong values and norms within a team will lead to increased commitment, focusing on transformational leadership in teams is a more effective way to encourage the development of trust, commitment and team efficacy.

173. **INCLUDE IN ARTICLE REVIEW (coded)**
Trust within teams: The relation with performance effectiveness. [References].
Costa, Ana Cristina; Roe, Robert A; Taillieu, Tharsi.
European Journal of Work and Organizational Psychology. Vol.10(3), 2001, pp. 225-244.
[Journal; Peer Reviewed Journal]
Year of Publication
2001
Publication Month/Season
Sep

The acknowledgement that trust is important for the functioning of organizations has increased the demand for research showing how this importance is reflected on the behaviour of its members. In this article we focus on trust within teams and explore the relation with performance effectiveness. A model was tested relating trust with perceived task performance, team satisfaction, relationship commitment, and stress. In this model trust is presented as a multi-component variable with distinct but related dimensions. These include propensity to trust, perceived trustworthiness, cooperative and monitoring behaviours. Data from 112 teams collected in three social care institutions in The Netherlands were analysed with structural equation modelling to test the model. The results are supportive of the multi-component structure of trust and confirmed the importance of trust for the functioning of teams in organizations. The results suggest that trust is positively related with perceived task performance, team satisfaction, and relationship commitment, and negatively related with stress. In addition, perceived task performance was positively related with team satisfaction.

176. **INCLUDE IN ARTICLE REVIEW (This abstract was reviewed on the PUBMED document)**
Task conflict and relationship conflict in top management teams: The pivotal role of intragroup trust. [References].
Simons, Tony L; Peterson, Randall S.
Journal of Applied Psychology. Vol.85(1), 2000, pp. 102-111.
[Journal; Peer Reviewed Journal]
Year of Publication
2000
Publication Month/Season
Feb

177. **EXCLUDE (This abstract was reviewed on the SCOPUS document)**
Communication and trust in global virtual teams. [References].
Jarvenpaa, Sirkka L; Leidner, Dorothy E.
Organization Science. Vol.10(6), 1999, pp. 791-815.
[Journal; Peer Reviewed Journal]
Year of Publication
1999
Publication Month/Season
Nov-Dec

178. **EXCLUDE (This study is almost 20 years old and doesn’t really seem to add any additional information that would be useful to our review - 2)**
The effects of interpersonal trust on work group performance. [References].
Dirks, Kurt T.
Journal of Applied Psychology. Vol.84(3), 1999, pp. 445-455.
[Journal; Peer Reviewed Journal]
Year of Publication
1999
Publication Month/Season
Jun

This study explored 2 questions: Does the level of trust within a group affect group performance? If so, how does this relationship operate? An experimental method was used to examine 2 roles through which interpersonal trust could affect group performance: a main effect and a moderating effect. The data do not support the main effect that has dominated the literature on interpersonal trust. The data do support the moderating role: Trust seems to influence how motivation is converted into work group processes and performance. On the basis of these findings, it is suggested that trust may be best understood as a construct that influences group performance indirectly by channeling group members' energy toward reaching alternative goals.

179. **EXCLUDE (The concept of video-mediated communication in this study is very dated compared to studies we are proposing to include looking at virtual teams. I think this study adds little value to our review - 7)**
Teams without trust? Investigations in the influence of video-mediated communication on the origin of trust among cooperating persons. [References].
Muhlfelder, M; Klein, U; Simon, S; Luczak, H.
Behaviour & Information Technology. Vol.18(5), 1999, pp. 349-360.
[Journal; Peer Reviewed Journal]
Year of Publication
1999
Publication Month/Season
Sep-Oct

Based on a model about the origin of trust among persons, who are meeting for the first time, the influence of video-mediated communication on confidence building was tested. Two groups were compared, one carrying out a cooperative task face-to-face, the other using a video-conferencing system. Ss were aged 21-36 yrs. Three measurements were taken. Measurement 1 registered the effects of anticipation. One group was instructed to get to know somebody face-to-face, the other group should meet their partner by videoconference. Measurement 2 registered the amount of trust the subjects had developed in their partner during the session. Measurement 3 was the analysis of the interpersonal interaction processes during the conversation. The results were that Ss who anticipated a video-conference showed no difference in mean, but a highly reduced variability in their assessment of their counterpart compared to those subjects who anticipated a meeting face-to-face. A similar pattern was observed for measurement 2. However, no systematically meaningful difference in the behavioral categories of measurement 3 could be found. The results are similar to experiments mostly carried out in the 1970s and 1980s, which focused on comparisons between face-to-face and mediated communication.

180. **INCLUDE IN ARTICLE REVIEW (coded)**
Building commitment, attachment, and trust in strategic decision-making teams: The role of procedural justice.
Korsgaard, M. Audrey; Schweiger, David M; Sapienza, Harry J.
Academy of Management Journal. Vol.38(1), 1995, pp. 60-84.
[Journal; Peer Reviewed Journal]
Year of Publication
1995
Publication Month/Season
Feb

Examined how decision-making procedures can facilitate the positive attitudes necessary for cooperative relations in decision-making teams. It was hypothesized that consideration of member input and members' influence on a decision affects their perceptions of procedural fairness and consequently, their commitment to the decision, attachment to the group, and trust in its leader. An experiment involving 20 intact management teams (including 20 team leaders and 89 team members) supported the hypotheses and indicated that perceived fairness partially mediated the impact of procedures on commitment, attachment, and trust.

**Exclude (these articles have already been excluded based upon the title review):**

8. **EXCLUDE (e-commerce; not applicable or readily transferable to CCCs - 6)**
The stickiness intention of group-buying websites: The integration of the commitment-trust theory and e-commerce success model. [References].
Wang, Wei-Tsong; Wang, Yi-Shun; Liu, En-Ru.
Information & Management. Vol.53(5), 2016, pp. 625-642.
[Journal; Peer Reviewed Journal]
Year of Publication
2016
Publication Month/Season
Jul

12. **EXCLUDE (population studied/sports; not applicable or readily transferable to CCCs - 2)**
Coaching competency and trust in coach in sport teams. [References].
Kao, San-Fu; Hsieh, Ming-Hui; Lee, Po-Lun.
International Journal of Sports Science & Coaching. Vol.12(3), 2017, pp. 319-327.
[Journal; Peer Reviewed Journal]
Year of Publication
2017
Publication Month/Season
Jun

13. **EXCLUDE (technology, principal-agent relationships?; not applicable or readily transferable to CCCs - 3)**
The effect of production technology on trust and reciprocity in principal-agent relationships with team production. [References].
Cobo-Reyes, Ramon; Lacomba, Juan A; Lagos, Francisco; Levin, Dan.
Journal of Economic Behavior & Organization. Vol.137 2017, pp. 324-338.
[Journal; Peer Reviewed Journal]
Year of Publication
2017
Publication Month/Season
May

16. **EXCLUDE (substance abuse; not applicable or readily transferable to CCCs - 3)**
Effects of live and educational music therapy on working alliance and trust with patients on detoxification unit: A four-group cluster-randomized trial. [References].
Silverman, Michael J.
Substance Use & Misuse. Vol.51(13), 2016, pp. 1741-1750.
[Journal; Peer Reviewed Journal]
Year of Publication
2016
Publication Month/Season
Nov

19. **EXCLUDE (I’ve reviewed this article, heavy focus on computer science; not applicable or readily transferable to CCCs - 6)**
A visual interaction consensus model for social network group decision making with trust propagation. [References].
Wu, Jian; Chiclana, Francisco; Fujita, Hamido; Herrera-Viedma, Enrique.
Knowledge-Based Systems. Vol.122 2017, pp. 39-50.
[Journal; Peer Reviewed Journal]
Year of Publication
2017
Publication Month/Season
Apr

22. **EXCLUDE (ethics, population studied; not applicable or readily transferable to CCCs - 3)**
Can I trust them to do everything? The role of distrust in ethics committee consultations for conflict over life-sustaining treatment among Afro-Caribbean patients. [References].
Romain, Frederic; Courtwright, Andrew.
Journal of Medical Ethics: Journal of the Institute of Medical Ethics. Vol.42(9), 2016, pp. 582-585.
[Journal; Peer Reviewed Journal]
Year of Publication
2016
Publication Month/Season
Sep
24. **EXCLUDE (hormones, automated agents?; not applicable or readily transferable to CCCs - 4)**
A little anthropomorphism goes a long way: Effects of oxytocin on trust, compliance, and team performance with automated agents. [References].
de Visser, Ewart J; Monfort, Samuel S; Goodyear, Kimberly; Lu, Li; O'Hara, Martin; Lee, Mary R; Parasuraman, Raja; Krueger, Frank.
Human Factors. Vol.59(1), 2017, pp. 116-133.
[Journal; Peer Reviewed Journal]
Year of Publication
2017
Publication Month/Season
Feb

25. **EXCLUDE (computer science focus; not applicable or readily transferable to CCCs - 6)**
Uninorm trust propagation and aggregation methods for group decision making in social network with four tuple information. [References].
Wu, Jian; Xiong, Ruoyun; Chiclana, Francisco.
Knowledge-Based Systems. Vol.96 2016, pp. 29-39.
[Journal; Peer Reviewed Journal]
Year of Publication
2016
Publication Month/Season
Mar

27. **EXCLUDE (culture of crime?; not applicable or readily transferable to CCCs - 2)**
Trust and in-group favoritism in a culture of crime. [References].
Meier, Stephan; Pierce, Lamar; Vaccaro, Antonino; La Cara, Barbara.
Journal of Economic Behavior & Organization. Vol.132(Part A), 2016, pp. 78-92.
[Journal; Peer Reviewed Journal]
Year of Publication
2016
Publication Month/Season
Dec

40. **EXCLUDE (subject/population studied; not applicable or readily transferable to CCCs - 3)**
An intermediary enhances out-group trust and in-group profit expectation of Chinese but not Australians. [References].
Ye, Jiawen; Ng, Sik Hung.
International Journal of Psychology. Vol.52(3), 2017, pp. 189-196.
[Journal; Peer Reviewed Journal]
Year of Publication
2017
Publication Month/Season
Jun

41. **EXCLUDE (subject/population studied; not applicable or readily transferable to CCCs - 3)**
Quality of physical surroundings and service encounters, airfare, trust and intention during the flight: Age-group difference (young, middle-aged, and mature). [References].
Han, Heesup; Hwang, Jinsoo.
International Journal of Contemporary Hospitality Management. Vol.27(4), 2015, pp. 585-607.
[Journal; Peer Reviewed Journal]
Year of Publication
2015

47. **EXCLUDE (ropes course?; not applicable or readily transferable to CCCs - 2)**
Getting roped in: Group cohesion, trust, and efficacy following a ropes course intervention. [References].
Eatough, Erin; Chang, Chu-Hsiang; Hall, Nicholas.
Performance Improvement Quarterly. Vol.28(2), 2015, pp. 65-89.
[Journal; Peer Reviewed Journal]
Year of Publication
2015

52. **EXCLUDE (subject/population studied; not applicable or readily transferable to CCCs - 3)**
Psychometric analysis of a scale to assess particularized trust in families and community- and congregation-based groups. [References].
Miller, Margaret C; Pope, Holly C; Wolfer, Terry A; Mann, Joshua R; Hussey, James R; Colabianchi, Natalie; McKeown, Robert E.
Journal of Community Psychology. Vol.43(2), 2015, pp. 227-243.
[Journal; Peer Reviewed Journal]
Year of Publication
2015
Publication Month/Season
Mar

55. **EXCLUDE (subject/population studied; not applicable or readily transferable to CCCs - 2)**
Empirical analysis of roles of perceived leadership styles and trust on team members' creativity: Evidence from Korean ICT companies. [References].
Jo, Nam Yong; Lee, Kun Chang; Lee, Dae Sung; Hahn, Minhee.
Computers in Human Behavior. Vol.42 2015, pp. 149-156.
[Journal; Peer Reviewed Journal]
Year of Publication
2015
Publication Month/Season
Jan

56. **EXCLUDE (child psychology; not applicable or readily transferable to CCCs - 4)**
Do children trust based on group membership or prior accuracy? The role of novel group membership in children's trust decisions. [References].
Elashi, Fadwa B; Mills, Candice M.
Journal of Experimental Child Psychology. Vol.128 2014, pp. 88-104.
[Journal; Peer Reviewed Journal]
Year of Publication
2014
Publication Month/Season
Dec

57. **EXCLUDE (subject/population studied; not applicable or readily transferable to CCCs - 3)**
Group psychotherapy's impact on trust in veterans with PTSD: A pilot study. [References].
Williams, Wright; Graham, David P; McCurry, Katherine; Sanders, April; Eiseman, Jessica; Chiu, Pearl H; King-Casas, Brooks.
Bulletin of the Menninger Clinic. Vol.78(4), 2014, pp. 335-348.
[Journal; Peer Reviewed Journal]
Year of Publication
2014
Publication Month/Season
Dec

60. **EXCLUDE (computer science emphasis; not applicable or readily transferable to CCCs - 6)**
A social network analysis trust-consensus based approach to group decision-making problems with interval-valued fuzzy reciprocal preference relations. [References].
Wu, Jian; Chiclana, Francisco.
Knowledge-Based Systems. Vol.59 2014, pp. 97-107.
[Journal; Peer Reviewed Journal]
Year of Publication
2014
Publication Month/Season
Mar

64. **EXCLUDE (online, offline social lending groups?; not applicable or readily transferable to CCCs - 6)**
Innovative transposition of trust mechanisms in social lending groups from offline to online. [References].
Assadi, Djamchid; Ashta, Arvind.
Strategic Change. Vol.23(7-8), 2014, pp. 461-480.
[Journal; Peer Reviewed Journal]
Year of Publication
2014
Publication Month/Season
Nov

66. **EXCLUDE (subject/population studied; not applicable or readily transferable to CCCs - 3)**
Effects of a live educational music therapy intervention on acute psychiatric inpatients' perceived social support and trust in the therapist: A four-group randomized effectiveness study. [References].
Silverman, Michael J.
Journal of Music Therapy. Vol.51(3), 2014, pp. 228-249.
[Journal; Peer Reviewed Journal]
Year of Publication
2014
Publication Month/Season
Fal

67. **EXCLUDE (swift trust, especially in virtual teams not really the focus of our review - 6)**
The role of swift trust in virtual teams and implications for human resource development. [References].
Germain, Marie-Line; McGuire, David.
Advances in Developing Human Resources. Vol.16(3), 2014, pp. 356-370.
[Journal; Peer Reviewed Journal]
Year of Publication
2014
Publication Month/Season
Aug

68. **EXCLUDE (retraction statement, likely commentary or editorial, not primary research or a review article - 6)**
Retraction statement: "Authentically leading groups: The mediating role of collective psychological capital and trust." [References].
Walumba, Fred O; Luthans, Fred; Avey, James B; Oke, Adegoke.
Journal of Organizational Behavior. Vol.35(5), 2014, pp. 746.
[Journal; Peer Reviewed Journal]
Year of Publication
2014
Publication Month/Season
Jul

69. **EXCLUDE (computer science emphasis/online group-buying?; not applicable or readily transferable to CCCs - 6)**
Determinants of repurchase intention in online group-buying: The perspectives of DeLone & McLean IS success model and trust. [References].
Hsu, Meng-Hsiang; Chang, Chun-Ming; Chu, Kuo-Kuang; Lee, Yi-Jung.
Computers in Human Behavior. Vol.36 2014, pp. 234-245.
[Journal; Peer Reviewed Journal]
Year of Publication
2014
Publication Month/Season
Jul

70. **EXCLUDE (subject/population studied, politics; not applicable or readily transferable to CCCs - 3)**
Out-group trust and conflict understandings: The perspective of Turks and Kurds in Turkey. [References].
Celebi, Elif; Verkuyten, Maykel; Kose, Talha; Maliepaard, Mieke.
International Journal of Intercultural Relations. Vol.40 2014, pp. 64-75.
[Journal; Peer Reviewed Journal]
Year of Publication
2014
Publication Month/Season
May

73. **EXCLUDE (computer science emphasis, esp “virtual world environment; not applicable or readily transferable to CCCs - 6)**
Institutional boundaries and trust of virtual teams in collaborative design: An experimental study in a virtual world environment. [References].
Schiller, Shu Z; Mennecke, Brian E; Nah, Fiona Fui-Hoon; Luse, Andy.
Computers in Human Behavior. Vol.35 2014, pp. 565-577.
[Journal; Peer Reviewed Journal]
Year of Publication
2014
Publication Month/Season
Jun

76. **EXCLUDE (online gaming? brand trust/product type?; not applicable or readily transferable to CCCs - 6)**
Online game characters' influence on brand trust: Self-disclosure, group membership, and product type. [References].
Choi, Yung Kyun; Yoon, Sukki; Lacey, Heather P.
Journal of Business Research. Vol.66(8), 2013, pp. 996-1003.
[Journal; Peer Reviewed Journal]
Year of Publication
2013
Publication Month/Season
Aug

77. **EXCLUDE (game situation?; subject/population studied; not applicable or readily transferable to CCCs - 2)**
Group membership overrides dispositional variables in the determination of reciprocation of trust in a game situation. [References].
Sakalaki, Maria; Richardson, Clive; Sotiriou, Penelope.
Revue Internationale de Psychologie Sociale. Vol.26(4), 2013, pp. 79-92.
[Journal; Peer Reviewed Journal]
Year of Publication
2013

85. **EXCLUDE (computer science emphasis - personal profiles/virtual project teams; subject/population studied; not applicable or readily transferable to CCCs - 6)**
The mind's eye on personal profiles: A cognitive perspective on profile elements that inform initial trustworthiness assessments and social awareness in virtual project teams. [References].
Rusman, Ellen; van Bruggen, Jan; Sloep, Peter; Valcke, Martin; Koper, Rob.
Computer Supported Cooperative Work (CSCW). Vol.22(2-3), 2013, pp. 159-179.
[Journal; Peer Reviewed Journal]
Year of Publication
2013
Publication Month/Season
Apr

86. **EXCLUDE (swift trust, especially in virtual teams not really the focus of our review - 6)**
Swift trust in global virtual teams: Trusting beliefs and normative actions. [References].
Crisp, C. Brad; Jarvenpaa, Sirkka L.
Journal of Personnel Psychology. Vol.12(1), 2013, pp. 45-56.
[Journal; Peer Reviewed Journal]
Year of Publication
2013

88. **EXCLUDE (repairing trust not the focus of our review - 2)**
Repairing trust to preserve balance: A balance-theoretic approach to trust breach and repair in groups. [References].
Brodt, Susan E; Neville, Lukas.
Negotiation and Conflict Management Research. Vol.6(1), 2013, pp. 49-65.
[Journal; Peer Reviewed Journal]
Year of Publication
2013
Publication Month/Season
Feb

89. **EXCLUDE (repairing trust not the focus of our review - 2)**
Repairing trust with individuals vs. groups. [References].
Kim, Peter H; Cooper, Cecily D; Dirks, Kurt T; Ferrin, Donald L.
Organizational Behavior and Human Decision Processes. Vol.120(1), 2013, pp. 1-14.
[Journal; Peer Reviewed Journal]
Year of Publication
2013
Publication Month/Season
Jan

93. **EXCLUDE (deception in the setting of virtual teams not the focus of our review - 6)**
An examination of deception in virtual teams: Effects of deception on task performance, mutuality, and trust. [References].
Fuller, Christie M; Marett, Kent; Twitchell, Douglas P.
IEEE Transactions on Professional Communication. Vol.55(1), 2012, pp. 20-35.
[Journal; Peer Reviewed Journal]
Year of Publication
2012
Publication Month/Season
Mar

102. **EXCLUDE (linguistic politeness in emails, esp in student-teams not the focus of our review - 4)**
Linguistic politeness in student-team emails: Its impact on trust between leaders and members. [References].
Lam, Chris.
IEEE Transactions on Professional Communication. Vol.54(4), 2011, pp. 360-375.
[Journal; Peer Reviewed Journal]
Year of Publication
2011
Publication Month/Season
Dec

105. **EXCLUDE (human rights and trust in the out-group not the focus of our review; not applicable or readily transferable to CCCs - 4)**
Explaining support for violating out-group human rights in conflict: Attitudes toward principles of human rights, trust in the out-group, and intergroup contact. [References].
Maoz, Ifat; McCauley, Clark.
Journal of Applied Social Psychology. Vol.41(4), 2011, pp. 891-905.
[Journal; Peer Reviewed Journal]
Year of Publication
2011
Publication Month/Season
Apr

124. **EXCLUDE (politics; not applicable or readily transferable to CCCs - 3)**
Intergroup communication as a predictor of Jewish-Israeli agreement with integrative solutions to the Israeli-Palestinian conflict: The mediating effects of out-group trust and guilt. [References].
Maoz, Ifat; Ellis, Donald G.
Journal of Communication. Vol.58(3), 2008, pp. 490-507.
[Journal; Peer Reviewed Journal]
Year of Publication
2008
Publication Month/Season
Sep

127. **EXCLUDE (politics; not applicable or readily transferable to CCCs - 3)**
Institutional incentives and trust: Marginalized groups and the creation of trust in Local Government. [References].
Richey, Sean; Ikeda, Ken'ichi.
Social Science Quarterly. Vol.90(4), 2009, pp. 911-926.
[Journal; Peer Reviewed Journal]
Year of Publication
2009
Publication Month/Season
Dec

128. **EXCLUDE (mobile banking, information system success instruments; not applicable or readily transferable to CCCs - 6)**
Effect of trust level on mobile banking satisfaction: A multi-group analysis of information system success instruments. [References].
Chung, Namho; Kwon, Soon Jae.
Behaviour & Information Technology. Vol.28(6), 2009, pp. 549-562.
[Journal; Peer Reviewed Journal]
Year of Publication
2009
Publication Month/Season
Nov

138. **EXCLUDE (although the title mentions virtual team performance, online learning not the focus of our review - 6)**
The effects of cognitive thinking styles, trust, conflict management on online students' learning and virtual team performance. [References].
Liu, Xiaojing; Magjuka, Richard J; Lee, Seung-hee.
British Journal of Educational Technology. Vol.39(5), 2008, pp. 829-846.
[Journal; Peer Reviewed Journal]
Year of Publication
2008
Publication Month/Season
Sep

144. **EXCLUDE (subject/population studied; not applicable or readily transferable to CCCs - 4)**
Guanxi networks and members' effectiveness in Chinese work teams: Mediating effects of trust networks. [References].
Chou, Li-Fang; Cheng, Bor-Shiuan; Huang, Min-Ping; Cheng, Hung-Yueh.
Asian Journal of Social Psychology. Vol.9(2), 2006, pp. 79-95.
[Journal; Peer Reviewed Journal]
Year of Publication
2006
Publication Month/Season
Aug

151. **EXCLUDE (computer science emphasis; not applicable or readily transferable to CCCs - 6)**
Web-based template-driven communication support systems: Using shadow networkspace to support trust development in virtual teams. [References].
Remidez, Herbert Jr.; Stam, Antonie; Laffey, James M.
International Journal of e-Collaboration. Vol.3(1), 2007, pp. 65-83.
[Journal; Peer Reviewed Journal]
Year of Publication
2007
Publication Month/Season
Jan-Mar

153. **EXCLUDE (politics; not applicable or readily transferable to CCCs - 3)**
Exploring Comparative Ratings and Constituent Facets of Public Trust in Risk Regulatory Bodies and Related Stakeholder Groups. [References].
Weyman, Andrew K; Pidgeon, Nicholas F; Walls, John; Horlick-Jones, Tom.
Journal of Risk Research. Vol.9(6), 2006, pp. 605-622.
[Journal; Peer Reviewed Journal]
Year of Publication
2006
Publication Month/Season
Sep

157. **EXCLUDE (temporary virtual teams not the focus of our review - 6)**
Trust and temporary virtual teams: Alternative explanations and dramaturgical relationships. [References].
Panteli, Niki; Duncan, Elizabeth.
Information Technology & People. Vol.17(4), 2004, pp. 423-441.
[Journal; Peer Reviewed Journal]
Year of Publication
2004

174. **EXCLUDE (population studied; not applicable or readily transferable to CCCs - 3)**
Public trust and confidence in legal authorities: What do majority and minority group members want from the law and legal institutions? [References].
Tyler, Tom R.
Behavioral Sciences & the Law. Vol.19(2), 2001, pp. 215-235.
[Journal; Peer Reviewed Journal]
Year of Publication
2001

175. **EXCLUDE (population studied/sports; not applicable or readily transferable to CCCs - 2)**
Trust in leadership and team performance: Evidence from NCAA basketball. [References].
Dirks, Kurt T.
Journal of Applied Psychology. Vol.85(6), 2000, pp. 1004-1012.
[Journal; Peer Reviewed Journal]
Year of Publication
2000
Publication Month/Season
Dec
