# Supplementary material for: Trust in Group Decisions: a scoping review
Source: BMC Med Educ. 2019 Aug 14;19:309. doi: 10.1186/s12909-019-1726-4 (PMC6693175; doi:10.1186/s12909-019-1726-4)
Supplement: Supplementary file 5 — PUBMED Search Publication Review. (DOCX 43 kb) [file 12909_2019_1726_MOESM5_ESM.docx]

**Additional file 5: PUBMED, 7 April 2018 (titles), 16 April 2018 (abstracts), 12 June 2018 (re-review abstracts)**

**Search strategy:** ("Trust"[Majr] OR trust[ti] OR trustworth*[ti]) AND ("Group Processes"[Majr] OR group[ti] OR groups[ti] OR team[ti] OR teams[ti] OR committee[ti] OR committees[ti] OR jury[ti] OR juries[ti]) AND ("Decision Making"[Mesh] OR decision*[tiab] OR success[tiab] OR outcome*[tiab]) AND English[lang]: **61 results, 47 “exclude,” 14 “include for abstract review”**

**16 April 2018: 14 “include for abstract review,” 10 “exclude,” 4 “include in article review”**

**ABSTRACT REVIEW (14 articles):**

**INCLUDE IN ARTICLE REVIEW (coded)** 5: Kweekel L, Gerrits T, Rijnders M, Brown P. The Role of Trust in CenteringPregnancy: Building Interpersonal Trust Relationships in Group-Based

Prenatal Care in The Netherlands. Birth. 2017 Mar;44(1):41-47. doi: 10.1111/birt.12260. Epub 2016 Nov 7. PubMed PMID: 28198099.

### **Abstract**

#### **BACKGROUND:**

CenteringPregnancy (CP) is a specific model of group-based prenatal care for women, implemented in 44 midwifery practices in The Netherlands since 2011. Women have evaluated CP positively, especially in terms of social support, and improvements have been made in birthweight and preterm-birth outcomes; however, there is limited understanding as to why. The purpose of this study was to examine the mechanisms that create trusting relationships within CP to better understand CP outcomes and effectiveness.

#### **METHODS:**

A qualitative study was conducted using in-depth interviews with 26 (former) CP participants, alongside observations of CP sessions. All interviews were transcribed and analyzed following open, axial, and selective coding.

#### **RESULTS:**

Most women characterized trust as a positive expectation about how others would respond to sensitive information that was shared within the group. Trust emerged within the data as a multidimensional concept and several preconditions seemed crucial in building trusting relations: vulnerability, communication, reciprocity, chemistry, and atmosphere. The facilitating of interpersonal trust among CP participants enhanced group processes, especially as a basis for social support by which women said they were more eager to share sensitive information in a trusting environment.

#### **CONCLUSIONS:**

Processes of trust were interwoven within various CP group dynamics. Trust facilitated social support which in turn enabled reassurance and the building of women's self-confidence.

**EXCLUDE (This study seems focused on assigning peer reviews to review research and/or funding proposals. I don’t see any specific details about group process, dynamics, decision-making, etc. - 3)** 12: Li L, Wang Y, Liu G, Wang M, Wu X. Context-Aware Reviewer Assignment for Trust Enhanced Peer Review. PLoS One. 2015 Jun 19;10(6):e0130493. doi:10.1371/journal.pone.0130493. eCollection 2015. PubMed PMID: 26090849; PubMed Central PMCID: PMC4474895.

### Abstract

Reviewer assignment is critical to peer review systems, such as peer-reviewed research conferences or peer-reviewed funding applications, and its effectiveness is a deep concern of all academics. However, there are some problems in existing peer review systems during reviewer assignment. For example, some of the reviewers are much more stringent than others, leading to an unfair final decision, i.e., some submissions (i.e., papers or applications) with better quality are rejected. In this paper, we propose a context-aware reviewer assignment for trust enhanced peer review. More specifically, in our approach, we first consider the research area specific expertise of reviewers, and the institution relevance and co-authorship between reviewers and authors, so that reviewers with the right expertise are assigned to the corresponding submissions without potential conflict of interest. In addition, we propose a novel cross-assignment paradigm, and reviewers are cross-assigned in order to avoid assigning a group of stringent reviewers or a group of lenient reviewers to the same submission. More importantly, on top of them, we propose an academic CONtext-aware expertise relevanCe oriEnted Reviewer cross-assignmenT approach (CONCERT), which aims to effectively estimate the "true" ratings of submissions based on the ratings from all reviewers, even though no prior knowledge exists about the distribution of stringent reviewers and lenient reviewers. The experiments illustrate that compared with existing approaches, our proposed CONCERT approach can less likely assign more than one stringent reviewers or lenient reviewers to a submission simultaneously and significantly reduce the influence of ratings from stringent reviewers and lenient reviewers, leading to trust enhanced peer review and selection, no matter what kind of distributions of stringent reviewers and lenient reviewers are.

**EXCLUDE (See above, also this has no abstract to review, and I don’t feel justified pull the full text given the title of the article - 7)** 14: Anderson WP. Peer review: maintaining trust in research funding decisions. Med J Aust. 2015 Feb 2;202(2):65. PubMed PMID: 25627723.

No abstract available

**INCLUDE IN ARTICLE REVIEW (coded)** 15: Bianchi EC, Brockner J, van den Bos K, Seifert M, Moon H, van Dijke M, De Cremer D. Trust in decision-making authorities dictates the form of the

interactive relationship between outcome fairness and procedural fairness. Pers

Soc Psychol Bull. 2015 Jan;41(1):19-34. doi: 10.1177/0146167214556237. Epub 2014

Nov 11. PubMed PMID: 25387762.

### Abstract

Reactions to decisions are shaped by both outcome and procedural fairness. Moreover, outcome and procedural fairness interact to influence beliefs and behaviors. However, different types of "process/outcome" interaction effects have emerged. Many studies have shown that people react particularly negatively when they receive unfair or unfavorable outcomes accompanied by unfair procedures (the "low-low" interactive pattern). However, others find that people react especially positively when they receive fair or favorable outcomes accompanied by fair procedures (the "high-high" interactive pattern). We propose that trust in decision-making authorities dictates the form of the process/outcome interaction. Across three studies, when trust was high, the "low-low" interactive pattern emerged. When trust was low, the "high-high" interactive pattern emerged. The findings suggest that when people's experience of outcome and procedural fairness diverged from how they expected to be treated, they reacted in the direction of their experiences; otherwise, their reactions were consistent with their expectations.

**EXCLUDE (Within and between-group competition is not really applicable to CCCs. The methodology of using an investment game is also not applicable - 4)** 18: Liu G, Lin C, Xin Z. The effects of within- and between-group competition on trust and trustworthiness among acquaintances. PLoS One. 2014 Jul 18;9(7):e103074. doi: 10.1371/journal.pone.0103074. eCollection 2014. PubMed

PMID: 25036147; PubMed Central PMCID: PMC4103862.

### Abstract

Several studies have indicated that between-group competition is a key stimulator of trust and trustworthiness. Another important but neglected type of competition may also affect trust and trustworthiness: within-group competition, especially competition among acquaintances. The present study investigated the effects of both within- and between-group competition on trust and trustworthiness, which were measured using an investment game played by acquaintances. We found that, compared to the participants' performance in the non-competition condition, when individuals were motivated to compete with their in-group members or the other groups for financial rewards, they demonstrated more trust. When individuals were motivated to compete with their in-group members, they exhibited lower trustworthiness than in non-competition and between-group competition. In addition, within-group competition decreased the trustor's payoff while both within- and between- group competition increased the trustee's payoff. Finally, we found that males trusted their group members more than females.

**EXCLUDE (This study seems to really focus on the relationship of trust between a patient and his/her treating physician, not about a group of physicians/medical educators making decisions - 4)** 21: Rolfe A, Cash-Gibson L, Car J, Sheikh A, McKinstry B. Interventions for improving patients' trust in doctors and groups of doctors. Cochrane Database Syst Rev. 2014 Mar 4;(3):CD004134. doi: 10.1002/14651858.CD004134.pub3. Review. PubMed PMID: 24590693.

### Abstract

#### BACKGROUND:

Trust is a fundamental component of the patient-doctor relationship and is associated with increased satisfaction, adherence to treatment, and continuity of care. Our 2006 review found little evidence that interventions improve patients' trust in their doctor; therefore an updated search was required to find out if there is further evidence of the effects of interventions that may improve trust in doctors or groups of doctors.

#### OBJECTIVES:

To update our earlier review assessing the effects of interventions intended to improve patients' trust in doctors or a group of doctors.

#### SEARCH METHODS:

In 2003 we searched the Cochrane Central Register of Controlled Trials (CENTRAL, The Cochrane Library), MEDLINE, EMBASE, Health Star, PsycINFO, CINAHL, LILACS, African Trials Register, African Health Anthology, Dissertation Abstracts International and the bibliographies of studies selected for inclusion. We also contacted researchers active in the field. We updated and re-ran the searches on available original databases (Cochrane Central Register of Controlled Trials (CENTRAL, The Cochrane Library issue 2, 2013), MEDLINE (OvidSP), EMBASE (OvidSP), PsycINFO (OvidSP), CINAHL (Ebsco)) as well as Proquest Dissertations and Current Contents for the period 2003 to 18 March 2013.

#### SELECTION CRITERIA:

Randomised controlled trials (RCTs), quasi-randomised controlled trials, controlled before and after studies, and interrupted time series of interventions (informative, educational, behavioural, organisational) directed at doctors or patients (or carers) where trust was assessed as a primary or secondary outcome.

#### DATA COLLECTION AND ANALYSIS:

Two review authors independently extracted data and assessed the risk of bias of included studies. Where mentioned, we extracted data on adverse effects. We synthesised data narratively.

#### MAIN RESULTS:

We included 10 randomised controlled trials (including 7 new trials) involving 11,063 patients. These studies were all undertaken in North America, and all but two involved primary care. As expected, there was considerable heterogeneity between the studies. Interventions were of three main types; three employed additional physician training, four were education for patients and three provided additional information about doctors in terms of financial incentives or consulting style. Additionally, several different measures of trust were employed.The studies gave conflicting results. Trials showing a small but statistically-significant increase in trust included: a trial of physician disclosure of financial incentives; a trial of providing choice of physician based on concordance between patient and physician beliefs about care; a trial of group visits for new inductees into a Health Maintenance Organisation; a trial of training oncologists in communication skills; and a trial of group visits for diabetic patients. However, trust was not affected in a subsequent larger trial of group visits for uninsured people with diabetes, nor with a decision aid for helping choose statins, another trial of disclosure of financial incentives or specifically training doctors to increase trust or cultural competence. There was no evidence of harm from any of the studies.

#### AUTHORS' CONCLUSIONS:

Overall, there remains insufficient evidence to conclude that any intervention may increase or decrease trust in doctors. This may be due in part to the sensitivity of trust instruments, and a ceiling effect, as trust in doctors is generally high. It may be that current measures of trust are insufficiently sensitive. Further trials are required to explore the impact of doctors' specific training or the use of a patient-centred or decision-sharing approach on patients' trust, especially in the areas of healthcare provider choice, and induction into healthcare organisation. International trials would be of particular benefit. The review was constrained by the lack of consistency between trust measurements, timeframes and populations.

**INCLUDE IN ARTICLE REVIEW (coded)** 29: Terwel BW, Harinck F, Ellemers N, Daamen DD. Voice in political decision-making: the effect of group voice on perceived trustworthiness of

decision makers and subsequent acceptance of decisions. J Exp Psychol Appl. 2010

Jun;16(2):173-86. doi: 10.1037/a0019977. PubMed PMID: 20565202.

### Abstract

The implementation of carbon dioxide capture and storage technology (CCS) is considered an important climate change mitigation strategy, but the viability of this technology will depend on public acceptance of CCS policy decisions. The results of three experiments with students as participants show that whether or not interest groups receive an opportunity to express their opinions in the decision-making process (i.e., group voice) affects acceptance of CCS policy decisions, with inferred trustworthiness of the decision maker mediating this effect. Decision-making procedures providing different interest groups with equal opportunities to voice their opinions instigate more trust in the decision maker and, in turn, lead to greater willingness to accept decisions compared to no-voice procedures (i.e., unilateral decision-making-Study 1) and unequal group-voice procedures (i.e., when one type of interest group receives voice, but another type of interest group does not-Study 2). Study 3 further shows that an individual's own level of knowledge about CCS moderates the desire for an opportunity for members of the general public to voice opinions in the decision-making process, inferred trustworthiness of decision makers, and policy acceptance. These results imply that people care about voice in decision-making even when they are not directly personally involved in the decision-making process. We conclude that people tend to use procedural information when deciding to accept or oppose policy decisions on political complex issues; hence, it is important that policymakers use fair group-voice procedures and that they communicate to the public how they arrive at their decisions.

**EXCLUDE (The population studied, the focus on politics/leadership, and the models reviewed don’t really apply to CCCs - 3)** 31: Wright BW. Power, trust, and Science of Unitary Human Beings influence political leadership: a celebration of Barrett's power theory. Nurs Sci Q. 2010 Jan;23(1):60-2. doi: 10.1177/0894318409353794. Review. PubMed PMID: 20026730.

### Abstract

The importance of nurses' participation in health policy leadership is discussed within the context of Rogers' science of unitary human beings, Barrett's power theory, and one nurse-politician's experience. Nurses have a major role to play in resolving public policy issues that influence the health of people. A brief review of the history of nurses in the political arena is presented. Research related to power and trust is reviewed. Suggested strategies for success in political situations are offered.

**EXCLUDE (Different leader types in the setting of step-level public good dilemmas and the population studied does not really apply to CCCs - 3)** 37: De Cremer D. Which type of leader do I support in step-level public good dilemmas? The roles of level of threshold and trust. Scand J Psychol. 2007 Feb;48(1):51-9. PubMed PMID: 17257369.

### **Abstract**

The present research examined the moderating effect of the level of threshold on people's preferences for different leader types in step-level public good dilemmas. It was assumed that the primary focus of people in step-level public good dilemmas is to make sure that the group surpasses the threshold. Consequently, when the level of threshold is difficult to reach people are expected to provide more support for and cooperate with a leader that monitors and controls the contributions made toward the public good. However, if the threshold is easy to surpass people will focus more on whether the obtained public good or bonus will be distributed according to agreements, suggesting that people will provide more support to and cooperate with a leader that monitors and controls the distribution of the bonus. These predictions were confirmed across two experiments using a step-level public good paradigm with a dichotomous (Study 1) and a continuous (Study 2) contribution choice. Moreover, the results also revealed that perceptions of trust accounted, in part, for the effect of level of threshold on people's leadership preferences.

**EXCLUDE (This study is a precursor to the Cochrane review above it. Excluding this for similar reasons - 4)** 38: McKinstry B, Ashcroft RE, Car J, Freeman GK, Sheikh A. Interventions for improving patients' trust in doctors and groups of doctors. Cochrane Database Syst Rev. 2006 Jul 19;(3):CD004134. Review. Update in: Cochrane Database Syst

Rev. 2014;3:CD004134. PubMed PMID: 16856033.

### **Abstract**

#### **BACKGROUND:**

Trust is a fundamental component of the patient-doctor relationship and is associated with increased satisfaction, adherence to treatment, and continuity of care. It is not clear if there are interventions known to be effective in enhancing patient trust in doctors.

#### **OBJECTIVES:**

To assess the effects of interventions intended to improve a patient's trust in the doctor or a group of doctors.

#### **SEARCH STRATEGY:**

We searched the Cochrane Central Register of Controlled Trials (The Cochrane Library Issue 1 2003), MEDLINE(1966 to week 4 2003), EMBASE (1985 to July 2003), Health Star (1975 to July 2004), PsycINFO (1967 to July 2004), CINAHL (1982 to June 2003), LILACS (1982 to April 2003), African Trials Register (1948 to April 2003), African Health Anthology (1924 to April 2003), Dissertation Abstracts International (1861 to April 2003) and the bibliographies of studies assessed for inclusion. We also searched the bibliographies of studies assessed for inclusion, and contacted researchers active in the field.

#### **SELECTION CRITERIA:**

Randomised controlled trials (RCTs), controlled clinical trials, controlled before and after studies, and interrupted time series studies of interventions (informative, educational, behavioural, organisational) directed at doctors or patients (or carers) where trust was assessed as a primary or secondary outcome.

#### **DATA COLLECTION AND ANALYSIS:**

Two review authors independently assessed trial quality and extracted data.

#### **MAIN RESULTS:**

Three RCTs, all published in English and set in North American primary care, and involving 1916 participants, were included. There was considerable heterogeneity in terms of aims, format and content of the interventions. One trial of a training intervention for family doctors to improve communication behaviours (20 doctors assessed by 414 patients) showed no effect on trust. The other two interventions were patient focussed. One explored the impact on trust of disclosing physician incentives to patients (n= 918) in a Health Maintenance Organisation (HMO) and showed no diminution in trust. Another investigated the effect of induction visits on new HMO members' (n=564) trust in their HMO doctors. Trust in doctors rose compared with control following the visit for one type of induction visit, the group visit (Trust out of 10 (standard deviation (SD)) was 8.8 (1.5) and 7.1 (2.2), difference 1.7, (95% confidence interval 1.22 to 2.18)). However there were many drop-outs and analysis was not on intention to treat.

#### **AUTHORS' CONCLUSIONS:**

Overall there remains insufficient evidence to conclude that any intervention may increase or decrease trust in doctors. Further trials are required to explore the impact of policy changes, guidelines and specific doctors' training on patients' trust.

**EXCLUDE (Based upon the title alone, this doesn’t seem applicable to CCCs. Also there is not abstract, and I don’t feel pulling the full text is warranted for this study - 7)** 46: Collopy BJ. The moral underpinning of the proxy-provider relationship: issues of trust and distrust. J Law Med Ethics. 1999 Spring;27(1):37-45. PubMed PMID: 11657141.

No abstract available

**EXCLUDE (This article seems to be really focused on QI, not group decision-making. I don’t feel this is applicable to CCCs - 3)** 49: Klazinga N. Re-engineering trust: the adoption and adaption of four models for external quality assurance of health care services in western European health care systems. Int J Qual Health Care. 2000 Jun;12(3):183-9. Review. PubMed PMID: 10894189.

### Abstract

Accreditation, ISO, EFQLM and visitatie are, in essence, control mechanisms in health care systems. An analysis is provided of the way the four models have been adopted and adapted in European health care systems over the past decade. After a short discussion of the major reforms in the European health care systems in the direction of regulated markets, deregulation and decentralization, the features of the four models are highlighted and it is explained how each of them can help to fill the 'accountability gap' between health care providers on the one hand and patients, financiers and governments on the other. The quality system perspective of ISO, the quality management development perspective of EFQM, the health care organization perspective of accreditation and the professional perspective of visitatie can each be appropriate given the balance of power between parties in the health care system and the focus and scope of accountability. Although a general convergence between the four models can be observed, actual convergence will depend on their adoption in specific health system contexts. Potential pitfalls for further convergence are the differences in distribution of responsibilities for quality of care among the various European countries, the drift away from clinical decision making, bureaucratic tendencies and too much focus on efficiency and patient empowerment compared with attention to medical effectiveness.

**INCLUDE IN ARTICLE REVIEW (coded)** 51: Simons TL, Peterson RS. Task conflict and relationship conflict in top management teams: the pivotal role of intragroup trust. J Appl Psychol. 2000 Feb;85(1):102-11. PubMed PMID: 10740960.

### **Abstract**

Task conflict is usually associated with effective decisions, and relationship conflict is associated with poor decisions. The 2 conflict types are typically correlated in ongoing groups, however, which creates a prescriptive dilemma. Three explanations might account for this relationship--misattribution of task conflict as relationship conflict, harsh task conflict tactics triggering relationship conflict, and misattribution of relationship conflict as task conflict. The authors found that intragroup trust moderates the relationship between task conflict and relationship conflict in 70 top management teams. This result supports the "misattribution of task conflict" explanation. The authors also found a weak effect that is consistent with the argument that tactical choices drive the association between the 2 conflict types. We infer that trust is a key to gaining the benefits of task conflict without suffering the costs of relationship conflict.

**EXCLUDE (This study focuses on patient and treating provider trust, similar to the Cochrane reviews. This is also a negative study. I don’t feel this will be directly applicable to CCCs - 3)** 53: Thom DH, Bloch DA, Segal ES. An intervention to increase patients' trust in their physicians. Stanford Trust Study Physician Group. Acad Med. 1999 Feb;74(2):195-8. PubMed PMID: 10065061.

### Abstract

#### PURPOSE:

To investigate the effect of a one-day workshop in which physicians were taught trust-building behaviors on their patients' levels of trust and on outcomes of care.

#### METHOD:

In 1994, the study recruited 20 community-based family physicians and enrolled 412 consecutive adult patients from those physicians' practices. Ten of the physicians (the intervention group) were randomly assigned to receive a one-day training course in building and maintaining patients' trust. Outcomes were patients' trust in their physicians, patients' and physicians' satisfaction with the office visit, continuity in the patient-physician relationship, patients' adherence to their treatment plans, and the numbers of diagnostic tests and referrals.

#### RESULTS:

Physicians and patients in the intervention and control groups were similar in demographic and other data. There was no significant difference in any outcome. Although their overall ratings were not statistically significantly different, the patients of physicians in the intervention group reported more positive physician behaviors than did the patients of physicians in the control group.

#### CONCLUSIONS:

The trust-building workshop had no measurable effect on patients' trust or on outcomes hypothesized to be related to trust.

**EXCLUDE (these articles have already been excluded based upon the title review):**

**EXCLUDE (politics; not applicable or readily transferable to CCCs - 3):** 1: Balliet D, Tybur JM, Wu J, Antonellis C, Van Lange PAM. Political Ideology,

Trust, and Cooperation: In-group Favoritism among Republicans and Democrats

during a US National Election. J Conflict Resolut. 2018 Apr;62(4):797-818. doi:

10.1177/0022002716658694. Epub 2016 Jul 21. PubMed PMID: 29593363; PubMed Central

PMCID: PMC5858642.

**EXCLUDE (acronym; not applicable or readily transferable to CCCs - 1):** 2: Shaughnessy AF, Vaswani A, Andrews BK, Erlich DR, D'Amico F, Lexchin J,

Cosgrove L. Developing a Clinician Friendly Tool to Identify Useful Clinical

Practice Guidelines: G-TRUST. Ann Fam Med. 2017 Sep;15(5):413-418. doi:

10.1370/afm.2119. PubMed PMID: 28893810; PubMed Central PMCID: PMC5593723.

**EXCLUDE (child development; not readily applicable or transferable to CCCs - 3):** 3: Grütter J, Gasser L, Zuffianò A, Meyer B. Promoting Inclusion Via Cross-Group

Friendship: The Mediating Role of Change in Trust and Sympathy. Child Dev. 2017

Jun 19. doi: 10.1111/cdev.12883. [Epub ahead of print] PubMed PMID: 28626994.

**EXCLUDE (hormones/biochemistry; not applicable or readily transferable to CCCs - 1):** 4: Kret ME, De Dreu CK. Pupil-mimicry conditions trust in partners: moderation by

oxytocin and group membership. Proc Biol Sci. 2017 Mar 15;284(1850). pii:

20162554. doi: 10.1098/rspb.2016.2554. PubMed PMID: 28250181; PubMed Central

PMCID: PMC5360920.

**EXCLUDE (subject/population studied; not applicable or readily transferable to CCCs - 2):** 6: Ohmann K, Stahl J, Mussweiler T, Kedia G. Immediate relativity: EEG reveals

early engagement of comparison in social information processing. J Exp Psychol

Gen. 2016 Nov;145(11):1512-1529. PubMed PMID: 27797558.

**EXCLUDE (substance abuse; not applicable or readily transferable to CCCs - 3):** 7: Silverman MJ. Effects of Live and Educational Music Therapy on Working

Alliance and Trust With Patients on Detoxification Unit: A Four-Group

Cluster-Randomized Trial. Subst Use Misuse. 2016 Nov 9;51(13):1741-50. doi:

10.1080/10826084.2016.1197263. Epub 2016 Aug 3. PubMed PMID: 27487408.

**EXCLUDE (politics; not applicable or readily transferable to CCCs - 3):** 8: Born M, Akkerman A, Thommes K. Peer influence on protest participation:

Communication and trust between co-workers as inhibitors or facilitators of

mobilization. Soc Sci Res. 2016 Mar;56:58-72. doi:

10.1016/j.ssresearch.2015.11.003. Epub 2015 Nov 10. PubMed PMID: 26857172.

**EXCLUDE (ethics; not applicable or readily transferable to CCCs - 3):** 9: Courtwright A, Rubin E. Who should Decide for the Unrepresented? Bioethics.

2016 Mar;30(3):173-80. doi: 10.1111/bioe.12185. Epub 2015 Aug 26. PubMed PMID:

26307414.

**EXCLUDE (population studied; not applicable or readily transferable to CCCs - 2):** 10: Merrick ET, Fry M, Duffield C, Stasa H. Trust and decision-making: How nurses in Australian general practice negotiate role limitations. Collegian.

2015;22(2):225-32. PubMed PMID: 26281411.

**EXCLUDE (subject/population studied; not applicable or readily transferable to CCCs - 3):** 11: Ye J, Ng SH. An intermediary enhances out-group trust and in-group profit expectation of Chinese but not Australians. Int J Psychol. 2017

Jun;52(3):189-196. doi: 10.1002/ijop.12199. Epub 2015 Jul 27. PubMed PMID:

26212598.

**EXCLUDE (population studied; not applicable or readily transferable to CCCs - 3):** 13: Lossie AC, Green J. Building Trust: The History and Ongoing Relationships

Amongst DSD Clinicians, Researchers, and Patient Advocacy Groups. Horm Metab Res.

2015 May;47(5):344-50. doi: 10.1055/s-0035-1548793. Epub 2015 Apr 13. Review.

PubMed PMID: 25868122.

**EXCLUDE (child psychology; not readily transferable to CCCs - 4):** 16: Elashi FB, Mills CM. Do children trust based on group membership or prior

accuracy? The role of novel group membership in children's trust decisions. J Exp

Child Psychol. 2014 Dec;128:88-104. doi: 10.1016/j.jecp.2014.07.003. Epub 2014

Aug 11. PubMed PMID: 25108696.

**EXCLUDE (substance abuse; not applicable or readily transferable to CCCs - 3):** 17: Silverman MJ. Effects of a live educational music therapy intervention on

acute psychiatric inpatients' perceived social support and trust in the

therapist: a four-group randomized effectiveness study. J Music Ther. 2014

Fall;51(3):228-49. doi: 10.1093/jmt/thu011. Epub 2014 Jul 23. PubMed PMID:

25057140.

**EXCLUDE (population studied; not applicable or readily transferable to CCCs - 1):** 19: Woodroffe R. Building trust on bovine TB. Vet Rec. 2014 Mar 8;174(10):254-5.

doi: 10.1136/vr.f1811. PubMed PMID: 24736823.

**EXCLUDE (population studied; not applicable or readily transferable to CCCs - 1):** 20: Jones TO. Building trust on TB. Vet Rec. 2014 Mar 22;174(12):307-8. doi:

10.1136/vr.g2232. PubMed PMID: 24652849.

**EXCLUDE (not applicable or readily transferable to CCCs, deception not really the focus of this review - 3):** 22: Slessor G, Phillips LH, Ruffman T, Bailey PE, Insch P. Exploring own-age

biases in deception detection. Cogn Emot. 2014 Apr;28(3):493-506. doi:

10.1080/02699931.2013.839438. Epub 2013 Nov 28. PubMed PMID: 24283379.

**EXCLUDE (population studied; not applicable or readily transferable to CCCs - 3):** 23: Ghobadifar MA. Importance of patient-physician relationship in cancer

prevention: a self experience-based survey. Asian Pac J Cancer Prev.

2013;14(8):4943-4. PubMed PMID: 24083773.

**EXCLUDE (Trust not used in the right context; not applicable or readily transferable to CCCs - 1):** 24: Ocloo J, O'Shea A, Fulop N. Empowerment or rhetoric? Investigating the role

of NHS Foundation Trust governors in the governance of patient safety. Health

Policy. 2013 Aug;111(3):301-10. doi: 10.1016/j.healthpol.2013.05.005. Epub 2013

Jun 12. PubMed PMID: 23764151.

**EXCLUDE (population studied; not applicable or readily transferable to CCCs - 3):** 25: Pivetti M, Montali L, Simonetti G. The discourse around usefulness, morality, risk and trust: a focus group study on prenatal genetic testing. Prenat Diagn. 2012 Dec;32(12):1205-11. doi: 10.1002/pd.3990. Epub 2012 Oct 24. PubMed PMID:23097220.

**EXCLUDE (child development; not readily transferable to CCCs - 2):** 26: Chen EE, Corriveau KH, Harris PL. Children trust a consensus composed of

outgroup members--but do not retain that trust. Child Dev. 2013

Jan-Feb;84(1):269-82. doi: 10.1111/j.1467-8624.2012.01850.x. Epub 2012 Sep 19.

PubMed PMID: 22994587.

**EXCLUDE (race/reputation not the focus of our review; not applicable or readily transferable to CCCs - 3):** 27: Stanley DA, Sokol-Hessner P, Fareri DS, Perino MT, Delgado MR, Banaji MR, Phelps EA. Race and reputation: perceived racial group trustworthiness influences the neural correlates of trust decisions. Philos Trans R Soc Lond B Biol Sci. 2012 Mar 5;367(1589):744-53. doi: 10.1098/rstb.2011.0300. PubMed PMID: 22271789;

PubMed Central PMCID: PMC3260848.

**EXCLUDE (population studied; not applicable or readily transferable to CCCs - 3):** 28: Davies L, Rhodes LA, Grossman DC, Rosenberg MC, Stevens DP. Decision making in head and neck cancer care. Laryngoscope. 2010 Dec;120(12):2434-45. doi:

10.1002/lary.21036. PubMed PMID: 21089143.

**EXCLUDE (population studied; not applicable or readily transferable to CCCs - 3):** 30: Shelton RC, Winkel G, Davis SN, Roberts N, Valdimarsdottir H, Hall SJ, Thompson HS. Validation of the group-based medical mistrust scale among urban black men. J Gen Intern Med. 2010 Jun;25(6):549-55. Doi: 10.1007/s11606-010-1288-y. Epub 2010 Mar 2. PubMed PMID: 20195782; PubMed Central PMCID: PMC2869405.

**EXCLUDE (population studied; not applicable or readily transferable to CCCs - 3):** 32: Brodsky SL, Neal TM, Cramer RJ, Ziemke MH. Credibility in the courtroom: how likeable should an expert witness be? J Am Acad Psychiatry Law.

2009;37(4):525-32. PubMed PMID: 20019000.

**EXCLUDE (ethics and end of life care?; not applicable or readily transferable to CCCs - 3):** 33: Howe EG. Increasing consensus with patients and their loved ones. J Clin

Ethics. 2009 Spring;20(1):3-12. PubMed PMID: 19385318.

**EXCLUDE (religion; not applicable or readily transferable to CCCs - 3):** 34: Magyar-Russell G, Fosarelli P, Taylor H, Finkelstein D. Ophthalmology

patients' religious and spiritual beliefs: an opportunity to build trust in the

patient-physician relationship. Arch Ophthalmol. 2008 Sep;126(9):1262-5. doi:

10.1001/archopht.126.9.1262. PubMed PMID: 18779488.

**EXCLUDE (surrogate decision makers; not applicable or readily transferable to CCCs - 3):** 35: Zier LS, Burack JH, Micco G, Chipman AK, Frank JA, Luce JM, White DB. Doubt and belief in physicians' ability to prognosticate during critical illness: the

perspective of surrogate decision makers. Crit Care Med. 2008 Aug;36(8):2341-7.

doi: 10.1097/CCM.0b013e318180ddf9. PubMed PMID: 18596630; PubMed Central PMCID:

PMC2628287.

**EXCLUDE (PHR?, medical record?; not applicable or readily transferable to CCCs - 3):** 36: Rhodes HB. The PHR quandary. Despite the benefits, issues of technology and

trust slow adoption. J AHIMA. 2007 Apr;78(4):66-7, 69. PubMed PMID: 17455850.

**EXCLUDE (ethics; not applicable or readily transferable to CCCs - 3):** 39: Loewy EH. In defense of paternalism. Theor Med Bioeth. 2005;26(6):445-68.

PubMed PMID: 16292603.

**EXCLUDE (politics; not applicable or readily transferable to CCCs - 3):** 40: Clark CC. Civil disobedience: the devil is in the details. Hastings Cent Rep.

2005 Jul-Aug;35(4):5; author reply 5-6. PubMed PMID: 16225291.

**EXCLUDE: (population studied; not applicable or readily transferable to CCCs - 1):** 41: Walsgrove H, Fulbrook P. Advancing the clinical perspective: a practice

development project to develop the nurse practitioner role in an acute hospital

trust. J Clin Nurs. 2005 Apr;14(4):444-55. PubMed PMID: 15807751.

**EXCLUDE (population studied; not applicable or readily transferable to CCCs - 1):** 42: Cannell P. Linking clinical audit in general dental services to primary care

trust clinical governance -- progress report of an approach used in Southend.

Prim Dent Care. 2005 Jan;12(1):23-6. PubMed PMID: 15703157.

**EXCLUDE (population studied; not applicable or readily transferable to CCCs - 2):** 43: Batiuk ME, Boland JA, Wilcox N. Project Trust: breaking down barriers between

middle school children. Adolescence. 2004 Fall;39(155):531-8. PubMed PMID:

15673228.

**EXCLUDE (ethics; not applicable or readily transferable to CCCs - 3):** 44: Pilkington FB. Exploring ethical implications for acting faithfully in

professional relationships. Nurs Sci Q. 2004 Jan;17(1):27-32. Review. PubMed

PMID: 14752949.

**EXCLUDE (“trust” is used in a different context here than the focus of our review - 1):** 45: Doyal L, Colvin B. The Clinical Ethics Committee at Barts and the London NHS Trust: rationale, achievements, and difficulties. HEC Forum. 2002

Mar;14(1):26-36. PubMed PMID: 12001799.

**EXCLUDE (population studied; not applicable or readily transferable to CCCs - 3):** 47: Tyler TR. Public trust and confidence in legal authorities: What do majority and minority group members want from the law and legal institutions? Behav Sci Law. 2001;19(2):215-35. PubMed PMID: 11385699.

**EXCLUDE (“trust” is used in a different context here than the focus of our review - 1):** 48: Dennis SM, Sharp SJ, Vickers MR, Frost CD, Crompton GK, Barnes PJ, Lee TH. Regular inhaled salbutamol and asthma control: the TRUST randomised trial.

Therapy Working Group of the National Asthma Task Force and the MRC General

Practice Research Framework. Lancet. 2000 May 13;355(9216):1675-9. PubMed PMID:

10905242.

**EXCLUDE (based upon the journal, this is likely a commentary or editorial; not applicable or readily transferable to CCCs - 6):** 50: Skeptical reaction to UnitedHealthcare's "we trust doctors" gambit. Med Econ.

2000 Jan 24;77(2):30. PubMed PMID: 10787877.

**EXCLUDE (ethics; not applicable or readily transferable to CCCs - 3):** 52: Howe EG. Ethics consultants: could they do better? J Clin Ethics. 1999

Spring;10(1):13-25. Review. PubMed PMID: 10394534.

**EXCLUDE (race/politics; not applicable or readily transferable to CCCs - 4):** 54: Ferguson JA, Weinberger M, Westmoreland GR, Mamlin LA, Segar DS, Greene JY, Martin DK, Tierney WM. Racial disparity in cardiac decision making: results from

patient focus groups. Arch Intern Med. 1998 Jul 13;158(13):1450-3. PubMed PMID:

9665355.

**EXCLUDE (race/politics; not applicable or readily transferable to CCCs - 3):** 55: Berkowitz S. Race and the delivery of care. Hastings Cent Rep. 1998

Jan-Feb;28(1):5; author reply 6. PubMed PMID: 9539037.

**EXCLUDE (“trust” is used in a different context here than the focus of our review - 1):** 56: Fins JJ. A medical trust fund for managed care: the legacy of Hughley vs

Rocky Mountain Health Care Maintenance Organization. J Am Geriatr Soc. 1998

Mar;46(3):365-8. PubMed PMID: 9514388.

**EXCLUDE (race/politics, end of life care; not applicable or readily transferable to CCCs - 3):** 57: Krakauer EL, Truog RD. Mistrust, racism, and end-of-life treatment. Hastings

Cent Rep. 1997 May-Jun;27(3):23; discussion 23-5. PubMed PMID: 9219020.

**EXCLUDE (race/politics, end of life care; not applicable or readily transferable to CCCs - 4):** 58: Hauser JM, Kleefield SF, Brennan TA, Fischbach RL. Minority populations and

advance directives: insights from a focus group methodology. Camb Q Healthc

Ethics. 1997 Winter;6(1):58-71. PubMed PMID: 9111963.

**EXCLUDE (end of life care?/surrogate decision-making, not applicable or readily transferable to CCCs - 3):** 59: Povar GJ. Second guessing the patient's trust: facing the challenge of the difficult surrogate. J Clin Ethics. 1993 Summer;4(2):168-71. PubMed PMID: 8334281.

**EXCLUDE (population studied; not applicable or readily transferable to CCCs - 1):** 60: Rie MA. Practicing medicine, fiduciary trust privacy, and public moral

interloping after Cruzan. J Med Philos. 1992 Dec;17(6):647-64. PubMed PMID:

1479310.

**EXCLUDE (population studied; not applicable or readily transferable to CCCs - 1):** 61: Kerr R. Group health: avoiding a MET (multiple-employer trust) crisis. Assoc

Manage. 1989 Apr;41(4):82-5. PubMed PMID: 10292898.
