# Supplementary material for: Trust in Group Decisions: a scoping review
Source: BMC Med Educ. 2019 Aug 14;19:309. doi: 10.1186/s12909-019-1726-4 (PMC6693175; doi:10.1186/s12909-019-1726-4)
Supplement: Supplementary file 6 — SCOPUS Search Publication Review. (DOCX 87 kb) [file 12909_2019_1726_MOESM6_ESM.docx]

**Additional 6: SCOPUS, 9 April 2018 (titles), 16 April 2018 (abstracts)**

**Search strategy:** (TITLE(trust OR trustworth*) AND TITLE(group OR groups OR team OR teams OR committee OR committees OR jury OR juries) AND TITLE-ABS-KEY(decision OR decisions OR success OR successes OR outcome OR outcomes)): **214 Results**

**Limit**: “English (language, 209),” “article (134)” (including “article in press (5)”) and “review(12)” (excludes “conference paper(52),” “book chapter(8),” “note(2),” “letter(1),” “short survey(1)”): **146 Results, 73 “exclude,” 73 “include in abstract review”**

**16 April 2018: 73 “include in abstract review,” 47 “exclude,” 26 “include in article review”**

**22 June 2018: 1 additional “exclude,” 25 “include in article review”**

**5 July 2018: 6 “waiting to code - Virtual Teams,” 19 “include in article review”**

**ABSTRACT REVIEW (73 articles):**

**INCLUDE IN ARTICLE REVIEW (reviewed, used to build extraction sheet)** Costa, A.C., Fulmer, C.A., Anderson, N.R.
Trust in work teams: An integrative review, multilevel model, and future directions
(2018) Journal of Organizational Behavior, 39 (2), pp. 169-184.
https://www.scopus.com/inward/record.uri?eid=2-s2.0-85021952242&doi=10.1002%2fjob.2213&partnerID=40&md5=9e539e00215e8922e9be481eae093373
DOCUMENT TYPE: Review
SOURCE: Scopus

This article presents an integrative review of the rapidly growing body of research on trust in work teams. We start by analyzing prominent definitions of trust and their theoretical foundations, followed by different conceptualizations of trust in teams emphasizing its multilevel, dynamic, and emergent nature. We then review the empirical research and its underlying theoretical perspectives concerning the emergence and development of trust in teams. On the basis of this review, we propose an integrated conceptual framework that organizes the field and can advance knowledge of the multilevel nature of trust in teams. Our conclusion is that trust in teams resides at multiple levels of analysis simultaneously, is subject to factors across levels in organizations, and impacts performance and other relevant outcomes both at the individual and team levels. We argue that research should not only differentiate between interpersonal trust between members from collective trust at the team level but also emphasize the interplay within and between these levels by considering cross-level influences and dynamics. We conclude by proposing 4 major directions for future research and 3 critical methodological recommendations for study designs derived from our review and framework.

**EXCLUDE (New venture teams/emergence not really applicable to CCCs - 2)** Williams Middleton, K., Nowell, P.
Team trust and control in new venture emergence
(2018) International Journal of Entrepreneurial Behaviour and Research, . Article in Press.
https://www.scopus.com/inward/record.uri?eid=2-s2.0-85040742125&doi=10.1108%2fIJEBR-01-2017-0048&partnerID=40&md5=63487a4e1a29ec47dabc10e39247aeb8
DOCUMENT TYPE: Article in Press
SOURCE: Scopus

Effective internal dynamics of new venture teams is seen as a key contributor to venture success. The purpose of this paper is to investigate the ways in which new venture teams consisting of nascent entrepreneurs initiate trust and control during venture emergence. Design/methodology/approach – Dimensions of trust and control are developed into an analytical framework applied to documented team norms. Coding detects frequency of trust and control dimensions. Supplementary data triangulate findings and explore follow-on effects in team dynamics and venture emergence. Findings – Frequency of coded dimensions generates a venture team profile. Teams prime their dynamics through use of trust and/or control language in documented norms. Priming is seen to influence entrepreneurial perseverance during venture emergence, stemming either directly from team dynamics, or indirectly from key shareholder relationships or environmental conditions. Research limitations/implications – Data are bounded to a specific contextual setting representing incubation and education, where the nascent entrepreneurs are simultaneously students. The complexity of venture emergence means that multiple factors influencing new venture teams may influence trust and control in ways currently unaccounted for. Practical implications – Exploration of trust and control during venture emergence emphasizes soft-skills critical to entrepreneurial perseverance and venture success. Team norms can be designed to prime toward trust or control, and can be indicative of teams' sensitivity to external factors, enabling evidence for intervention. Originality/value – The paper illustrates ways in which trust and control influence team dynamics during venture emergence.

**INCLUDE IN ARTICLE REVIEW (EXCLUDE, after full text review, this article focuses on initial facial trustworthiness scores in the absence of other information about a group, unlikely to be helpful for CCCs, 22 June 2018 - 2)** Kong, D.T.
Trust toward a group of strangers as a function of stereotype-based social identification
(2018) Personality and Individual Differences, 120, pp. 265-270.
https://www.scopus.com/inward/record.uri?eid=2-s2.0-85015418798&doi=10.1016%2fj.paid.2017.03.031&partnerID=40&md5=d35de8f1471f72953d69a383af895263
DOCUMENT TYPE: Article
SOURCE: Scopus

Individuals often have to decide whether to trust others at zero or little acquaintance. In this instance, individuals rely on social stereotypes as a guide to their trust decisions. Yet little is known regarding how social cues lead to trust. The present research examines the stereotype-based social identification mechanism through which the facial trustworthiness of a group of strangers determines an individual's trust toward the group. Two experimental studies showed that group warmth (rather than competence or dominance) stereotypes and subsequent self-group merging (inclusion of the group in the self-representation) fully mediated the effect of group facial trustworthiness on trust toward the group at zero acquaintance, whether the level of trust was linked with pecuniary rewards or not. The current findings contribute to the emerging literature on trust at zero or little acquaintance.

**EXCLUDE (This study seems largely focused on artificial intelligence and “human-agent teams” which really don’t apply to CCCs - 7)** Schaefer, K.E., Straub, E.R., Chen, J.Y.C., Putney, J., Evans, A.W., III
Communicating intent to develop shared situation awareness and engender trust in human-agent teams
(2017) Cognitive Systems Research, 46, pp. 26-39.
https://www.scopus.com/inward/record.uri?eid=2-s2.0-85014752866&doi=10.1016%2fj.cogsys.2017.02.002&partnerID=40&md5=e6a67c42277d30e23853c340296e717d
DOCUMENT TYPE: Review
SOURCE: Scopus

This paper addresses issues related to integrating autonomy-enabled, intelligent agents into collaborative, human-machine teams. Interaction with intelligent machine agents capable of making independent, goal-directed decisions in human-machine teaming operations constitutes a major change from traditional human-machine interaction involving teleoperation. Communicating the machine agent's intent to human counterparts becomes increasingly important as independent machine decisions become subject to human trust and mental models. The authors present findings from their research that suggest existing user display technologies, tailored with context-specific information and the human's knowledge level of the machine agent's decision process, can mitigate misperceptions of the appropriateness of agent behavioral responses. This is important because misperceptions on the part of human team members increases the likelihood of trust degradation and unnecessary interventions, ultimately leading to disuse of the agent. Examples of possible issues associated with communicating agent intent, as well as potential implications for trust calibration are provided.

**EXCLUDE (Virtual new product teams and Management Control Systems have very little applicability to CCCs - 7)** Bisbe, J., Sivabalan, P.
Management control and trust in virtual settings: A case study of a virtual new product development team
(2017) Management Accounting Research, 37, pp. 12-29.
https://www.scopus.com/inward/record.uri?eid=2-s2.0-85013477716&doi=10.1016%2fj.mar.2017.02.001&partnerID=40&md5=0b6b5f36f9a809e602ab2aea64ff6f4f
DOCUMENT TYPE: Article
SOURCE: Scopus

In this case study, we draw on theory relating to the trust-control nexus to investigate how formal Management Control Systems (MCS) and inter-personal trust relate in Virtual Teams (VTs), and examine the implications of this interplay for VT outcomes. Taking a virtual new product development team as our research site, we evidence the reciprocal influences between trust and formal MCS in a virtual setting. We show that in addition to formal MCS helping uphold inter-personal trust, trust enables the adoption and workability of incomplete formal MCS, hence expanding and shaping the set of control alternatives that are available to a VT. We further extend prior theory by providing evidence of synergies between inter-personal trust and formal MCS that span both the decision-facilitating and decision-influencing MCS roles, indicating that the combination of trust and formal MCS enhances the informational and motivational effects of controls, as well as the motivational effects of trust, on VT outcomes. Overall, this study adds to the accounting literature by shedding light on how formal MCS help manage highly interdependent tasks in dispersed contexts where inter-personal trust is present.

**INCLUDE IN ARTICLE REVIEW (This abstract was reviewed on the ERIC document)** Robertson, M.J.
Trust: the power that binds in team supervision of doctoral students
(2017) Higher Education Research and Development, 36 (7), pp. 1463-1475.
https://www.scopus.com/inward/record.uri?eid=2-s2.0-85030765533&doi=10.1080%2f07294360.2017.1325853&partnerID=40&md5=6d89c2cc609cebcc36d233d7dd434b8e
DOCUMENT TYPE: Article
SOURCE: Scopus

**EXCLUDE (I reviewed this article when I built the framework earlier in this project, and this was a very challenging article to read and understand. In my opinion, it has minimal relevance to CCCs - 4)** van Baalen, S., Carusi, A.
Implicit trust in clinical decision-making by multidisciplinary teams
(2017) Synthese, pp. 1-24. Article in Press.
https://www.scopus.com/inward/record.uri?eid=2-s2.0-85021756800&doi=10.1007%2fs11229-017-1475-z&partnerID=40&md5=8008caa5ac868d0332610aaa57737b27
DOCUMENT TYPE: Article in Press
ACCESS TYPE: Open Access
SOURCE: Scopus

In clinical practice, decision-making is not performed by individual knowers but by an assemblage of people and instruments in which no one member has full access to every piece of evidence. This is due to decision making teams consisting of members with different kinds of expertise, as well as to organisational and time constraints. This raises important questions for the epistemology of medicine, which is inherently social in this kind of setting, and implies epistemic dependence on others. Trust in these contexts is a highly complex social practice, involving different forms of relationships between trust and reasons for trust: based on reasons, and not based on reasons; based on reasons that are easily accessible to reflection and others that are not. In this paper, we focus on what it means to have reasons to trust colleagues in an established clinical team, collectively supporting or carrying out every day clinical decision-making. We show two important points about these reasons, firstly, they are not sought or given in advance of a situation of epistemic dependence, but are established within these situations; secondly they are implicit in the sense of being contained or nested within other actions that are not directly about trusting another person. The processes of establishing these reasons are directly about accomplishing a task, and indirectly about trusting someone else’s expertise or competence. These processes establish a space of reasons within which what it means to have reasons for trust, or not, gains a meaning and traction in these team-work settings. Based on a qualitative study of decision-making in image assisted diagnosis and treatment of a complex disease called pulmonary hypertension (PH), we show how an intersubjective framework, or ‘space of reasons’ is established through team members forging together a common way of identifying and dealing with evidence. In dealing with images as a central diagnostic tool, this also involves a common way of looking at the images, a common mode or style of perception. These frameworks are developed through many iterations of adjusting and calibrating interpretations in relation to those of others, establishing what counts as evidence, and ranking different kinds of evidence. Implicit trust is at work throughout this process. Trusting the expertise of others in clinical decision-making teams occurs while the members of the team are busy on other tasks, most importantly, building up a framework of common modes of seeing, and common ways of identifying and assessing evidence emerge. It is only in this way that trusting or mistrusting becomes meaningful in these contexts, and that a framework for epistemic dependence is established.

**INCLUDE IN ARTICLE REVIEW (waiting to code - Virtual Teams - 7)** Alsharo, M., Gregg, D., Ramirez, R.
Virtual team effectiveness: The role of knowledge sharing and trust
(2017) Information and Management, 54 (4), pp. 479-490.
https://www.scopus.com/inward/record.uri?eid=2-s2.0-85007504980&doi=10.1016%2fj.im.2016.10.005&partnerID=40&md5=e67a007cdbb767b5ab0fa6a4ee76c15a
DOCUMENT TYPE: Article
SOURCE: Scopus

Organizations utilize virtual teams to gather experts who collaborate online to accomplish organizational tasks. The virtual nature of these teams creates challenges to effective collaboration and team outcomes. This research addresses the social effects of knowledge sharing on virtual teams. We propose a conceptual model which hypothesizes a relationship between knowledge sharing, trust, collaboration, and team effectiveness in virtual team settings. The findings suggest that knowledge sharing positively influences trust and collaboration among virtual team members. The findings also suggest that while trust positively influences virtual team collaboration, it does not have a significant direct effect on team effectiveness.

**EXCLUDE (This study seems very algorithmic, focused on more computer-based or AI-based decision making, not really applicable to CCCs - 7)** Liu, Y., Liang, C., Chiclana, F., Wu, J.
A trust induced recommendation mechanism for reaching consensus in group decision making
(2017) Knowledge-Based Systems, 119, pp. 221-231.
https://www.scopus.com/inward/record.uri?eid=2-s2.0-85009445663&doi=10.1016%2fj.knosys.2016.12.014&partnerID=40&md5=b650f9bb11b902a2b7e8a2f3041bda62
DOCUMENT TYPE: Article
SOURCE: Scopus

This article addresses the inconsistency problem in group decision making caused by disparate opinions of multiple experts. To do so, a trust induced recommendation mechanism is investigated to generate personalised advices for the inconsistent experts to reach higher consensus level. The concept of trust degree (TD) is defined to identify the trusted opinion from group experts, and then the visual trust relationship is built to help experts ‘see’ their own trust preferences within the group. Consequently, trust based personalised advices are generated for the inconsistent experts to revisit their opinions. To model the uncertainty of experts, an interval-valued trust decision making space is defined. It includes the novel concepts of interval-valued trust functions, interval-valued trust score (IVTS) and interval-valued knowledge degree (IVKD). The concepts of consensus degree (CD) between an expert and the rest of experts in the group as well as the harmony degree (HD) between the original opinion and the revised opinion are developed for interval-valued trust functions. Combining HD and CD, a more reasonable policy for group consensus is proposed as it should arrive at the threshold value with the maximum value of harmony and consensus degrees simultaneously. Furthermore, because the trust induced recommendation mechanism focuses on changing inconsistent opinions using only opinions from the trusted experts and not from the distrusted ones, the HD based changes cost to reach the threshold value of consensus is lower than previous mechanisms based on the average of the opinion of all experts. Finally, once consensus has been achieved, a ranking order relation for interval-valued trust functions is constructed to select the most appropriate alternative.

**INCLUDE IN ARTICLE REVIEW (This was reviewed on the PUBMED document)** Kweekel, L., Gerrits, T., Rijnders, M., Brown, P.
The Role of Trust in CenteringPregnancy: Building Interpersonal Trust Relationships in Group-Based Prenatal Care in The Netherlands
(2017) Birth, 44 (1), pp. 41-47.
https://www.scopus.com/inward/record.uri?eid=2-s2.0-84998723886&doi=10.1111%2fbirt.12260&partnerID=40&md5=ed96661ffaa3a741780d08d96bec809c
DOCUMENT TYPE: Article
SOURCE: Scopus

**EXCLUDE (This is a book chapter - 7)** Fry, T.N., Nyein, K.P., Wildman, J.L.
Team trust development and maintenance over time
(2017) Research on Managing Groups and Teams, 18, pp. 123-153.
https://www.scopus.com/inward/record.uri?eid=2-s2.0-85026898909&doi=10.1108%2fS1534-085620160000018006&partnerID=40&md5=54b4cbb0ffdbf6a2101bc49b84aee73f
DOCUMENT TYPE: Article
SOURCE: Scopus

Theories of trust imply that team trust develops and grows over time, yet relatively few researchers have taken on the challenge of studying team trust in longitudinal research designs. The purpose of this chapter is to provide a concise summary of the existing literature on team trust over time, and to offer a conceptual model of team-level trust development over time to aid future research on this topic. Methodology/approach - We draw from the Input-Mediator- Output-Input (IMOI) framework, as well as previous multilevel models of organizational trust development, and published findings from longitudinal team trust studies. Findings - Taking a temporal perspective, we consider how team-level mediators and outcomes can both predict and be predicted by team trust trajectories and feedback loops over time, as well as how those relationships with team trust might change based on the existence of other moderating variables including trust violation and repair. Research implications - Future longitudinal team research may use the model as a starting point for investigating the antecedents, as well as the team processes and dynamic emergent states, that can effectively predict trajectories of team trust across various stages of teamwork. Practical implications - Based on our review of extant literature, we provide several recommendations for training and organizational intervention including the importance of management's consideration of team-level trust in providing feedback, enhancing cohesion, and mitigating conflict. Originality/value - We provide insight into the development of team trust trajectories and offer a framework to help guide future longitudinal team trust research.

**INCLUDE IN ARTICLE REVIEW (coded)** La Macchia, S.T., Louis, W.R., Hornsey, M.J., Leonardelli, G.J.
In Small We Trust: Lay Theories About Small and Large Groups
(2016) Personality and Social Psychology Bulletin, 42 (10), pp. 1321-1334.
https://www.scopus.com/inward/record.uri?eid=2-s2.0-84988027179&doi=10.1177%2f0146167216657360&partnerID=40&md5=4ccb3a89b1fba97c14706c2113a489a0
DOCUMENT TYPE: Article
SOURCE: Scopus

Day-to-day interactions often involve individuals interacting with groups, but little is known about the criteria that people use to decide which groups to approach or trust and which to avoid or distrust. Seven studies provide evidence for a “small = trustworthy” heuristic, such that people perceive numerically smaller groups as more benevolent in their character and intentions. As a result of this, individuals in trust-sensitive contexts are more likely to approach and engage with groups that are relatively small than those that are relatively large. We provide evidence for this notion across a range of contexts, including analyses of social categories (Studies 1 and 2), ad hoc collections of individuals (Study 3), interacting panels (Studies 4-6), and generalized, abstract judgments (Study 7). Findings suggest the existence of a general lay theory of group size that may influence how individuals interact with groups.

**EXCLUDE (The location of the study, the type of teams studied, and the focus on knowledge sharing on cross-functional projects limit applicability to CCCs - 7)** Verma, J., Sinha, A.
Knowledge Sharing in Cross-Functional Teams and its Antecedents: Role of Mutual Trust as a Moderator
(2016) Journal of Information and Knowledge Management, 15 (3), art. no. 1650033, .
https://www.scopus.com/inward/record.uri?eid=2-s2.0-84974846269&doi=10.1142%2fS0219649216500337&partnerID=40&md5=0a5ec39081d885f7d6a0020b12025759
DOCUMENT TYPE: Article
SOURCE: Scopus

Growth and success of an organisation are primarily dependent on its ability to develop, leverage, and utilise its knowledge base. This study developed a model to test the impact of antecedents of knowledge Sharing (KS) on team performance. The antecedents were classified as organisational characteristics (structure, learning culture, employee training, reward system, top management support) and individual characteristic (emotional intelligence). The study is based on data collected with the help of structured questionnaires from 582 team members working under 69 teams in 26 surveyed organisations in India. Results suggest that high emotional intelligence increases the extent of KS and hence, has positive impact on team performance among cross-functional team members. Moreover, mutual trust among team members moderates KS behaviour and team performance. Thus, findings confirm the applicability and predictive power of the proposed model. Furthermore, this model contributes to the literature of predicting KS practices in cross-functional projects.

**EXCLUDE (The population studied and how they look at group interaction is not really applicable to CCCs - 4)** Kenworthy, J.B., Voci, A., Ramiah, A.A., Tausch, N., Hughes, J., Hewstone, M.
Building Trust in a Postconflict Society: An Integrative Model of Cross-group Friendship and Intergroup Emotions
(2016) Journal of Conflict Resolution, 60 (6), pp. 1041-1070.
https://www.scopus.com/inward/record.uri?eid=2-s2.0-84981744992&doi=10.1177%2f0022002714564427&partnerID=40&md5=4ac2eaf44a03888b12c803b24b1a7ccb
DOCUMENT TYPE: Article
SOURCE: Scopus

Across one longitudinal and two cross-sectional surveys in Northern Ireland, we tested a model of intergroup relations in which out-group attitudes and behavioral tendencies are predicted by cross-group friendship and positive intergroup appraisals, mediated by intergroup emotions and out-group trust. In study 1, out-group friendship at time 1 predicted out-group trust at time 2 (one year later), controlling for prior out-group trust. In study 2, positive and negative intergroup emotions mediated the effects of friendship on positive and negative behavioral tendencies and attitudes. In study 3, a confirmatory factor analysis indicated that trust and emotions are distinct constructs with unique predictive contributions. We then tested a model in which cross-group friendship predicted intergroup emotions and trust through intimate self-disclosure in out-group friendships. Our findings support an integration of an intergroup emotions framework with research highlighting the importance of cross-group friendship in fostering positive intergroup outcomes.

**EXCLUDE (I reviewed this article to help develop the framework for this project. It has a few interesting points in the introduction, but overall, I don’t think it has a significant correlation to CCCs given the population studied and focus on political science - 2)** Nielsen, J.H.
Do Group Decision Rules Affect Trust? A Laboratory Experiment on Group Decision Rules and Trust
(2016) Scandinavian Political Studies, 39 (2), pp. 115-137.
https://www.scopus.com/inward/record.uri?eid=2-s2.0-84952683998&doi=10.1111%2f1467-9477.12058&partnerID=40&md5=c11226bbc676617ec14eb9e1065d841b
DOCUMENT TYPE: Article
SOURCE: Scopus

Enhanced participation has been prescribed as the way forward for improving democratic decision making while generating positive attributes like trust. Yet we do not know the extent to which rules affect the outcome of decision making. This article investigates how different group decision rules affect group trust by testing three ideal types of decision rules (i.e., a Unilateral rule, a Representative rule and a 'Non-rule') in a laboratory experiment. The article shows significant differences between the three decision rules on trust after deliberation. Interestingly, however, it finds that the Representative rule yields more trust than the Non-rule and also significantly more trust than the Unilateral rule, when analysing the results at group level. These findings challenge the theoretical understanding by, for example, deliberative normative theorists that more inclusive, consensual and non-hierarchical decision-making procedures enhance trust vis-à-vis other more hierarchical decision-making procedures.

**EXCLUDE (Virtual work teams in the setting of marketing and market research along with some of the leaders attributes studied in this paper are not really applicable to CCCs - 7)** Guinalíu, M., Jordán, P.
Building trust in the leader of virtual work teams [Generación de confianza en el líder de equipos de trabajos virtuales]
(2016) Spanish Journal of Marketing - ESIC, 20 (1), pp. 58-70.
https://www.scopus.com/inward/record.uri?eid=2-s2.0-85020294502&doi=10.1016%2fj.reimke.2016.01.003&partnerID=40&md5=0c432e5ea863bdbcc2cc22c6d604f390
DOCUMENT TYPE: Article
ACCESS TYPE: Open Access
SOURCE: Scopus

In recent years, due to the development of new technologies, virtual work teams have arisen as a new organizational form that offers businesses greater flexibility and adaptability in coping with new market challenges. The departments that manage high value-added projects are more susceptible to implementing virtual teams; the area of marketing and market research being one of them. However, the peculiarities of these teams present a real challenge for building trust within the team, with trust being one of the key factors for their success. Accordingly, this study considers various antecedent factors of trust toward leaders of virtual teams grouped in two blocks: the physical attributes (attractiveness) and the behavioral characteristics (justice and empathy) of the leader. Furthermore, the paper discusses how leadership style (transactional or transformational) can moderate the relationships between some of the previously mentioned variables. The results suggest a greater capacity for attractive, empathetic and just leaders to build trust. These results have interesting implications for management which are discussed along with the principle lines of future research.

**INCLUDE IN ARTICLE REVIEW (coded)** Mayfield, C.O., Tombaugh, J.R., Lee, M.
Psychological collectivism and team effectiveness: Moderating effects of trust and psychological safety
(2016) Journal of Organizational Culture, Communications and Conflict, 20 (1), pp. 78-94.
https://www.scopus.com/inward/record.uri?eid=2-s2.0-84974697586&partnerID=40&md5=bce56a8092fbc323c2c969a62856247a
DOCUMENT TYPE: Article
SOURCE: Scopus

The importance of emergent states and their influence on team functioning has become a focus for understanding various team outcomes. Using hierarchical linear, modeling we examine the moderating effects of two emergent states, team trust and psychological safety, on the relationship between psychological collectivism and team outcomes. Psychological collectivism is the internal orientation of an individual toward group goals, concern for group well-being, acceptance of group norms and a tendency toward cooperation in group contexts. Results from multilevel analysis of 58 teams of students (N=260) show that psychological collectivism is strongly related to team member evaluations of team satisfaction, team identity, and willingness to work with team members. Team trust and psychological safety moderated the relationship such that the effects of psychological collectivism were constrained in conditions of high trust and high safety. Implications for research and practice are discussed.

**EXCLUDE (This paper looks at various leadership styles in the setting of resource-maximization tasks, which doesn’t seem applicable to leadership or outcomes in a CCC setting - 4)** Boies, K., Fiset, J., Gill, H.
Communication and trust are key: Unlocking the relationship between leadership and team performance and creativity
(2015) Leadership Quarterly, 26 (6), pp. 1080-1094.
https://www.scopus.com/inward/record.uri?eid=2-s2.0-84939825129&doi=10.1016%2fj.leaqua.2015.07.007&partnerID=40&md5=006167639d1969c070a4301e7159d166
DOCUMENT TYPE: Article
SOURCE: Scopus

Considerable theoretical and empirical work has identified a relationship between transformational leadership and team performance and creativity. The mechanisms underlying this link, however, are not well understood. To identify the intervening processes inherent in this relationship, we experimentally manipulated the leadership style assigned to 44 teams taking part in a resource-maximization task. Teams were exposed either to a leader using inspirational motivation, intellectual stimulation, or a control condition. Our findings reveal important differences between leadership styles in communication and team outcomes (objective task performance and creativity). These results suggest that different dimensions of transformational leadership should be emphasized depending on the outcome sought. In addition, our results provide evidence for a sequential mediation model where leadership influences team outcomes through overall team communication and trust in teammates. This study suggests mechanisms by which transformational leaders may impact team outcomes, which has implications for team building and leadership training.

**INCLUDE IN ARTICLE REVIEW (This abstract was reviewed on the ERIC document)** Ennen, N.L., Stark, E., Lassiter, A.
The importance of trust for satisfaction, motivation, and academic performance in student learning groups
(2015) Social Psychology of Education, 18 (3), pp. 615-633.
https://www.scopus.com/inward/record.uri?eid=2-s2.0-84941421472&doi=10.1007%2fs11218-015-9306-x&partnerID=40&md5=bd27754ec0c44ee53b532a0b14a8a388
DOCUMENT TYPE: Article
SOURCE: Scopus

**EXCLUDE (This study focuses on distrust in the setting of newer virtual teams involved in short-term (vs longitudinal) decision-making. Although I feel there is value looking at virtual teams to some extent for this review, most CCCs are face to face, and this study seems to not apply to our research setting. - 7)** Lowry, P.B., Schuetzler, R.M., Giboney, J.S., Gregory, T.A.
Is Trust Always Better than Distrust? The Potential Value of Distrust in Newer Virtual Teams Engaged in Short-Term Decision-Making
(2015) Group Decision and Negotiation, 24 (4), pp. 723-752.
https://www.scopus.com/inward/record.uri?eid=2-s2.0-85027943459&doi=10.1007%2fs10726-014-9410-x&partnerID=40&md5=4df984b3a5c69b090bcd95c2b2d2e92b
DOCUMENT TYPE: Article
SOURCE: Scopus

The debate on the benefits of trust or distrust in groups has generated a substantial amount of research that points to the positive aspects of trust in groups, and generally characterizes distrust as a negative group phenomenon. Therefore, many researchers and practitioners assume that trust is inherently good and distrust is inherently bad. However, recent counterintuitive evidence obtained from face-to-face (FtF) groups indicates that the opposite might be true; trust can prove detrimental, and distrust instrumental, to decision-making in groups. By extending this argument to virtual teams (VTs), we examined the value of distrust for VTs completing routine and non-routine decision tasks, and showed that the benefits of distrust can extend to short-term VTs. Specifically, VTs seeded with distrust significantly outperformed all control groups in a non-routine decision-making task. In addition, we present quantitative evidence to show that the decision task itself can significantly affect the overall levels of trust/distrust within VTs. In addition to its practical and research implications, the theoretical contribution of our study is that it extends to a group level, and then to a VT setting, a theory of distrust previously tested in the psychology literature in the context of completing non-routine and routine decision tasks at an individual level.

**EXCLUDE (The population studied (undergraduate marketing/management students) using social cues for self-selected teams for a team assignment not really applicable to CCCs. We also excluded this based on title review on the ERIC sheet - 4)** Neu, W.A.
Social Cues of (Un)Trustworthy Team Members
(2015) Journal of Marketing Education, 37 (1), pp. 36-53.
https://www.scopus.com/inward/record.uri?eid=2-s2.0-84924911983&doi=10.1177%2f0273475314565509&partnerID=40&md5=4bc8a9afb6782c18463048cc06c60a66
DOCUMENT TYPE: Article
SOURCE: Scopus

This study investigates the way in which and the extent to which students engage in social categorization during the process of self-selecting team members for a team assignment. The discovery-oriented method of grounded theory was used. Data were gathered from a sample of 38 undergraduate marketing and management students using the Zaltman Metaphor Elicitation Technique. Results indicate that, when faced with having too little information about classmates, students use a variety of social cues to cognitively categorize classmates, and then make a series of inferences about their personality, values, and trustworthiness based on the category in which they are placed. In addition, students use the inferences to help make decisions about who to approach and who to avoid during the self-selection process. Making inferences about classmates based on the social category in which they are placed, rather than on individual merit, is stereotyping. Behaving differently toward classmates based on the social category in which they are placed is discrimination. All 38 students who participated in this study reported that they have used social cues to help decide who to approach and who to avoid during team formation, and 13 reported doing so every time they have self-selected team members.

**EXCLUDE (This article was reviewed on the PUBMED sheet)** Liu, G., Lin, C., Xin, Z.
The effects of within- and between-group competition on trust and trustworthiness among acquaintances
(2014) PLoS ONE, 9 (7), art. no. e103074, .
https://www.scopus.com/inward/record.uri?eid=2-s2.0-84904512211&doi=10.1371%2fjournal.pone.0103074&partnerID=40&md5=9845ab089de631adc3dd03ec7900a149
DOCUMENT TYPE: Article
ACCESS TYPE: Open Access
SOURCE: Scopus

**EXCLUDE (This article was reviewed on the PUBMED sheet)** Rolfe, A., Cash-Gibson, L., Car, J., Sheikh, A., Mckinstry, B.
Interventions for improving patients' trust in doctors and groups of doctors
(2014) Cochrane Database of Systematic Reviews, 2014 (3), art. no. CD004134, .
https://www.scopus.com/inward/record.uri?eid=2-s2.0-84907404462&doi=10.1002%2f14651858.CD004134.pub3&partnerID=40&md5=3a41b31dbc69288e53b23a4df61074ee
DOCUMENT TYPE: Review
SOURCE: Scopus

**INCLUDE IN ARTICLE REVIEW (coded)** Yang, I.
What makes an effective team? The role of trust (dis)confirmation in team development
(2014) European Management Journal, 32 (6), pp. 858-869.
https://www.scopus.com/inward/record.uri?eid=2-s2.0-84922428994&doi=10.1016%2fj.emj.2014.04.001&partnerID=40&md5=0bb88eb783f9ce36f948e2dc81d7f3cb
DOCUMENT TYPE: Article
SOURCE: Scopus

Most newly formed teams manage to function in spite of the fact that their members do not know each other. Over time, teams progress into successful units; however, sometimes, they regress into a situation where morale is worse than when the team was created. We explain how such opposing group outcomes can arise by examining team members' (dis)confirmation of expectations in line with the development of trust. We argue that the process of (dis)confirmation of expectations created based on early swift trust is crucial in defining the direction of team development (progression or regression) because it gives rise to emotions which further underpin (dis)trust. We present six sets of propositions which taken together construct a framework for understanding the role of (dis)confirmation and subsequent emotions during the process of trust updating and of team development. We provide a conceptual view of individuals' experiences within a team and their impact on team dynamics in a way which could form the basis of future empirical testing.

**EXCLUDE (I reviewed this article as one of the four to test out our coding document. I think it was originally written in Chinese and translated to English. It was hard to decifer parts, and Chinese self-management teams don’t seem directly applicable to CCCs - 2)** Wu, T., Wang, W., Bi, X., Liu, D.
Mediating effect of team trust between team conflict and team effectiveness in self-management teams
(2013) Journal of Applied Sciences, 13 (9), pp. 1504-1508.
https://www.scopus.com/inward/record.uri?eid=2-s2.0-84884497398&doi=10.3923%2fjas.2013.1504.1508&partnerID=40&md5=95ee794f0a2aec7c2d239f36a3c68e48
DOCUMENT TYPE: Article
SOURCE: Scopus

Team conflict is an amazing variable in team studies. But the effect of team conflict still remains debatable. Blessing or curse, it is not certain that team conflict is. It is reasonable that team type, team process and team context may influence the outcome of team conflict. In this study, 54 self-management teams were selected as participants, to explore the relationship between team conflict and team effectiveness. The result indicated that team conflict is medium negative correlated to team effectiveness and team trust is mediator between team conflict and team effectiveness. So, team conflict is harmful to team effectiveness in self-management team, but team trust may reduce the negative impact of team conflict.

**INCLUDE IN ARTICLE REVIEW (waiting to code - Virtual Teams - 7)** Jawadi, N.
E-leadership and trust management: Exploring the moderating effects of team virtuality
(2013) International Journal of Technology and Human Interaction, 9 (3), pp. 18-35.
https://www.scopus.com/inward/record.uri?eid=2-s2.0-84887461391&doi=10.4018%2fjthi.2013070102&partnerID=40&md5=a5d3090d1eb7fd8f92150fb7a9aacedc
DOCUMENT TYPE: Article
SOURCE: Scopus

Trust is considered a key factor in virtual team performance and outcomes. Recent studies suggest that eleaders significantly contribute to trust development in their teams and that their contributions depend on the team's level of virtuality. The purpose of this paper is to analyze the behaviors and practices that enable e-leaders to build trusty relationships in their teams. Using leadership behavioral complexity theory, we focus on the roles played by e-leaders in managing their teams. To this end, we conducted a large survey with virtual team members. The results highlight the importance of the roles of rational goals and human relations in trust management. With regard to the effects of virtuality, distance is found to have a significant negative moderator effect on the contribution of leadership to trust development, while the moderator effect of ICT use is positive.

**INCLUDE IN ARTICLE REVIEW (This article was reviewed on the ERIC sheet)** Selmer, J., Jonasson, C., Lauring, J.
Group conflict and faculty engagement: Is there a moderating effect of group trust?
(2013) Journal of Higher Education Policy and Management, 35 (1), pp. 95-109.
https://www.scopus.com/inward/record.uri?eid=2-s2.0-84873364015&doi=10.1080%2f1360080X.2013.748477&partnerID=40&md5=6181e31bda07550ffe9775a831f3d7b3
DOCUMENT TYPE: Article
SOURCE: Scopus

**EXCLUDE (This study focuses on a group setting in the setting of online learning. I feel this is a separate topic with limited applicability to CCCs - 7)** Tseng, H.W., Yeh, H.-T.
Team members' perceptions of online teamwork learning experiences and building teamwork trust: A qualitative study
(2013) Computers and Education, 63, pp. 1-9.
https://www.scopus.com/inward/record.uri?eid=2-s2.0-84871110834&doi=10.1016%2fj.compedu.2012.11.013&partnerID=40&md5=cc520ff955db2c1778cacf23d5928cdc
DOCUMENT TYPE: Article
SOURCE: Scopus

Teamwork factors can facilitate team members, committing themselves to the purposes of maximizing their own and others' contributions and successes. It is important for online instructors to comprehend students' expectations on learning collaboratively. The aims of this study were to investigate online collaborative learning experiences and to identify important factors that were crucial for building teamwork trust. A qualitative research method was utilized in the study. Data were collected from students' responses of three open-ended questions and interviews. The results indicated that students who enjoyed working in the group setting had a good relationship with their team members and they trusted their team members. In contrast, the questionable behaviors of members (lack of communication and low level of individual accountability) were negative factors of their teamwork experiences. In addition, students considered individual accountability, familiarity with team members, commitment toward quality work, and team cohesion were important factors for building trust with team members. Quantitative analyses confirmed that teamwork trust was correlated significantly with two of the important factors for building trust indicated by team members: familiarity with members (r = .74) and team cohesion (r = .79). Implications and recommendations for future research were also discussed.

**INCLUDE IN ARTICLE REVIEW (waiting to code - Virtual Teams - 7)** Brahm, T., Kunze, F.
The role of trust climate in virtual teams
(2012) Journal of Managerial Psychology, 27 (6), pp. 595-614.
https://www.scopus.com/inward/record.uri?eid=2-s2.0-84865129833&doi=10.1108%2f02683941211252446&partnerID=40&md5=5bc5bb9a4af3bda840cfd4bea0a17b90
DOCUMENT TYPE: Article
SOURCE: Scopus

Purpose: Research testing a complex process model, incorporating moderating and mediating mechanisms associated with virtual team (VT) performance, remains rare. This paper aims to introduce trust climate as a crucial boundary condition for high performance in VTs. It also aims to propose a moderated-indirect model such that the relationship between team goals and task performance is mediated by task cohesion and the relationship between team goals and task cohesion is moderated by trust. Design/methodology/approach: Hypotheses are tested using a longitudinal design with a sample of 50 teams. Findings: The proposed moderated-indirect model is confirmed. The model explains the indirect relationship between team goal setting and performance transmitted through task cohesion, which is dependent on the level of trust climate. Research limitations/implications: Although hypotheses were tested in a longitudinal setting, common source bias might be a potential problem for some of the observed relationships. Future research could build on this model for further investigations on more complex theoretical models for VT performance. Practical implications: This research suggests that managers should emphasize the development of team trust at early stages of collaboration in a VT to reach high performance outcomes. Societal implications: For VTs, trustful working environments should become even more important in the future, supporting team members' satisfaction in working in VTs. Originality/value: Through this study, a complex process model for VTs was developed and trust climate established as a prominent context factor for VT success.

**EXCLUDE (This paper looks looks at dyads of college students working on a computer-based truck dispatching task, deciding as a team which task activities to perform and in what order. It doesn’t really seem applicable to CCCs - 2)** Narayan, A., Steele-Johnson, D.
Individual and relational self-concepts in a team context: Effects on task perceptions, trust, intrinsic motivation, and satisfaction
(2012) Team Performance Management: An International Journal, 18 (5-6), pp. 236-255.
https://www.scopus.com/inward/record.uri?eid=2-s2.0-84865117505&doi=10.1108%2f13527591211251122&partnerID=40&md5=2931e13cb31155db72ee3ec9f3fac66e
DOCUMENT TYPE: Article
SOURCE: Scopus

The purpose of this article is to understand the role of individual and relational self-concepts on various team processes and outcomes in a team context. Participants (n=470) worked in dyads on a computer-based truck dispatching task, deciding as a team which task activities to perform and in what order. The authors assessed differential relationships between individual and relational self-concepts and various team processes (e.g. trust) and outcomes (satisfaction). Subjective task complexity was influenced primarily by individual self-concept, specifically their core self-evaluations. Trust in others was influenced primarily by individuals' relational self-concepts, specifically their teamwork predisposition. Intrinsic motivation and satisfaction were influenced by both individual and relational self-concepts. Future research should examine these effects in teams larger than dyads, with other types of tasks, over longer time periods, and with non-college student samples. Depending on the task type, a practitioner might cue different self-concepts to increase individuals' focus on team performance, individual performance, or both. For example, if the team task is highly interdependent and reciprocal in nature, then the team can be trained together or provided information to cue relational self-concept. This paper focuses on the construct of individual and relational self-concepts and their effects on individual functioning in a team context. The results support and extend prior research by demonstrating that outcomes in a team context can be identified and examined in relation to individual conceptions of the self, relational conceptions of the self, or by both.

**INCLUDE IN ARTICLE REVIEW (coded)** Seppälä, T., Lipponen, J., Pirttilä-Backman, A.-M.
Leader fairness and employees' trust in coworkers: The moderating role of leader group prototypicality
(2012) Group Dynamics, 16 (1), pp. 35-49.
https://www.scopus.com/inward/record.uri?eid=2-s2.0-84880759747&doi=10.1037%2fa0026970&partnerID=40&md5=7f8d86c70f4f4b305072a6d6c11608e0
DOCUMENT TYPE: Article
SOURCE: Scopus

In this article, the association between perceived supervisor fairness and trust in coworkers as a collective entity is studied. Based on identity-related theories on fairness, trust, and leader effectiveness it was hypothesized that perceived supervisor distributive, procedural, and interactional fairness are positively and more strongly related to employee trust in their coworkers if the supervisor is highly group prototypical rather than less group prototypical. An empirical study, conducted with 176 employees within 30 work groups, supported this hypothesis. Fairness of a less group prototypical supervisor was not associated with trust in coworkers, whereas especially unfairness of the group prototypical supervisor was detrimental for trust in coworkers. The study concludes that leader's prototypicality might not work as a substitute for fairness, as some recent studies have suggested, when the outcome is not directly related to the assessment of the leader. Thus, leaders should not count on the trust they earn by being group prototypical but they should also aim at fairness. Implications for collective distrust theory (Kramer, 1994, 1998) are also discussed.

**INCLUDE IN ARTICLE REVIEW (coded)** Carmeli, A., Tishler, A., Edmondson, A.C.
CEO relational leadership and strategic decision quality in top management teams: The role of team trust and learning from failure
(2012) Strategic Organization, 10 (1), pp. 31-54.
https://www.scopus.com/inward/record.uri?eid=2-s2.0-84856615882&doi=10.1177%2f1476127011434797&partnerID=40&md5=3277104fd9e6673d7839d4829c104701
DOCUMENT TYPE: Article
SOURCE: Scopus

In this study, we examine a complex pathway through which CEOs, who exhibit relational leadership, may improve the quality of strategic decisions of their top management teams (TMTs) by creating psychological conditions of trust and facilitating learning from failures in their teams. Structural equation modeling (SEM) analyses of survey data collected from 77 TMTs indicate that (1) the relationship between CEO relational leadership and team learning from failures was mediated by trust between TMT members; (2) team learning from failures mediated the relationship between team trust and strategic decision quality. Supplemented by qualitative data from two TMTs, these findings suggest that CEOs can improve the quality of strategic decisions their TMTs make by shaping a relational context of trust and facilitating learning from failures.

**EXCLUDE (This study looks a moderators that might influence trust of in-group members versus out-group members, which doesn’t really seem applicable to CCCs - 4)** Platow, M.J., Foddy, M., Yamagishi, T., Lim, L., Chow, A.
Two experimental tests of trust in in-group strangers: The moderating role of common knowledge of group membership
(2012) European Journal of Social Psychology, 42 (1), pp. 30-35.
https://www.scopus.com/inward/record.uri?eid=2-s2.0-84856219249&doi=10.1002%2fejsp.852&partnerID=40&md5=66f0e058c00a74a0a602acf8c45c819b
DOCUMENT TYPE: Article
SOURCE: Scopus

The role that shared group membership plays in decisions to trust others is now well established within social psychology. A close reading of this literature, however, shows that this process is often moderated by other variables. Currently, we examined one potential moderator of this process. In particular, we evaluated the role that common knowledge of a shared social group membership between self and a to-be-trusted stranger provides as a basis for trusting this stranger. This common knowledge emerges when the truster knows the group membership of the to-be-trusted other, and believes that this other also knows the group membership of the truster. In two experiments, using pre-existing and minimal groups, we show that people are more likely to trust an in-group member over an out-group member under conditions of common group-membership knowledge rather than private group-membership knowledge (i.e. other does not know truster's group), even when they could choose not to trust anyone. The manner in which these data add to current understandings of group-based trust in strangers is discussed.

**EXCLUDE (This article looks at computer modeling to assess decision-making processes of virtual teams, not really applicable to CCCs - 7)** Martínez-Miranda, J., Pavón, J.
Modeling the influence of trust on work team performance
(2012) SIMULATION, 88 (4), pp. 408-436.
https://www.scopus.com/inward/record.uri?eid=2-s2.0-84860433278&doi=10.1177%2f0037549711404714&partnerID=40&md5=a7ff1a618dae68dfab90557f7ddd64e9
DOCUMENT TYPE: Article
SOURCE: Scopus

The selection of suitable people to make up a successful work team is not a trivial decision-making process, because of the diversity and complexity of the factors that influence individual and team performance. Teams are assembled by project managers using their experience and the information they have available (although this is frequently scarce, uncertain, and dynamic) about the personal and professional characteristics of potential team members. In this paper, we introduce an agent-based model developed to support this decision-making process where a virtual team can be configured using some selected characteristics of potential team members. Fuzzy sets and fuzzy rules are used to model the interaction between team members and a given set of tasks, generating statistical information that represents possible performance by team members. More specifically, the paper focuses on the concept of trust, an important social skill that influences performance at individual (team member) and global (work team) levels. We describe the implementation of an agent-based simulation system where the user can test different team configurations to compare performance and select the best possible work team for a given project. The evaluation and validation of the model was performed through face validity and historical data validation techniques, which are based on information collected from real work teams. The results show the suitability of the model as a helpful tool in the formation and configuration of work teams for specific scenarios.

**INCLUDE IN ARTICLE REVIEW (This article was reviewed on the ERIC sheet)** Berry, G.R.
A cross-disciplinary literature review: Examining trust on virtual teams
(2011) Performance Improvement Quarterly, 24 (3), pp. 9-28.
https://www.scopus.com/inward/record.uri?eid=2-s2.0-80155203013&doi=10.1002%2fpiq.20116&partnerID=40&md5=dff24f311e0669be98734caabf3fd19d
DOCUMENT TYPE: Article
SOURCE: Scopus

**EXCLUDE (Computer science focused article - 7)** Fan, Z.-P., Suo, W.-L., Feng, B., Liu, Y.
Trust estimation in a virtual team: A decision support method
(2011) Expert Systems with Applications, 38 (8), pp. 10240-10251.
https://www.scopus.com/inward/record.uri?eid=2-s2.0-79953684545&doi=10.1016%2fj.eswa.2011.02.060&partnerID=40&md5=e65ce6a872b8b5fe6726ca66f666e4ef
DOCUMENT TYPE: Article
SOURCE: Scopus

Trust plays an important role in the operation and management of virtual teams. It is also a foundation of the cooperation between members. In the operation of a virtual team, trust estimation is a very significant issue to know about the trustworthiness of each member and cooperation situations between members. The purpose of this paper is to propose a decision support method for estimating the trust level. A trust estimation framework with two dimensions of reputation and collaboration is developed. From this, a fuzzy multiattribute decision analysis (MADA) approach based on the 2-tuple fuzzy linguistic representation model is proposed to measure the performances on reputation and collaboration. By the measurements of the performances, the trust level of each member and the virtual team can be identified so as to improve the management of the virtual team. Furthermore, a Web-based trust estimation system (WTES) is designed and developed to support the activities of trust estimation in a virtual team. Finally, an application is provided to illustrate the applicability of the proposed decision support method.

**INCLUDE IN ARTICLE REVIEW (coded)** Walumbwa, F.O., Luthans, F., Avey, J.B., Oke, A.
Authentically leading groups: The mediating role of collective psychological capital and trust
(2011) Journal of Organizational Behavior, 32 (1), pp. 4-24.
https://www.scopus.com/inward/record.uri?eid=2-s2.0-78650426146&doi=10.1002%2fjob.653&partnerID=40&md5=c5f0e7003b688616c39b1778ec116501
DOCUMENT TYPE: Article
SOURCE: Scopus

Although there have been recent theoretical advances in what is increasingly being recognized as authentic leadership, research testing possible mediating processes and the impact on group-level outcomes has not received attention. To help address this need, this study examined at the group level of analysis the role that collective psychological capital and trust may play in the relationship between authentic leadership and work groups' desired outcomes. Utilizing 146 intact groups from a large financial institution, the results indicated a significant relationship between both their collective psychological capital and trust with their group-level performance and citizenship behavior. These two variables were also found to mediate the relationship between authentic leadership and the desired group outcomes, even when controlling for transformational leadership. Implications for future research and practice conclude the paper.

**EXCLUDE (This article is focused on virtual teams, specifically distributed softward development teams, with minimal applicability to CCCs - 7)** Miner-Rubino, K., Reed, W.D.
Testing a Moderated Mediational Model of Workgroup Incivility: The Roles of Organizational Trust and Group Regard
(2010) Journal of Applied Social Psychology, 40 (12), pp. 3148-3168.
https://www.scopus.com/inward/record.uri?eid=2-s2.0-78650217337&doi=10.1111%2fj.1559-1816.2010.00695.x&partnerID=40&md5=9c4374df654d4bfda2754e0d9be775d4
DOCUMENT TYPE: Article
SOURCE: Scopus

Today globally distributed software development has become the norm for many organizations and the popularity of implementing such an approach continues to increase. In these circumstances a strategy often employed is the use of virtual software development teams. Due to the collaborative nature of software development this has proved a difficult and complex endeavor. Research has identified distance in its various forms as an important factor which negatively impacts on global software development and on virtual software team operation in particular. In this context, the aspects of distance have been defined as temporal, geographical, cultural and linguistic. A key element for the success of any team based project is the development of trust and cooperation. Each aspect of distance can negatively impact on the development of trust and hamper cooperation particularly in the virtual team environment. An additional factor which this research identified is the importance and negative impact fear plays. The serious implications of these factors are due to the need for dependence on asynchronous and online communication which is inherent to global software development and the operation of virtual software teams in particular. The findings presented here are the results from four independent studies undertaken over a twelve year period which consider each of these issues. Having identified the problems associated with trust and communication, how these issues were successfully addressed and managed on a multimillion dollar project which was heading for failure is outlined.

**EXCLUDE (This study focuses on ad hoc, virtual teams, which is not similar to the longitudinal structure of CCCs - 7)** Altschuller, S., Benbunan-Fich, R.
Trust, Performance, and the Communication Process in Ad Hoc Decision-Making Virtual Teams
(2010) Journal of Computer-Mediated Communication, 16 (1), pp. 27-47.
https://www.scopus.com/inward/record.uri?eid=2-s2.0-79951820476&doi=10.1111%2fj.1083-6101.2010.01529.x&partnerID=40&md5=9b0f7ef4027f2cc13ad05705c493381a
DOCUMENT TYPE: Article
SOURCE: Scopus

This research explores the communication process variables that potentially determine trust and performance quality in ad hoc virtual teams to better inform the choice of communication media for virtual groups engaged in decision-making tasks. Results of a survey indicate that virtual copresence is significantly correlated with intrateam trust and the performance quality that teams achieve. Results also accentuate the prominent role of trust in the virtual collaborative decision-making process; they indicate that trust mediates the relationship between virtual copresence and performance. It is therefore recommended that the efforts related to the design and adoption of communication systems for decision making teams strongly consider that virtual copresence potentially promotes both high levels of trust and high quality performance in virtual teams.

**EXCLUDE (This study focuses on financial incentives and audit committee members, neither of which are applicable to CCCs - 4)** Rose, A.M., Rose, J.M., Dibben, M.
The effects of trust and management incentives on audit committee judgments
(2010) Behavioral Research in Accounting, 22 (2), pp. 87-103.
https://www.scopus.com/inward/record.uri?eid=2-s2.0-80052774176&doi=10.2308%2fbria.2010.22.2.87&partnerID=40&md5=72255b7fa8f0a866d2038c3478f6e680
DOCUMENT TYPE: Article
SOURCE: Scopus
We investigate the effects of audit committee members' dispositional trust and management incentives on audit committee judgments. Results of an experiment indicate that: (1) When management has incentives to manage earnings, less trusting audit committee members are more likely to support the external auditor than are more trusting audit committee members; (2) when management has incentives to manage earnings, less trusting audit committee members are more likely to perceive that management is not credible, and more likely to perceive that management is being deceptive than are more trusting audit committee members; and (3) the interactive effects of trust and incentives on decisions to support the auditor are mediated by perceptions of management's intent to deceive. That is, less trusting audit committee members perceive that management's incentives to manage earnings result in potential deception by management, and less trusting audit committee members increase their support for the auditor because of concerns about management deception.

**INCLUDE IN ARTICLE REVIEW (This article was reviewed on the PUBMED document)** Terwel, B.W., Harinck, F., Ellemers, N., Daamen, D.D.L.
Voice in Political Decision-Making: The Effect of Group Voice on Perceived Trustworthiness of Decision Makers and Subsequent Acceptance of Decisions
(2010) Journal of Experimental Psychology: Applied, 16 (2), pp. 173-186.
https://www.scopus.com/inward/record.uri?eid=2-s2.0-77953899827&doi=10.1037%2fa0019977&partnerID=40&md5=afce3020d7f94a80a20f0edc67602c37
DOCUMENT TYPE: Article
SOURCE: Scopus

**INCLUDE IN ARTICLE REVIEW (coded)** Dayan, M., Di Benedetto, C.A.
The impact of structural and contextual factors on trust formation in product development teams
(2010) Industrial Marketing Management, 39 (4), pp. 691-703.
https://www.scopus.com/inward/record.uri?eid=2-s2.0-77952886151&doi=10.1016%2fj.indmarman.2010.01.001&partnerID=40&md5=9fd4ecbee73bee7d2413acc4c6e42617
DOCUMENT TYPE: Article
SOURCE: Scopus

This study examines antecedents of trust formation in new product development (NPD) teams and the effects of trust on NPD team performance. A theoretical framework relating structural and contextual factors to interpersonal trust and project outcomes was built, including task complexity as a moderating variable. Hypotheses from this model were tested with data on 93 product development projects carried out in Turkey. The findings showed that structural factors such as moderate level of demographic diversity, proximity of team members, team longevity, and contextual factors (procedural and interactional justices) were positively related to the development of interpersonal trust in NPD teams. The findings also revealed that interpersonal trust had an impact on team learning and new product success, but not on speed-to-market. Further, the findings showed that the impact of interpersonal trust on team learning and new product success was higher when there was higher task complexity. Theoretical and managerial implications of the study findings are discussed.

**INCLUDE IN ARTICLE REVIEW (coded)** Lowry, P.B., Zhang, D., Zhou, L., Fu, X.
Effects of culture, social presence, and group composition on trust in technology-supported decision-making groups
(2010) Information Systems Journal, 20 (3), pp. 297-315.
https://www.scopus.com/inward/record.uri?eid=2-s2.0-84863337613&doi=10.1111%2fj.1365-2575.2009.00334.x&partnerID=40&md5=a43a947d3c263b71dd87ad32898839ff
DOCUMENT TYPE: Article
SOURCE: Scopus

This study examines trust in technology-supported groups from the perspectives of culture, social presence and group composition. Our results demonstrate that, in culturally homogeneous groups, individualism has a negative impact on the level of interpersonal trust; however, in culturally heterogeneous groups consisting of Chinese and U.S. participants, individualism has a positive impact on interpersonal trust among members. There were also significant differences in the level of trust between homogeneous Chinese groups and heterogeneous groups consisting of Chinese and U.S. participants. In addition, the mediating role of communication quality was identified to explain the effect for trust-of social presence on trust. These findings have important implications on building and communication in global technology-supported decision-making groups.

**EXCLUDE (Although I opted to include the other Dayan article above, this one looking a team commitment and longevity in the setting of product development teams does not seem to apply very well to CCCs - 2)** Dayan, M.
Managerial trust and NPD team performance: Team commitment and longevity as mediators
(2010) Journal of Business and Industrial Marketing, 25 (2), pp. 94-105.
https://www.scopus.com/inward/record.uri?eid=2-s2.0-75649114561&doi=10.1108%2f08858621011017723&partnerID=40&md5=daa1a5605ef7a53f6369b326177415c8
DOCUMENT TYPE: Article
SOURCE: Scopus

Purpose: The purpose of this paper is to examine the mediating effects of team commitment and longevity between managerial trust and team performance (e.g. team learning and product success), using environmental turbulence as a moderator. Design/methodology/approach: To test the proposed model, data were collected from 335 team members and team leaders of 107 Turkish new product/project development teams. Findings: The results of the structural equation model showed that managerial trust (as rated by team members) was significantly associated with team commitment and longevity; and team commitment and longevity significantly mediated the relationships between managerial trust and team learning, and managerial trust and product success. Moreover, the findings showed that the impact of managerial trust on team commitment and longevity was higher when environmental turbulence was high. However, the mediating impact of team commitment was significant regardless of market or technical turbulence; and team longevity had a significant mediating impact if environment was turbulent. Theoretical and managerial implications of the study findings were discussed at the end. Originality/value: The novelty of the research lies in the empirical test of managerial trust in the context of teams with innovation projects (e.g. product development teams). Furthermore, the inclusion of team commitment and longevity as moderators represents an added value of the study.

**EXCLUDE (This study looks at virtual project teams and the way they analyze the idea of trust (along with the title of the journal) makes it sound like this is more of a computer science-based field, which is not really applicable to CCCs - 7)** Chen, T.-Y., Chen, Y.-M.
Advanced multi-phase trust evaluation model for collaboration between coworkers in dynamic virtual project teams
(2009) Expert Systems with Applications, 36 (8), pp. 11172-11185.
https://www.scopus.com/inward/record.uri?eid=2-s2.0-67349252995&doi=10.1016%2fj.eswa.2009.02.090&partnerID=40&md5=bc1ce97e231a61abff7db32783b71cd8
DOCUMENT TYPE: Article
SOURCE: Scopus

With the globalization of commercial practices and advances in information and communication technologies, increasing numbers of enterprises and workers are establishing cross-functional, geographically distributed virtual project teams (VPTs) to maximize competitive advantage from limited labor and resources. One of the major challenges for workers in VPT is developing trust. Trust is a particularly crucial issue for VPT success because numerous project tasks are interdependent, making team members reliant on the functional expertise of their partners. The challenge to develop trust is particularly complex because trust should consider various factors into different assessments made during different phases of a VPT. This study first introduces a previously proposed trust evaluation model for evaluating trust over the lifecycle of VPTs. The original method, which comprises a method for evaluating trust between two VPT workers and a method for evaluating worker reliability, in which direct, indirect and negative trust relationships are considered in calculating trust between workers. This study aims to improve this method and then design an advanced multi-phase trust evaluation model, which comprises calculus-based, contribution-based and affective-based trust evaluation sub-models for evaluating trust during three different VPT phases (early, middle and late project phases). The advanced multi-phase trust evaluation model is a more appropriate means of assessing degree of trust among coworkers than the original model. Therefore, using the three sub-models during different project phases can assist VPT workers in making more accurate decisions regarding secure resource sharing with other coworkers

**INCLUDE IN ARTICLE REVIEW (waiting to code - Virtual Teams - 7)** Bierly III, P.E., Stark, E.M., Kessler, E.H.
The moderating effects of virtuality on the antecedents and outcome of NPD team trust
(2009) Journal of Product Innovation Management, 26 (5), pp. 551-565.
https://www.scopus.com/inward/record.uri?eid=2-s2.0-67650745193&doi=10.1111%2fj.1540-5885.2009.00680.x&partnerID=40&md5=8d4f131424e921c96f5684a38fa0c5d3
DOCUMENT TYPE: Article
SOURCE: Scopus

The fundamental dynamics of virtual and traditional face-to-face teams may be very different. The purpose of this study is to empirically examine and assess the moderating effects of virtuality on the antecedents and outcome of trust, where virtuality is measured along a continuum from face to face (no virtuality) to fully virtual rather than the more common approach of dichotomizing teams into two groups (i.e., face to face and virtual). The sample includes 116 different new product development teams from a variety of industries. The antecedents of trust that are studied are familiarity, goal clarity, training, relationship conflict, and process conflict. The outcome of trust is analyzed by determining how the impact of trust on cooperation changes as the level of virtuality changes. Primary findings are as follows: (1) Relationship conflict can be more detrimental to virtual teams than face-to-face teams because it is very difficult for team members of virtual teams to resolve their interpersonal disputes; (2) goal clarity is more important for face-to-face teams and less important for virtual teams in creating trust among team members; and (3) the impact of trust on cooperation is less for virtual teams than face-to-face teams. The primary implication for researchers and practice of these findings is that the role and importance of trust in virtual teams needs to be reevaluated. Managers using virtual teams need to realize that interpersonal relationships in virtual teams do not evolve in the same manner as face-to-face teams and may require different management techniques to be successful.

**INCLUDE IN ARTICLE REVIEW (waiting to code - Virtual Teams - 7)** Mitchell, A., Zigurs, I.
Trust in Virtual Teams: Solved or Still a Mystery?
(2009) Data Base for Advances in Information Systems, 40 (3), pp. 61-83.
https://www.scopus.com/inward/record.uri?eid=2-s2.0-79952965247&doi=10.1145%2f1592401.1592407&partnerID=40&md5=6d0d7944ec699a712c8e4c207e3c8d48
DOCUMENT TYPE: Article
SOURCE: Scopus

Trust was one of the most prominent topics of early research in virtual teams, highlighted by the emergence of the theory of swift trust. As researchers continue to study this topic, it is important to understand what problems have been solved as well as what aspects of trust in virtual teams remain a mystery. We address this goal by presenting an in-depth analysis of existing research on trust in virtual teams. We review theories, methods, tasks, technologies, and major outcome themes from 42 empirical studies over an eleven- year period from 1997 through 2007. The analysis reveals gaps and areas for future research, including the need for additional theorizing, broader use of methods, deliberate interventions that enhance trust, and continuing evolution of the trust concept.

**EXCLUDE (This article focuses on temporary, work-oriented virtual teams and is not really applicable to CCCs - 7)** Kuo, F.-Y., Yu, C.-P.
An exploratory study of trust dynamics in work-oriented virtual teams
(2009) Journal of Computer-Mediated Communication, 14 (4), pp. 823-854.
https://www.scopus.com/inward/record.uri?eid=2-s2.0-68849090229&doi=10.1111%2fj.1083-6101.2009.01472.x&partnerID=40&md5=c2e6c98dbc79e67e34d611180a4293a4
DOCUMENT TYPE: Article
SOURCE: Scopus

This study explores trust development and maintenance in temporary, work-oriented virtual teams, and examines the effect of trust on communication and cohesiveness. Results suggest that for work-oriented virtual teams formed on a temporary basis, members swiftly develop calculus-based trust in order to assess the outcomes and costs of maintaining team relationships. Members also rely on prior knowledge to determine other members' competence so that they can make predictions about one another's behaviors. Thus, both calculus-based and knowledge-based trust play accentuating roles in the initial development of work-oriented virtual teams. Identification-based trust also develops swiftly initially, but is relatively insignificant compared to the other two types of trust. Finally, initial trust may correlate to both later communication and later cohesiveness.

**EXCLUDE (This article looks at virtual teams, trust between team members, and identification with the team. Although these are potentially important concepts, we have newer papers identified to look at virtual teams, and I think this one is low yield. - 7)** Hakonen, M., Lipponen, J.
It takes two to tango: The close interplay between trust and identification in predicting virtual team effectiveness
(2009) Journal of E-working, 3 (1), .
https://www.scopus.com/inward/record.uri?eid=2-s2.0-66549083591&partnerID=40&md5=d7de17d1681cbc88461f184bc6a52456
DOCUMENT TYPE: Article
SOURCE: Scopus

The purpose of this study was to examine how trust between the team-members and identification with the team are related to the effectiveness of virtual teams. The literature suggests that both trust and identification are crucial for success of virtual teams but there is a lack of empirical studies to substantiate this assumption. We hypothesized that the identification-effectiveness link should be stronger under high-trust than under low-trust conditions, and that the relationship between trust and effectiveness should be stronger when team members identify strongly with the team. In our study based on a cross-sectional survey methodology and data aggregated to team level (N = 31), we found clear support for our hypotheses.

**INCLUDE IN ARTICLE REVIEW (coded)** van der Zee, K., Vos, M., Luijters, K.
Social identity patterns and trust in demographically diverse work teams
(2009) Social Science Information, 48 (2), pp. 175-198.
https://www.scopus.com/inward/record.uri?eid=2-s2.0-67949124873&doi=10.1177%2f0539018409102406&partnerID=40&md5=04aa2f0926278a916d0bd5d00a033b38
DOCUMENT TYPE: Article
SOURCE: Scopus

The article presents a model that links trust in a demographically diverse work context to three different social-identity patterns. Trust is considered to be beneficial for interpersonal relationships and work outcomes in diverse teams as well as for a healthy work relationship between minority members and their company. First, imposing a common ingroup identity based on similarities has been put forward as a useful method of creating depersonalized forms of trust among members of different demographic subgroups. However, its usefulness seems to be limited to situations of low identity threat. Alternatively, recent findings support the usefulness of creating a relational identity orientation or a common ingroup identity that explicitly embraces the value of diversity. The latter methods seem to enforce more personalized and more robust forms of identity-based trust in teams. They may also be useful in promoting trust of minority members in the organizational setting as a whole and in its authorities, probably because these identity patterns contribute to feelings of respect among minority members. Practical implications of these findings are discussed.

**EXCLUDE (The concepts of depersonalized ingroup trust, group importance, and anxiety, don’t seem to really fit well with CCCs - 2)** Kenworthy, J.B., Jones, J.
The roles of group importance and anxiety in predicting depersonalized ingroup trust
(2009) Group Processes and Intergroup Relations, 12 (2), pp. 227-239.
https://www.scopus.com/inward/record.uri?eid=2-s2.0-61749083824&doi=10.1177%2f1368430208101058&partnerID=40&md5=27533bebcb030112c3e9d54b35ae24de
DOCUMENT TYPE: Article
SOURCE: Scopus

In this experiment, we examine depersonalized ingroup trust as a function of group importance and anxiety. We expected that depersonalized ingroup trust would be stronger in more important social groups. We further predicted that anxiety would moderate the relationship between group importance and depersonalized ingroup trust, so that in high-importance social groups, but not within low-importance social groups, anxiety would increase depersonalized ingroup trust. By contrast, for low importance groups, anxiety should not affect depersonalized trust, but should increase interpersonal trust instead. These predictions were supported, even after controlling for baseline levels of general trust in others as well as for the presence of non-anxiety emotion states. The article discusses the theoretical and empirical links between anxiety, uncertainty, and decisions to trust in collective contexts.

**EXCLUDE (This study looks at senior business students operating in computer-mediated dyads while performing a strategic decision-making simulation, all of which really don’t apply to CCCs - 7)** Hill, N.S., Bartol, K.M., Tesluk, P.E., Langa, G.A.
Organizational context and face-to-face interaction: Influences on the development of trust and collaborative behaviors in computer-mediated groups
(2009) Organizational Behavior and Human Decision Processes, 108 (2), pp. 187-201.
https://www.scopus.com/inward/record.uri?eid=2-s2.0-60649098951&doi=10.1016%2fj.obhdp.2008.10.002&partnerID=40&md5=9ead5913f268a175eaadba1013013879
DOCUMENT TYPE: Article
SOURCE: Scopus

Using adaptive structuration theory as a framework [DeSanctis, G., & Poole, M. S. (1994). Capturing the complexity in advanced technology use: Adaptive structuration theory. Organization Science, 5(2), 121-147], we examined the influence of organizational context (competitive versus cooperative) and introductory meeting communication medium (face-to-face versus electronic) on the development of trust and collaborative behaviors of dyads communicating electronically. Based on a sample of 208 senior business students operating in computer-mediated (CM) dyads while performing a strategic decision-making simulation, we found that context, introductory communication medium, and time interacted to influence trust and collaborative behaviors. The pattern of development of trust and collaborative behaviors differed for the same introductory communication medium in different context conditions. Further, the level of trust and collaboration for dyad members in the competitive/electronic introductory meeting condition lagged significantly behind those in the other conditions. The findings suggest that introductory face-to-face interaction plays a more important role in facilitating the development of trust and collaboration in a CM environment when the context is competitive.

**EXCLUDE (I reviewed this study to help build the framework. The trust game and impact of the research results really don’t apply very well to a CCC setting - 4).** Song, F.
Intergroup trust and reciprocity in strategic interactions: Effects of group decision-making mechanisms
(2009) Organizational Behavior and Human Decision Processes, 108 (1), pp. 164-173.
https://www.scopus.com/inward/record.uri?eid=2-s2.0-57249103575&doi=10.1016%2fj.obhdp.2008.06.005&partnerID=40&md5=073e68ede4d8e5e6b8a8bc67a6d672da
DOCUMENT TYPE: Article
SOURCE: Scopus

I examined the impact of the consensus-making mechanism, where members reach a common decision via an intra-group discussion, on intergroup trust and reciprocity in a strategic setting. Data from a trust game generated the following results. First, compared to individual decision-makers, consensus groups exhibited (a) lower psychological trust, (b) higher behavioral trust, after controlling for psychological trust, and (c) lower reciprocity. Second, compared to decisions made by group-representatives, who are responsible for unilateral decisions on behalf of their groups, group consensus decisions were more trusting but less reciprocating. Thus, the specific decision-making mechanism adopted by groups in a strategic interaction may profoundly change the nature and the interplay of the interaction. Lastly, results show that the level of behavioral trust is driven by reciprocity expectations, while the level of reciprocity behavior, measured as a proportion of the trust received, does not change systematically with the level of trust experienced.

**INCLUDE IN ARTICLE REVIEW (waiting to code - Virtual Teams - 7)** Staples, D.S., Webster, J.
Exploring the effects of trust, task interdependence and virtualness on knowledge sharing in teams
(2008) Information Systems Journal, 18 (6), pp. 617-640.
https://www.scopus.com/inward/record.uri?eid=2-s2.0-39049139749&doi=10.1111%2fj.1365-2575.2007.00244.x&partnerID=40&md5=084442d3e5749311e3b4124beeb5866a
DOCUMENT TYPE: Article
SOURCE: Scopus

The sharing of knowledge within teams is critical to team functioning. However, working with team members who are in different locations (i.e. in virtual teams) may introduce communication challenges and reduce opportunities for rich interactions, potentially affecting knowledge sharing and its outcomes. Therefore, using questionnaire-based data, this study examined the potential effects of different aspects of virtuality on a knowledge-sharing model. Social exchange theory was used to develop a model relating trust to knowledge sharing and knowledge sharing to team effectiveness. The moderating effects of virtuality and task interdependence on these relationships were examined. A strong positive relationship was found between trust and knowledge sharing for all types of teams (local, hybrid and distributed), but the relationship was stronger when task interdependence was low, supporting the position that trust is more critical in weak structural situations. Knowledge sharing was positively associated with team effectiveness outcomes; however, this relationship was moderated by team imba lance and hybrid structures, such that the relationship between sharing and effectiveness was weaker. Organizations should therefore avoid creating unbalanced or hybrid virtual teams.

**EXCLUDE (This study focuses on virtual project teams, is close to 10 years old, and doesn’t seem as applicable for CCCs as we have included above. - 7)** Chen, T.-Y., Chen, Y.-M., Chu, H.-C.
Developing a trust evaluation method between co-workers in virtual project team for enabling resource sharing and collaboration
(2008) Computers in Industry, 59 (6), pp. 565-579.
https://www.scopus.com/inward/record.uri?eid=2-s2.0-43949093061&doi=10.1016%2fj.compind.2008.01.001&partnerID=40&md5=71af6c163fd62e574dad35791a03c1ec
DOCUMENT TYPE: Article
SOURCE: Scopus

An increasing number of companies, especially those with knowledge-intensive R & D programs, have turned to virtual project teams (VPTs) in recent years to generate the greatest competitive advantage from limited labor and resources. Members of a VPT are not limited to an enterprise and usually have various core capabilities. Successful implementation of a VPT depends on collaboration among team members during a project; such collaboration relies on efficient management of distributed resources, safe and timely resource sharing, and reliable trust evaluation mechanisms that facilitate the greatest degree of information sharing and minimize information transfer delays. This study first identifies VPT characteristics and the particular requirements of effective resource management and sharing. A VPT model is then presented. By applying this model, this study presents a trust evaluation method that assists VPT members in determining whether resource holders have made appropriate decisions to share resources with other VPT members. The proposed evaluation method comprises a trust evaluation between two VPT workers and a reliability evaluation of worker. Each resource can have a threshold that is a benchmark for resource sharing. The trust evaluation method for the proposed VPT model enables secure resource sharing across team and enterprise boundaries, facilitates collaboration, and enhances information transparency among team members.

**EXCLUDE (This study looks at giving the responsibility to an individual representative of making decisions on behalf of a group or organization. This is exactly the opposite idea behind implementing a CCC. - 7)** Song, F.
Trust and reciprocity behavior and behavioral forecasts: Individuals versus group-representatives
(2008) Games and Economic Behavior, 62 (2), pp. 675-696.
https://www.scopus.com/inward/record.uri?eid=2-s2.0-39149137257&doi=10.1016%2fj.geb.2007.06.002&partnerID=40&md5=b2b045d5773634938c8469dbc9e592cc
DOCUMENT TYPE: Article
SOURCE: Scopus

Individuals are often given the responsibility of making decisions on behalf of a group or an organization. However, little is known about preferences or behavior in such contexts. In an adapted trust game, I examine whether the perspectives and behavior of group-representatives differ from those of the same individuals in an analogous situation. Group-representatives are given the responsibility of unilaterally and privately making a decision on behalf of a three-person group. Results from both the main and the follow-up studies show that people trust less and reciprocate less as group-representatives, and thus demonstrate that the interindividual-intergroup discontinuity effect not only exists in group behavior but also in individual behavior of group-representatives. Moreover, results show that trusting behavior is driven by reciprocity expectations, while reciprocating behavior is not sensitive to the first mover's behavior, i.e., the level of trust experienced.

**EXCLUDE (This study focuses on virtual teams, is close to 10 years old, and doesn’t seem as applicable for CCCs as we have included above. - 7)** Clemmensen, T., Khryashcheva, M., Podshibikhina, O.
Combining bases of trust development in virtual teams
(2008) International Journal of Networking and Virtual Organisations, 5 (1), pp. 17-34.
https://www.scopus.com/inward/record.uri?eid=2-s2.0-37849041282&doi=10.1504%2fIJNVO.2008.015999&partnerID=40&md5=3646a7a992d573c57cb880da2f0f7c32
DOCUMENT TYPE: Article
SOURCE: Scopus

In the setting of a virtual team where cooperating team members are not physically co-located and where technology is used as the major means of communication, trust plays a vital role - the glue that holds the entire team together. We suggest that the key assumption in conceptualisation of trust is its dynamic nature, and analyse the formation and maintenance of trust. The purpose is to look holistically at a number of bases including calculative-, cognition-, value-norm-, affect-, and impersonal-based trust. Our conclusion is that most of the trust bases are important in making a decision to trust in the initial phases of the virtual group's life. However, as trust has a very dynamic nature, the bases of trust change their relative importance through time under the influence of people's interactions among themselves. We present a framework for further empirical research into trust formation and maintenance in virtual teams.

**EXCLUDE (This study focuses on distributed work teams (specifically engineers), is close to 10 years old, and doesn’t seem as applicable for CCCs as we have included above. - 7)** Julsrud, T.E.
Flows, bridges and brokers: Exploring the development of trust relations in a distributed work group
(2008) International Journal of Networking and Virtual Organisations, 5 (1), pp. 83-102.
https://www.scopus.com/inward/record.uri?eid=2-s2.0-37849017899&doi=10.1504%2fIJNVO.2008.016004&partnerID=40&md5=1f1836fa14d30207fc7cb9a3fdb78844
DOCUMENT TYPE: Article
SOURCE: Scopus

During the last few decades several important empirical studies have documented that trust is important for the efficiency of distributed groups (Piccoli and Ives, 2003; Wilson et al., 2006). It has also been documented that more task-oriented forms of trust (i.e., swift trust) develop more easily in such teams than affective trust forms (Meyerson et al., 1996; Jarvenpaa and Leidner, 1999; Kanawattanachai and Yoo, 2002). More poorly understood are the underlying mechanisms that generate different types of trust within distributed groups in the first place. In this article, findings from a study of affective and cognitive trust relations in a group of distributed engineers are presented, and it is demonstrated how these trust forms followed slightly different patterns. The findings indicate that 'trust brokering' occurred along both dimensions and that these activities were crucial for the development of trust in the group.

**EXCLUDE (The two-person trust game discussed in this study does not really align with CCCs - 7)** Kugler, T., Bornstein, G., Kocher, M.G., Sutter, M.
Trust between individuals and groups: Groups are less trusting than individuals but just as trustworthy
(2007) Journal of Economic Psychology, 28 (6), pp. 646-657.
https://www.scopus.com/inward/record.uri?eid=2-s2.0-33947589561&doi=10.1016%2fj.joep.2006.12.003&partnerID=40&md5=52768d388a56ea729276ee455518a22a
DOCUMENT TYPE: Article
SOURCE: Scopus

We compare the behavior of groups and individuals in a two-person trust game. The first mover in this game, the sender, receives an endowment and can send any part of it to the responder; the amount sent is tripled, and the responder can then return to the sender any portion of the tripled sum. In a 2 × 2 design, the players in the roles of sender and responder are either individuals or groups of three players (who conduct face-to-face discussions to decide on a collective group strategy). We find that groups in the role of sender send smaller amounts than individuals, and expect lower returns. In particular, groups send nothing more often than individuals do (and are even more likely to do so when the responder is another group). Groups and individuals in the role of responder return on average the same fraction of the amount sent. Hence, we conclude that groups are less trusting than individuals, but just as trustworthy.

**INCLUDE IN ARTICLE REVIEW (coded)** Parayitam, S., Dooley, R.S.
The relationship between conflict and decision outcomes: Moderating effects of cognitive- and affect-based trust in strategic decision-making teams
(2007) International Journal of Conflict Management, 18 (1), pp. 42-73.
https://www.scopus.com/inward/record.uri?eid=2-s2.0-34547448836&doi=10.1108%2f10444060710759318&partnerID=40&md5=5d67c38f3761e36ad099a658d5df3684
DOCUMENT TYPE: Article
SOURCE: Scopus

Purpose - Research on strategic decision making has over-emphasized the importance of cognitive conflict because of its potential benefits. Recent research documented that, apart from the benefits, cognitive conflict leads to affective conflict. Taking information processing perspective, the present study seeks to argue that the benefits of cognitive conflict can be stimulated by the cognition-based trust, while the interplay between cognitive conflict and affective conflict can be influenced by affect-based trust. The present study therefore aims to demonstrate the divergent roles of the perceived trustworthiness as potential moderators in strategic decision-making teams. Design/methodology/approach - Using structured survey instrument, multi-informant data was collected from CEOs and senior executives of 109 US hospitals. After performing confirmatory factor analysis of the measures used, the data was analyzed using hierarchical regression techniques to analyze divergent roles of cognition- and affect-based trust as moderators in the relationship between conflict and decision outcomes. Findings - Results showed that cognition-based trust is the key to fortify the benefits of cognitive conflict while affect-based trust is the panacea for the ills of cognitive conflict. Research limitations/implications - The sample consisted of hospitals in healthcare industry only. Self-report measures may have some inherent social desirability bias. Practical implications - This study contributes to both practicing managers as well as to strategic management literature. This study suggests that trust between the executives involved in strategic decision-making process plays an important role in enhancing decision quality. It is therefore suggested that CEOs and administrators engage the executives who have both cognition- and affect-based trust with each other to have successful decision outcomes. Originality/value - Though the sample in the present study focuses only on healthcare industry, to the extent strategic decision-making process is similar in other industries, the findings can be generalizable across other industries.

**EXCLUDE (This study focuses on virtual project teams, is over 10 years old, and doesn’t seem as applicable for CCCs as we have included above. - 7)** Greenberg, P.S., Greenberg, R.H., Antonucci, Y.L.
Creating and sustaining trust in virtual teams
(2007) Business Horizons, 50 (4), pp. 325-333.
https://www.scopus.com/inward/record.uri?eid=2-s2.0-34249025850&doi=10.1016%2fj.bushor.2007.02.005&partnerID=40&md5=1cbe90bb757bdd4a6deb62947ee0c07f
DOCUMENT TYPE: Article
SOURCE: Scopus

Conventional wisdom assumes that trust develops from a history of interpersonal interactions and communication, through which people come to 'know and trust' one another. In virtual teams, however, establishing trust can be complicated: members may have no past on which to build, no future to reference, and may never even actually meet face-to-face. Swift but fragile trust can develop early in a team's life cycle. Yet, if swift trust doesn't develop or even dissipates, members need to find ways of building trust in each other. To this end, an understanding of how trust impacts a virtual team's development will help managers and team leaders to facilitate and improve team success. Herein, we describe the three components of trust (ability, integrity, and benevolence) and identify which of these are critical to each life cycle stage (establishing the team, inception, organizing, transition, and accomplishing the task) of the virtual team. Proposed action steps for each stage show managers and team leaders how to help members develop trust and sustain it through the project's successful completion.

**EXCLUDE (Although interesting, this study seems to be focused more on a learning or performance-based environment than a group trying to make collective decisions and not evaluating individual performance of other faculty members. - 4)** Molleman, E., Nauta, A., Buunk, B.P.
Social comparison-based thoughts in groups: Their associations with interpersonal trust and learning outcomes
(2007) Journal of Applied Social Psychology, 37 (6), pp. 1163-1180.
https://www.scopus.com/inward/record.uri?eid=2-s2.0-34248596322&doi=10.1111%2fj.1559-1816.2007.00207.x&partnerID=40&md5=ba86e77ad1d011a6ff105393b600fc49
DOCUMENT TYPE: Article
SOURCE: Scopus

This study relates thoughts derived from 4 types of social comparison to trust and individual learning. Our study (N = 362 students) showed that upward identification (i.e., believing one is just as good as a better performing teammate) was positively related to trust and individual learning. Upward contrast (i.e., believing one is worse than a better performing group member) was negatively related to learning, as were downward-identifying thoughts (i.e., believing one will perform as badly as a poorly performing teammate). Downward contrast (i.e., thinking one can do much better than the poor performer) was negatively related to trust. We concluded that social comparison-based thoughts are important to consider when designing teamwork because of their constructive and destructive consequences.

**EXCLUDE (No abstract available, and the title implies the focus only on team collaboration and healthy work environments, not on decisions or team outcomes - 7)** Reina, M.L., Reina, D.S., Rushton, C.H.
Trust: The foundation for team collaboration and healthy work environments
(2007) AACN Advanced Critical Care, 18 (2), pp. 103-108.
https://www.scopus.com/inward/record.uri?eid=2-s2.0-34250683478&doi=10.1097%2f01.AACN.0000269252.94224.0b&partnerID=40&md5=47c80eec37e63871c25d92fc66faf9a1
DOCUMENT TYPE: Review
SOURCE: Scopus

No abstract available

**EXCLUDE (This abstract was reviewed on the PUBMED document)** McKinstry, B., Ashcroft, R.E., Car, J., Freeman, G.K., Sheikh, A.
Interventions for improving patients' trust in doctors and groups of doctors.
(2006) Cochrane database of systematic reviews (Online), 3, pp. CD004134.
https://www.scopus.com/inward/record.uri?eid=2-s2.0-33750136809&partnerID=40&md5=66b17558b4c0c51092d44f7aee9499cd
DOCUMENT TYPE: Review
SOURCE: Scopus

**EXCLUDE (This is an article from an economics journal, which typically don’t have very much applicability to CCCs. Also the idea of making group decisions by a single representative, inter-group interactions, and the idea of reciprocity really doesn’t fit well with a CCC - 7)** Song, F.
Trust and reciprocity in inter-individual versus inter-group interactions: The effects of social influence, group dynamics, and perspective biases
(2006) Experimental Economics, 9 (2), pp. 179-180.
https://www.scopus.com/inward/record.uri?eid=2-s2.0-33744815962&doi=10.1007%2fs10683-006-7051-x&partnerID=40&md5=600bacf3e22ee8f2d71d10573c24280d
DOCUMENT TYPE: Article
SOURCE: Scopus

Using an experimental trust game, I examine whether the perspectives and behavior of group representatives and consensus groups differ from those of the same individuals in an analogous inter-individual situation. A primary goal of this research is to extend past work on trust and reciprocity by examining the impact of the social contexts within which social interactions are characteristically embedded. Specifically, this research concerns whether norms and dynamics of trust and reciprocity differ in the contexts of inter-individual and inter-group interactions. First, I examine whether dynamics of trust and reciprocity differ in various inter-group interactions where inter-group decisions are operationalized as 1) autonomous group representatives, i.e., individuals who are given the responsibility of unilaterally making a decision on behalf of a three-person group engaging with a group representative of another such group; and 2) consensus groups, i.e., group members making a consensus trust or reciprocity decision for their groups via a collective process with another such group. Results of these studies show that 1) people trust less and reciprocate less when responsible for a group or organizational decision as autonomous group representatives; 2) consensus groups do not differ from individuals in their level of trust but show dramatically less reciprocity. The group consensus mechanism in fact produced by far the lowest reciprocity level, significantly lower than that exhibited by either individuals or autonomous group representatives. Thus, inter-group trust and reciprocity dynamics are not readily inferable from their inter-individual counterparts. Moreover, an important implication is emerging here: the extent and direction of the discrepancy between individual and group choices in regard to trust and reciprocity levels and possibly other social preferences in general may depend importantly on the precise details of the group decision-making mechanism, for example whether decisions are made consensually, by majority vote, or by a group leader or representative. In addition to examining the level of trust and reciprocity that occur in these various situations, I also studied, using both behavioral and questionnaire data, the roles of self-interest, social influence, and group dynamics in trust and reciprocity perceptions and behavior. The results showed that there exist discrepancies between behavioral forecasts and the actual behavior, and that trusting behavior is driven strongly by expectation of level of reciprocation, while reciprocating behavior is driven strongly by the difference between trust expectation and actual trust received.

**EXCLUDE (This article focuses on interpersonal perceptions of trustworthiness or a shared group membership, it doesn’t really discuss actions of the group per se that make it more or less trustworthy. I don’t feel this is very applicable to our study - 4)** Tanis, M., Postmes, T.
Short communication a social identity approach to trust: Interpersonal perception, group membership and trusting behaviour
(2005) European Journal of Social Psychology, 35 (3), pp. 413-424.
https://www.scopus.com/inward/record.uri?eid=2-s2.0-20444475443&doi=10.1002%2fejsp.256&partnerID=40&md5=ebae91f17f66b6fe05d6fbc2e5091acf
DOCUMENT TYPE: Article
SOURCE: Scopus

Trusting behaviour involves relinquishing control over outcomes valuable to the self. Previous research suggests that interpersonal perceptions of trustworthiness are closely related to this behaviour. The present research suggests that the more proximal determinant of trusting behaviour is the expectation that the other will reciprocate. Based on the Social Identity model of Deindividuation Effects (SIDE) model, reciprocity expectations may be created by interpersonal perceptions of trustworthiness or a shared group membership. To investigate this, group membership and individual identifiability were experimentally manipulated (N = 139): When individuals were not identifiable, trusting behaviour was based on expectations of reciprocity inferred from group membership, not on perceived trustworthiness. In contrast, personal identifiability fostered perceptions of trustworthiness for both in- and out-group members. In this case interpersonal trustworthiness enhanced expectations of reciprocity, which in turn increased trusting behaviour.

**EXCLUDE (This article focuses on trust in online education and is 13 years old. I don’t feel this is as applicable to CCC as the articles for virtual teams above. - 7)** Wojnar, L., Uden, L.
Group Process & Trust in Group Discussion
(2005) International Journal of Information and Communication Technology Education (IJICTE), 1 (1), pp. 55-68.
https://www.scopus.com/inward/record.uri?eid=2-s2.0-85001600709&doi=10.4018%2fjicte.2005010106&partnerID=40&md5=901c0a3e4b8cbf8ae72d56d49ded0ee2
DOCUMENT TYPE: Article
SOURCE: Scopus

Successful group discussion plays a crucial role in online learning. Teachers normally assume that students automatically transfer their learning of group process from group to group. Our experience found that for group discussion to be effective, it is important that we consider group process and the role of trust within groups. The article begins with an introduction to group process and trust followed by a brief review of the benefits of group discussion for online learning. In the third section, we describe the role that teachers play in initiating environments that promote trust and group empowerment. Through our experience of the implications of group process and trust, we will discuss how this environment fosters trusting relationships. We will also discuss the value of reviewing the group process for each class before assigning group work. The article concludes with the outcomes of our experience and suggestions for further work.

**EXCLUDE (This article focuses on trust in online education and is 14 years old. I don’t feel this is as applicable to CCC as the articles for virtual teams above. - 7)** Coppola, N.W., Hiltz, S.R., Rotter, N.G.
Building trust in virtual teams
(2004) IEEE Transactions on Professional Communication, 47 (2), pp. 95-104.
https://www.scopus.com/inward/record.uri?eid=2-s2.0-3042589339&doi=10.1109%2fTPC.2004.828203&partnerID=40&md5=e512d04fbd2ce09f3bf7bf9e253f769f
DOCUMENT TYPE: Article
SOURCE: Scopus

This paper presents a study of trust development in online courses. It reviews the concept of swift trust and examines changes in faculty roles as professors go online. An exploratory content analysis looks at indicators of the development of swift trust in the highest rated of a large number of online courses studied over a three-year period, and contrasts these results with one of the poorest-rated online courses. Establishing swift trust at the beginning of an online course appears to be related to subsequent course success. Strategies for trust formation are also suggested.

**EXCLUDE (This article focuses on trust in global virtual teams and is 14 years old. I don’t feel this is as applicable to CCC as the articles for virtual teams above. - 7)**Jarvenpaa, S.L., Shaw, T.R., Staples, D.S.
Toward contextualized theories of trust: The role of trust in global virtual teams
(2004) Information Systems Research, 15 (3), pp. 250-267.
https://www.scopus.com/inward/record.uri?eid=2-s2.0-9144229160&doi=10.1287%2fisre.1040.0028&partnerID=40&md5=b60cf10b2d505ae848a8b54fa3be7af8
DOCUMENT TYPE: Review
SOURCE: Scopus

Although trust has received much attention in many streams of information systems research, there has been little theorizing to explain how trust evokes sentiments and affects task performance in IT-enabled relationships. Many studies unquestionably assume that trust is intrinsically beneficial, and dismiss the possibility that the effects of trust may be dependent on the situation (or conditions) at present. This paper theoretically and empirically examines outcomes of an individual's trust in global virtual teams under differing situations (or conditions). In Study 1, we find that early in a team's existence, a member's trusting beliefs have a direct positive effect on his or her trust in the team and perceptions of team cohesiveness. Later on, however, a member's trust in his team operates as a moderator, indirectly affecting the relationships between team communication and perceptual outcomes. Study 2 similarly suggests that trust effects are sensitive to the particular situation or condition. Combined, the studies find that trust affects virtual teams differently in different situations. Future studies on trust will need to consider situational contingencies. This paper contributes to the literature on IT-enabled relationships by theorizing and empirically testing how trust affects attitudes and behaviors.

**EXCLUDE (Although this article is interesting, we’re not really looking at mixed teams from different sections of the organization. I think multiple other papers suggest that diversity can hinder group cohesion and trust. - 2)** Meeting the challenges of cross-functional teams: The key role of creating a climate of trust
(2003) Human Resource Management International Digest, 11 (3), pp. 34-36.
https://www.scopus.com/inward/record.uri?eid=2-s2.0-84986064539&doi=10.1108%2f09670730310792727&partnerID=40&md5=94c319eee60284b128753a48bae6bbb3
DOCUMENT TYPE: Article
SOURCE: Scopus

A team made up of employees from different sections of an organization can be a great recipe for success, but its full potential can only be achieved through skilled leaders creating an atmosphere of mutual trust. Mixed teams face various challenges, including differences in personality, culture, language or jargon, and reward systems. Members may have diverse backgrounds, experiences, values and beliefs, and this diversity can hinder group cohesion and handicap social interaction.

**INCLUDE IN ARTICLE REVIEW (coded)** Simsarian Webber, S.
Leadership and trust facilitating cross-functional team success
(2002) Journal of Management Development, 21 (3), pp. 201-214.
https://www.scopus.com/inward/record.uri?eid=2-s2.0-84986178170&doi=10.1108%2f02621710210420273&partnerID=40&md5=1b7d069c11ed41617cb67ff768db293b
DOCUMENT TYPE: Article
SOURCE: Scopus

Cross-functional teams (CFTs) have increased in use within a variety of organizations. While these teams claim to enhance organizational effectiveness, research has seen mixed results. This paper examines the challenges faced by CFTs and why these challenges facilitate the need for the development of a team climate for trust. Trust is discussed as a team-level construct, an aspect of the “micro-climate” that occurs within a team. Leadership actions particularly important to cross-functional teams and the development of trust are offered as influential in creating a team climate for trust in cross-functional teams.

**INCLUDE IN ARTICLE REVIEW (This abstract was reviewed on the PUBMED sheet)** Simons, T.L., Peterson, R.S.
Task conflict and relationship conflict in top management teams: The pivotal role of intragroup trust
(2000) Journal of Applied Psychology, 85 (1), pp. 102-111.
https://www.scopus.com/inward/record.uri?eid=2-s2.0-0034131549&doi=10.1037%2f0021-9010.85.1.102&partnerID=40&md5=9fc30a0c9ce9b05e1cbc29f072c7d24b
DOCUMENT TYPE: Article
SOURCE: Scopus

**EXCLUDE (The title of the article, journal in which it is published, and the age of the article (along with no abstract) suggests minimal applicability to CCCs - 7)** Thiagarajan, S.
Easy money: An exploration of trust in teams
(1997) Simulation and Gaming, 28 (2), pp. 238-241.
https://www.scopus.com/inward/record.uri?eid=2-s2.0-79959553242&doi=10.1177%2f1046878197282008&partnerID=40&md5=19d651fae77cac6933d59ca6a4ccb399
DOCUMENT TYPE: Article
SOURCE: Scopus

No abstract available

**Exclude (these articles have already been excluded based upon the title review):**

**EXCLUDE (computer science focus; not applicable or readily transferable to CCCs - 6)**

Wu, J., Dai, L., Chiclana, F., Fujita, H., Herrera-Viedma, E.
A minimum adjustment cost feedback mechanism based consensus model for group decision making under social network with distributed linguistic trust
(2018) Information Fusion, 41, pp. 232-242.
https://www.scopus.com/inward/record.uri?eid=2-s2.0-85029590284&doi=10.1016%2fj.inffus.2017.09.012&partnerID=40&md5=a2635a012b4136085323a5c781edf16c
DOCUMENT TYPE: Article
SOURCE: Scopus

**EXCLUDE (politics; not applicable or readily transferable to CCCs - 3)**

Balliet, D., Tybur, J.M., Wu, J., Antonellis, C., Van Lange, P.A.M.
Political Ideology, Trust, and Cooperation: In-group Favoritism among Republicans and Democrats during a US National Election
(2018) Journal of Conflict Resolution, 62 (4), pp. 797-818.
https://www.scopus.com/inward/record.uri?eid=2-s2.0-85014809916&doi=10.1177%2f0022002716658694&partnerID=40&md5=d6dda15fdca29f3bbf9c3d567335dcdd
DOCUMENT TYPE: Article
SOURCE: Scopus

**EXCLUDE (legal focus; not applicable or readily transferable to CCCs - 3)**

Manzi, L., Hall, M.E.K.
Friends You Can Trust: A Signaling Theory of Interest Group Litigation Before the U.S. Supreme Court
(2017) Law and Society Review, 51 (3), pp. 704-734.
https://www.scopus.com/inward/record.uri?eid=2-s2.0-85027333955&doi=10.1111%2flasr.12280&partnerID=40&md5=fad9d00d18f4a0fb40bb919a669b85fb
DOCUMENT TYPE: Article
SOURCE: Scopus

**EXCLUDE (computer science focus; not applicable or readily transferable to CCCs - 6)** Krishankumar, R., Ravichandran, K.S.
Realizing the effects of trust and personality in cross functional teams using ANFIS classification framework
(2017) Computational and Mathematical Organization Theory, pp. 1-34. Article in Press.
https://www.scopus.com/inward/record.uri?eid=2-s2.0-85021816475&doi=10.1007%2fs10588-017-9256-2&partnerID=40&md5=46fb977052116e7f9c8d203152ccc597
DOCUMENT TYPE: Article in Press
SOURCE: Scopus

**EXCLUDE (subject/population studied; not applicable or readily transferable to CCCs - 3)**

Ye, J., Ng, S.H.
An intermediary enhances out-group trust and in-group profit expectation of Chinese but not Australians
(2017) International Journal of Psychology, 52 (3), pp. 189-196.
https://www.scopus.com/inward/record.uri?eid=2-s2.0-84938152467&doi=10.1002%2fijop.12199&partnerID=40&md5=df837a90955ecc6e4b5a0a1db6d06102
DOCUMENT TYPE: Article
SOURCE: Scopus

**EXCLUDE (production techology? Principal-agent relationships?; not applicable or readily transferable to CCCs - 3)**

Cobo-Reyes, R., Lacomba, J.A., Lagos, F., Levin, D.
The effect of production technology on trust and reciprocity in principal-agent relationships with team production
(2017) Journal of Economic Behavior and Organization, 137, pp. 324-338.
https://www.scopus.com/inward/record.uri?eid=2-s2.0-85017166340&doi=10.1016%2fj.jebo.2017.03.008&partnerID=40&md5=0f85601df4519b9ad8512749c2867b3d
DOCUMENT TYPE: Article
SOURCE: Scopus

**EXCLUDE (I’ve reviewed this article, computer science emphasis; not applicable or readily transferable to CCCs - 6)**

Wu, J., Chiclana, F., Fujita, H., Herrera-Viedma, E.
A visual interaction consensus model for social network group decision making with trust propagation
(2017) Knowledge-Based Systems, 122, pp. 39-50.
https://www.scopus.com/inward/record.uri?eid=2-s2.0-85011271331&doi=10.1016%2fj.knosys.2017.01.031&partnerID=40&md5=8c95a53a0e9fe292169472183a045935
DOCUMENT TYPE: Article
SOURCE: Scopus

**EXCLUDE (hormones/biochemistry; not applicable or readily transferable to CCCs - 1)**

Kret, M.E., De Dreu, C.K.W.
Pupil-mimicry conditions trust in partners: Moderation by oxytocin and group membership
(2017) Proceedings of the Royal Society B: Biological Sciences, 284 (1850), art. no. 20162554,.
https://www.scopus.com/inward/record.uri?eid=2-s2.0-85014546028&doi=10.1098%2frspb.2016.2554&partnerID=40&md5=5323e8fd71df050a30e0890f8bc1d373
DOCUMENT TYPE: Article
SOURCE: Scopus

**EXCLUDE (hormones/biochemistry; not applicable or readily transferable to CCCs - 4)**

De Visser, E.J., Monfort, S.S., Goodyear, K., Lu, L., O'Hara, M., Lee, M.R., Parasuraman, R., Krueger, F.
A Little Anthropomorphism Goes a Long Way: Effects of Oxytocin on Trust, Compliance, and Team Performance with Automated Agents
(2017) Human Factors, 59 (1), pp. 116-133.
https://www.scopus.com/inward/record.uri?eid=2-s2.0-85011565227&doi=10.1177%2f0018720816687205&partnerID=40&md5=767dc49416732d7b700b79a662e6ed7f
DOCUMENT TYPE: Article
SOURCE: Scopus

**EXCLUDE (child development; not applicable or readily transferable to CCCs - 3)**

Grütter, J., Gasser, L., Zuffianò, A., Meyer, B.
Promoting Inclusion Via Cross-Group Friendship: The Mediating Role of Change in Trust and Sympathy
(2017) Child Development, . Article in Press.
https://www.scopus.com/inward/record.uri?eid=2-s2.0-85020543862&doi=10.1111%2fcdev.12883&partnerID=40&md5=4ba27941a5db7f13c4923d19712b2c47
DOCUMENT TYPE: Article in Press
SOURCE: Scopus

**EXCLUDE (computer science focus; not applicable or readily transferable to CCCs - 6)**

Wan, S.-P., Xu, J.
A method for multi-attribute group decision-making with triangular intuitionistic fuzzy numbers application to trustworthy service selection
(2017) Scientia Iranica, 24 (2), pp. 794-807.
https://www.scopus.com/inward/record.uri?eid=2-s2.0-85019120610&partnerID=40&md5=a36ea32c63a94fe5b0bfd231cd530255
DOCUMENT TYPE: Article
SOURCE: Scopus

**EXCLUDE (substance abuse; not applicable or readily transferable to CCCs - 3)**

Silverman, M.J.
Effects of Live and Educational Music Therapy on Working Alliance and Trust With Patients on Detoxification Unit: A Four-Group Cluster-Randomized Trial
(2016) Substance Use and Misuse, 51 (13), pp. 1741-1750.
https://www.scopus.com/inward/record.uri?eid=2-s2.0-84980401128&doi=10.1080%2f10826084.2016.1197263&partnerID=40&md5=72bf31d3562371d122fe9d1f696becfe
DOCUMENT TYPE: Article
SOURCE: Scopus

**EXCLUDE (ethics, population study; not applicable or readily transferable to CCCs - 3)** Romain, F., Courtwright, A.
Can I trust them to do everything? The role of distrust in ethics committee consultations for conflict over life-sustaining treatment among Afro-Caribbean patients
(2016) Journal of Medical Ethics, 42 (9), pp. 582-585.
https://www.scopus.com/inward/record.uri?eid=2-s2.0-84969204372&doi=10.1136%2fmedethics-2015-103137&partnerID=40&md5=84627fe3e984478f3b4ae1cd5c2c3efd
DOCUMENT TYPE: Article
SOURCE: Scopus

**EXCLUDE (computer science emphasis; not applicable or readily transferable to CCCs - 6)** Xu, X., Wang, B., Zhou, Y.
A method based on trust model for large group decision-making with incomplete preference information
(2016) Journal of Intelligent and Fuzzy Systems, 30 (6), pp. 3551-3565.
https://www.scopus.com/inward/record.uri?eid=2-s2.0-84971475298&doi=10.3233%2fIFS-162100&partnerID=40&md5=5dcc1484ff8b361fd769687dbc8c737e
DOCUMENT TYPE: Article
SOURCE: Scopus

**EXCLUDE (computer science emphasis; not applicable or readily transferable to CCCs - 6)** Wu, J., Xiong, R., Chiclana, F.
Uninorm trust propagation and aggregation methods for group decision making in social network with four tuple information
(2016) Knowledge-Based Systems, 96, pp. 29-39.
https://www.scopus.com/inward/record.uri?eid=2-s2.0-84957882020&doi=10.1016%2fj.knosys.2016.01.004&partnerID=40&md5=3ef5542f3f45a122a7b3113f8980065c
DOCUMENT TYPE: Article
SOURCE: Scopus

**EXCLUDE (computer science focus; not applicable or readily transferable to CCCs - 6)** Nagaraja, G., Pradeep Reddy, C.H.
Mitigate lying and on-off attacks on trust based group key management frameworks in MANETs
(2016) International Journal of Intelligent Engineering and Systems, 9 (4), pp. 215-224.
https://www.scopus.com/inward/record.uri?eid=2-s2.0-85006088627&doi=10.22266%2fijies2016.1231.23&partnerID=40&md5=18dd230b280adf29b27038ae9eb3bf72
DOCUMENT TYPE: Article
SOURCE: Scopus

**EXCLUDE (population studied; not applicable or readily transferable to CCCs - 4)**

Lossie, A.C., Green, J.
Building trust: The history and ongoing relationships amongst DSD clinicians, researchers, and patient advocacy groups
(2015) Hormone and Metabolic Research, 47 (5), pp. 344-350.
https://www.scopus.com/inward/record.uri?eid=2-s2.0-84937758051&doi=10.1055%2fs-0035-1548793&partnerID=40&md5=48d4817ffb0d82a5063ffb904335f014
DOCUMENT TYPE: Article
SOURCE: Scopus

**EXCLUDE (computer science emphasis/online group-buying?; not applicable or readily transferable to CCCs - 6)**

Wang, W.-T., Wang, Y.-S., Liu, E.-R.
The stickiness intention of group-buying websites: The integration of the commitment-trust theory and e-commerce success model
(2015) Information and Management, . Article in Press.
https://www.scopus.com/inward/record.uri?eid=2-s2.0-84959220728&doi=10.1016%2fj.im.2016.01.006&partnerID=40&md5=eecc61e8a3ec2e79a83a83a290a6438c
DOCUMENT TYPE: Article in Press
SOURCE: Scopus

**EXCLUDE (computer science focus; not applicable or readily transferable to CCCs - 6)**

De Meo, P., Ferrara, E., Rosaci, D., Sarné, G.M.L.
Trust and compactness in social network groups
(2015) IEEE Transactions on Cybernetics, 45 (2), art. no. 6869022, pp. 205-216.
https://www.scopus.com/inward/record.uri?eid=2-s2.0-85027952562&doi=10.1109%2fTCYB.2014.2323892&partnerID=40&md5=8405fc41459565484a16a9a5fd0b59c2
DOCUMENT TYPE: Article
SOURCE: Scopus

**EXCLUDE (subject/population studied; not applicable or readily transferable to CCCs - 4)**

Su, W., Liao, Y.
A jury-based trust management mechanism in distributed cognitive radio networks
(2015) China Communications, 12 (7), art. no. 7188530, pp. 119-126.
https://www.scopus.com/inward/record.uri?eid=2-s2.0-84939530263&doi=10.1109%2fCC.2015.7188530&partnerID=40&md5=e2cbbc262d92a6aba64a7c2b91cfec7d
DOCUMENT TYPE: Article
SOURCE: Scopus

**EXCLUDE (ropes course?; not applicable or readily transferable to CCCs - 2)**

Eatough, E., Chang, C.-H., Hall, N.
Getting roped in: Group cohesion, trust, and efficacy following a ropes course intervention
(2015) Performance Improvement Quarterly, 28 (2), pp. 65-89.
https://www.scopus.com/inward/record.uri?eid=2-s2.0-84937716982&doi=10.1002%2fpiq.21183&partnerID=40&md5=aaed88512dab65c3f29304b69d210a2a
DOCUMENT TYPE: Article
SOURCE: Scopus

**EXCLUDE (subject/population studied; not applicable or readily transferable to CCCs - 3)** Han, H., Hwang, J.
Quality of physical surroundings and service encounters, airfare, trust and intention during the flight: Age-group difference (young, middle-aged, and mature)
(2015) International Journal of Contemporary Hospitality Management, 27 (4), pp. 585-607.
https://www.scopus.com/inward/record.uri?eid=2-s2.0-84929258149&doi=10.1108%2fIJCHM-08-2013-0344&partnerID=40&md5=ff9d14a1f96148e12c3823ee24f0cb8e
DOCUMENT TYPE: Article
SOURCE: Scopus

**EXCLUDE (subject/population studied; not applicable or readily transferable to CCCs - 3)** Silverman, M.J.
Effects of a live educational music therapy intervention on acute psychiatric inpatients' perceived social support and trust in the therapist: A four-group randomized effectiveness study
(2015) Journal of Music Therapy, 51 (3), pp. 228-249.
https://www.scopus.com/inward/record.uri?eid=2-s2.0-84929030807&doi=10.1093%2fjmt%2fthu011&partnerID=40&md5=8fc611496e3158fc1909a6360bd2b4b0
DOCUMENT TYPE: Article
SOURCE: Scopus

**EXCLUDE (computer science focus; not applicable or readily transferable to CCCs - 6)**

Li, X., Tian, Q., Yan, H., Li, X.
The trustworthiness of reference group and cooperative quality: Implications for online crowdsourcing market
(2015) International Journal of Networking and Virtual Organisations, 15 (2-3), pp. 242-255.
https://www.scopus.com/inward/record.uri?eid=2-s2.0-84936758007&doi=10.1504%2fIJNVO.2015.070434&partnerID=40&md5=1b0929b99a4a9bf488a8f06e6b497e03
DOCUMENT TYPE: Article
SOURCE: Scopus

**EXCLUDE (subject/population studied; not applicable or readily transferable to CCCs - 6)** Weng, W., Yang, F.
The impact of social identity on trust in China: Experimental evidence from cross-group comparisons
(2014) Applied Economics, 46 (16), pp. 1855-1860.
https://www.scopus.com/inward/record.uri?eid=2-s2.0-84896868057&doi=10.1080%2f00036846.2014.887196&partnerID=40&md5=b00f707ab32c5ca91931bf890242c982
DOCUMENT TYPE: Article
SOURCE: Scopus

**EXCLUDE (computer science focus; not applicable or readily transferable to CCCs - 6)**

Wu, J., Chiclana, F.
A social network analysis trust-consensus based approach to group decision-making problems with interval-valued fuzzy reciprocal preference relations
(2014) Knowledge-Based Systems, 59, pp. 97-107.
https://www.scopus.com/inward/record.uri?eid=2-s2.0-84897660200&doi=10.1016%2fj.knosys.2014.01.017&partnerID=40&md5=cd483623f4d56e41ba1bddf2a518f4bf
DOCUMENT TYPE: Article
SOURCE: Scopus

**EXCLUDE (subject/population studied; not applicable or readily transferable to CCCs - 2)** Tenzer, H., Pudelko, M., Harzing, A.-W.
The impact of language barriers on trust formation in multinational teams
(2014) Journal of International Business Studies, 45 (5), pp. 508-535.
https://www.scopus.com/inward/record.uri?eid=2-s2.0-84902204689&doi=10.1057%2fjibs.2013.64&partnerID=40&md5=604f2137d5f789f25ba45c6c3756d6eb
DOCUMENT TYPE: Article
SOURCE: Scopus

**EXCLUDE (subject/population studied; not applicable or readily transferable to CCCs - 2)** Cater, J.J., III, Kidwell, R.E.
Function, governance, and trust in successor leadership groups in family firms
(2014) Journal of Family Business Strategy, 5 (3), pp. 217-228.
https://www.scopus.com/inward/record.uri?eid=2-s2.0-84908066721&doi=10.1016%2fj.jfbs.2013.06.001&partnerID=40&md5=a8bedb5fc4be8049f24429078e4d92e2
DOCUMENT TYPE: Article
SOURCE: Scopus

**EXCLUDE (child focus; not applicable or readily transferable to CCCs - 3)**

Barth, H., Bhandari, K., Garcia, J., MacDonald, K., Chase, E.
Preschoolers trust novel members of accurate speakers' groups and judge them favourably
(2014) Quarterly Journal of Experimental Psychology, 67 (5), pp. 872-883.
https://www.scopus.com/inward/record.uri?eid=2-s2.0-84899941387&doi=10.1080%2f17470218.2013.836234&partnerID=40&md5=824c4aa60d58b94859c7c3f7216e0f71
DOCUMENT TYPE: Article
SOURCE: Scopus

**EXCLUDE (child psychology; not applicable or readily transferable to CCCs - 4)**

Elashi, F.B., Mills, C.M.
Do children trust based on group membership or prior accuracy? The role of novel group membership in children's trust decisions
(2014) Journal of Experimental Child Psychology, 128, pp. 88-104.
https://www.scopus.com/inward/record.uri?eid=2-s2.0-84907357425&doi=10.1016%2fj.jecp.2014.07.003&partnerID=40&md5=bcb0ef97319d3ea4c11d6916d3439747
DOCUMENT TYPE: Article
SOURCE: Scopus

**EXCLUDE (computer science emphasis/online group-buying?; not applicable or readily transferable to CCCs - 6)**

Hsu, M.-H., Chang, C.-M., Chu, K.-K., Lee, Y.-J.
Determinants of repurchase intention in online group-buying: The perspectives of DeLone & McLean is success model and trust
(2014) Computers in Human Behavior, 36, pp. 234-245.
https://www.scopus.com/inward/record.uri?eid=2-s2.0-84899434414&doi=10.1016%2fj.chb.2014.03.065&partnerID=40&md5=8d535c1940cdd463705ce350f94776fc
DOCUMENT TYPE: Article
SOURCE: Scopus

**EXCLUDE (computer science focus; not applicable or readily transferable to CCCs - 6)**

Ming, X.
The research of P2P network security model based on group trust relationship
(2014) Journal of Chemical and Pharmaceutical Research, 6 (7), pp. 1103-1108.
https://www.scopus.com/inward/record.uri?eid=2-s2.0-84907249686&partnerID=40&md5=98b163570a12b8eaafab620129111dd1
DOCUMENT TYPE: Article
SOURCE: Scopus

**EXCLUDE (subject/population focus; not applicable or readily transferable to CCCs** - 3) Williams, W., Graham, D.P., McCurry, K., Sanders, A., Eiseman, J., Chiu, P.H., King-Casas, B.
Group psychotherapy's impact on trust in veterans with PTSD: A pilot study
(2014) Bulletin of the Menninger Clinic, 78 (4), pp. 335-348.
https://www.scopus.com/inward/record.uri?eid=2-s2.0-84920480743&doi=10.1521%2fbumc.2014.78.4.335&partnerID=40&md5=5bb7bba66b3d47a93009d4da48b6a6c3
DOCUMENT TYPE: Article
SOURCE: Scopus

**EXCLUDE (subject/population studied; not applicable or readily transferable to CCCs - 2)** Wai on, L., Liang, X., Priem, R., Shaffer, M.
Top management team trust, behavioral integration and the performance of international joint ventures
(2013) Journal of Asia Business Studies, 7 (2), pp. 99-122.
https://www.scopus.com/inward/record.uri?eid=2-s2.0-84962222053&doi=10.1108%2f15587891311319413&partnerID=40&md5=107bad8e4fe02a0984d719555e288610
DOCUMENT TYPE: Article
SOURCE: Scopus

**EXCLUDE (computer science focus; not applicable or readily transferable to CCCs - 6)**

Goh, J.C.-L., Pan, S.L., Zuo, M.
Developing the agile is development practices in large-scale it projects: The trust-mediated organizational controls and it project team capabilities perspectives
(2013) Journal of the Association of Information Systems, 14 (12), pp. 722-756.
https://www.scopus.com/inward/record.uri?eid=2-s2.0-84891397635&partnerID=40&md5=4a063594043e49688ec6d1b1f9f5052b
DOCUMENT TYPE: Article
SOURCE: Scopus

**EXCLUDE (repairing trust not the focus of this review - 2)**

Kim, P.H., Cooper, C.D., Dirks, K.T., Ferrin, D.L.
Repairing trust with individuals vs. groups
(2013) Organizational Behavior and Human Decision Processes, 120 (1), pp. 1-14.
https://www.scopus.com/inward/record.uri?eid=2-s2.0-84867295919&doi=10.1016%2fj.obhdp.2012.08.004&partnerID=40&md5=e60697ab18fd31929cc00fd6d40aae69
DOCUMENT TYPE: Article
SOURCE: Scopus

**EXCLUDE (subject/population studied; not applicable or readily transferable to CCCs - 4)** Pivetti, M., Montali, L., Simonetti, G.
The discourse around usefulness, morality, risk and trust: A focus group study on prenatal genetic testing
(2012) Prenatal Diagnosis, 32 (12), pp. 1205-1211.
https://www.scopus.com/inward/record.uri?eid=2-s2.0-84870053731&doi=10.1002%2fpd.3990&partnerID=40&md5=21e78320984ff334cce4e60ec63e5091
DOCUMENT TYPE: Article
SOURCE: Scopus

**EXCLUDE (online group-buying/e-commerce not the focus of this review - 6)**

Hsu, M.-H., Hsu, C.-S.
Understanding online group-buying intention: The role of trust, trust transference and conformity
(2012) Advances in Information Sciences and Service Sciences, 4 (12), pp. 37-45.
https://www.scopus.com/inward/record.uri?eid=2-s2.0-84864082490&doi=10.4156%2fAISS.vol4.issue12.5&partnerID=40&md5=f34042424dcca8705816c04fb74fa21e
DOCUMENT TYPE: Article
SOURCE: Scopus

**EXCLUDE (deception in virtual teams not the focus of this review - 6)** Fuller, C.M., Marett, K., Twitchell, D.P.
An examination of deception in virtual teams: Effects of deception on task performance, mutuality, and trust
(2012) IEEE Transactions on Professional Communication, 55 (1), art. no. 6092522, pp. 20-35.
https://www.scopus.com/inward/record.uri?eid=2-s2.0-84857355370&doi=10.1109%2fTPC.2011.2172731&partnerID=40&md5=395f4489baa977da570d9ed1e10421b0
DOCUMENT TYPE: Review
SOURCE: Scopus

**EXCLUDE (subject/population studied; not applicable or readily transferable to CCCs - 2)** Ling, F.Y.Y., Tran, H.B.T.
Ingredients to engender trust in construction project teams in Vietnam
(2012) Construction Innovation, 12 (1), pp. 43-61.
https://www.scopus.com/inward/record.uri?eid=2-s2.0-84855560301&doi=10.1108%2f14714171211197490&partnerID=40&md5=c4c9c1d675bbb93f09d45961c3882ab2
DOCUMENT TYPE: Article
SOURCE: Scopus

**EXCLUDE (geographically distributed teams and the development sector not the focus of our review - 6)**

Khan, M.S.
Role of trust and relationships in geographically distributed teams: Exploratory study on development sector
(2012) International Journal of Networking and Virtual Organisations, 10 (1), pp. 40-58.
https://www.scopus.com/inward/record.uri?eid=2-s2.0-84863399134&doi=10.1504%2fIJNVO.2012.045210&partnerID=40&md5=667a60e7e5a593fd1e33e8cef6e9c6ed
DOCUMENT TYPE: Article
SOURCE: Scopus

**EXCLUDE (race/reputation not the focus of our review; not applicable or readily transferable to CCCs - 1)**

Stanley, D.A., Sokol-Hessner, P., Fareri, D.S., Perino, M.T., Delgado, M.R., Banaji, M.R., Phelps, E.A.
Race and reputation: Perceived racial group trustworthiness influences the neural correlates of trust decisions
(2012) Philosophical Transactions of the Royal Society B: Biological Sciences, 367 (1589), pp. 744-753.
https://www.scopus.com/inward/record.uri?eid=2-s2.0-84856060936&doi=10.1098%2frstb.2011.0300&partnerID=40&md5=328d88f3c80756e0c005beff55377ddc
DOCUMENT TYPE: Article
SOURCE: Scopus

**EXCLUDE (computer science focus; not applicable or readily transferable to CCCs - 6)** Aikebaier, A., Enokido, T., Takizawa, M.
Trustworthy Group Making Algorithm in Distributed Systems
(2011) Human-centric Computing and Information Sciences, 1 (1), art. no. 6, pp. 1-15.
https://www.scopus.com/inward/record.uri?eid=2-s2.0-84958775075&doi=10.1186%2f2192-1962-1-6&partnerID=40&md5=c3e770301983b5e3883965b639f9df5d
DOCUMENT TYPE: Article
SOURCE: Scopus

**EXCLUDE (global virtual teams and social networks not the focus of our review - 6)**

Sarker, S., Ahuja, M., Sarker, S., Kirkeby, S.
The role of communication and trust in global virtual teams: A social network perspective
(2011) Journal of Management Information Systems, 28 (1), pp. 273-309.
https://www.scopus.com/inward/record.uri?eid=2-s2.0-80051711610&doi=10.2753%2fMIS0742-1222280109&partnerID=40&md5=758274d502add8581842b48082971d95
DOCUMENT TYPE: Review
SOURCE: Scopus

**EXCLUDE (technology adaptation in the setting of virtual teams not the focus of our review - 6)**

Thomas, D., Bostrom, R.
Building Trust and Cooperation through Technology Adaptation in Virtual Teams: Empirical Field Evidence 1
(2010) EDPACS, 42 (5), pp. 1-20.
https://www.scopus.com/inward/record.uri?eid=2-s2.0-84858982768&doi=10.1080%2f07366981.2010.537182&partnerID=40&md5=6b28a6a8af8096e4ef19d795d717fc48
DOCUMENT TYPE: Article
SOURCE: Scopus

**EXCLUDE (subject/population studied; not applicable or readily transferable to CCCs - 6)** Casey, V.
Developing trust in virtual software development teams
(2010) Journal of Theoretical and Applied Electronic Commerce Research, 5 (2), pp. 41-58.
https://www.scopus.com/inward/record.uri?eid=2-s2.0-77957981291&doi=10.4067%2fS0718-18762010000200004&partnerID=40&md5=8ba2feccb5d28472fc4e4ade2fb8d2ec
DOCUMENT TYPE: Article
SOURCE: Scopus

**EXCLUDE (subject/population studied; not applicable or readily transferable to CCCs - 6)**

Sillence, E.
Seeking out very like-minded others: Exploring trust and advice issues in an online health support group
(2010) International Journal of Web Based Communities, 6 (4), pp. 376-394.
https://www.scopus.com/inward/record.uri?eid=2-s2.0-78651579901&doi=10.1504%2fIJWBC.2010.035840&partnerID=40&md5=a8a0cae180bb40f002f88ffff30ced30
DOCUMENT TYPE: Article
SOURCE: Scopus

**EXCLUDE (subject/population studied; not applicable or readily transferable to CCCs - 4)** Parkins, J.R.
The problem with trust: Insights from advisory committees in the forest sector of Alberta
(2010) Society and Natural Resources, 23 (9), pp. 822-836.
https://www.scopus.com/inward/record.uri?eid=2-s2.0-77954734424&doi=10.1080%2f08941920802545792&partnerID=40&md5=4ee451b8f1b047ade4fb6e22c9e65ad9
DOCUMENT TYPE: Article
SOURCE: Scopus

**EXCLUDE (computer science focus; not applicable or readily transferable to CCCs - 6)**

Ballal, P., Lewis, F.L., Hudas, G.R.
Trust-based collaborative control for teams in communication networks
(2009) Studies in Computational Intelligence, 254, pp. 347-363.
https://www.scopus.com/inward/record.uri?eid=2-s2.0-78049306106&doi=10.1007%2f978-3-642-04227-0_11&partnerID=40&md5=84cb926e145bacd6b017ca876697efa9
DOCUMENT TYPE: Article
SOURCE: Scopus

**EXCLUDE (mobile banking satisfaction not relevant to our review - 6)**

Chung, N., Kwon, S.J.
Effect of trust level on mobile banking satisfaction: A multi-group analysis of information system success instruments
(2009) Behaviour and Information Technology, 28 (6), pp. 549-562.
https://www.scopus.com/inward/record.uri?eid=2-s2.0-77649334172&doi=10.1080%2f01449290802506562&partnerID=40&md5=151106213179989cabf5aecb4572e374
DOCUMENT TYPE: Article
SOURCE: Scopus

**EXCLUDE (subject/population studied; not applicable or readily transferable to CCCs - 3)** Indiramma, M., Anandakumar, K.R.
Behavioral analysis of team members in Virtual Organization based on Trust dimension and learning
(2009) World Academy of Science, Engineering and Technology, 39, pp. 269-274.
https://www.scopus.com/inward/record.uri?eid=2-s2.0-78651559455&partnerID=40&md5=03a112304ed3e8d55781162edff0d071
DOCUMENT TYPE: Article
SOURCE: Scopus

**EXCLUDE (technology adaptation in the setting of virtual teams not the focus of our review - 6)**

Thomas, D., Bostrom, R.
Building trust and cooperation through technology adaptation in virtual teams: Empirical field evidence
(2008) Information Systems Management, 25 (1), pp. 45-56.
https://www.scopus.com/inward/record.uri?eid=2-s2.0-38949127658&doi=10.1080%2f10580530701777149&partnerID=40&md5=a133b8b3e5d64fa269c88fd96cc4f2a3
DOCUMENT TYPE: Article
SOURCE: Scopus

**EXCLUDE (global software teams not the focus of our review - 6)**

Moe, N.B., Smite, D.
Understanding a lack of trust in global software teams: A multiple-case study
(2008) Software Process Improvement and Practice, 13 (3), pp. 217-231.
https://www.scopus.com/inward/record.uri?eid=2-s2.0-47749090951&doi=10.1002%2fspip.378&partnerID=40&md5=022806d0ab6a47e444b5416f62d56a65
DOCUMENT TYPE: Article
SOURCE: Scopus

**EXCLUDE (subject/population studied; technology adaptation in the setting of virtual teams not the focus of our review - 4)**

Ranco, D.J.
The trust responsibility and limited sovereignty: What can environmental justice groups Learn from Indian Nations?
(2008) Society and Natural Resources, 21 (4), pp. 354-362.
https://www.scopus.com/inward/record.uri?eid=2-s2.0-41449107333&doi=10.1080%2f08941920701329710&partnerID=40&md5=a5e024acd5bd066cfc24c5f71eb1e029
DOCUMENT TYPE: Review
SOURCE: Scopus

**EXCLUDE (virtual team collaboration in the setting of software outsourcing not the focus of our review - 6)**

Siakas, K.V., Siakas, E.
The need for trust relationships to enable successful virtual team collaboration in software outsourcing
(2008) International Journal of Technology, Policy and Management, 8 (1), pp. 59-75.
https://www.scopus.com/inward/record.uri?eid=2-s2.0-38049039998&doi=10.1504%2fIJTPM.2008.016181&partnerID=40&md5=84be94a2e6eb25ef24d4b2e2653a4475
DOCUMENT TYPE: Article
SOURCE: Scopus

**EXCLUDE (politics; not applicable or readily transferable to CCCs - 2)**

Font, J., Blanco, I.
Procedural legitimacy and political trust: The case of citizen juries in Spain
(2007) European Journal of Political Research, 46 (4), pp. 557-589.
https://www.scopus.com/inward/record.uri?eid=2-s2.0-34247342854&doi=10.1111%2fj.1475-6765.2007.00701.x&partnerID=40&md5=808322509a6a02ec86fe9612aa5b4b0d
DOCUMENT TYPE: Article
SOURCE: Scopus

**EXCLUDE (subject/population studied; not applicable or readily transferable to CCCs - 6)** Uden, L., Naaranoja, M.
The development of online trust among construction teams in Finland
(2007) Electronic Journal of Information Technology in Construction, 12, pp. 305-321.
https://www.scopus.com/inward/record.uri?eid=2-s2.0-34147151000&partnerID=40&md5=6f1c8ff771e3fd97193f7bf48ab646c9
DOCUMENT TYPE: Article
SOURCE: Scopus

**EXCLUDE (“trust” is used in a different context here than the focus of our review - 1)** Coupland, C.A.C., Savelyich, B.S.P., Hippisley-Cox, J., Kendrick, D., Groom, L., Cross, E.
A randomized controlled trial of the effect of providing information on accidental injury admissions and their costs to Primary Care Groups and Trusts
(2005) Family Practice, 22 (3), pp. 249-252.
https://www.scopus.com/inward/record.uri?eid=2-s2.0-19744369129&doi=10.1093%2ffampra%2fcmi016&partnerID=40&md5=0b9dea1d534556b194705a8e80135122
DOCUMENT TYPE: Article
SOURCE: Scopus

**EXCLUDE (“trust” is used in a different context here than the focus of our review - 1)**

Locock, L., Regen, E., Goodwin, N.
Managing or managed? Experience of general practitioners in English Primary Care Groups and Trusts
(2004) Health Services Management Research, 17 (1), pp. 24-35.
https://www.scopus.com/inward/record.uri?eid=2-s2.0-1542361221&doi=10.1258%2f095148404322772705&partnerID=40&md5=f916f2e35c582362ad36d784fc702dda
DOCUMENT TYPE: Article
SOURCE: Scopus

**EXCLUDE (subject not appropriate for our review, this is likely a commentary article as well - 6)**

Shell's bid to rebuild its reputation: Group Director Paul Skinner discusses the need for trust
(2003) Strategic Direction, 19 (7), pp. 9-11.
https://www.scopus.com/inward/record.uri?eid=2-s2.0-85001147083&doi=10.1108%2f02580540310794741&partnerID=40&md5=1e9a943e1e931f80262ca84c94f03f57
DOCUMENT TYPE: Article
SOURCE: Scopus

**EXCLUDE (subject/population studied; not applicable or readily transferable to CCCs - 2)** Haddad, L., Maluccio, J.A.
Trust, membership in groups, and household welfare: Evidence from KwaZulu-Natal, South Africa
(2003) Economic Development and Cultural Change, 51 (3), pp. 573-601.
https://www.scopus.com/inward/record.uri?eid=2-s2.0-0042230528&doi=10.1086%2f374986&partnerID=40&md5=a0c9fb1bbb9747c0ceb01ef08db968fa
DOCUMENT TYPE: Article
SOURCE: Scopus

**EXCLUDE (“trust” is used in a different context here than the focus of our review - 1)**

Kendrick, D., Groom, L., Hippisley-Cox, J., Savelyich, B.S.P., Webber, E., Coupland, C.
Accidental injury: A neglected area within Primary Care Groups and Trusts?
(2003) Health Education Research, 18 (3), pp. 380-388.
https://www.scopus.com/inward/record.uri?eid=2-s2.0-0037563984&doi=10.1093%2fher%2fcyf028&partnerID=40&md5=1fd026c7c50c902f2431606d43ab4778
DOCUMENT TYPE: Review
SOURCE: Scopus

**EXCLUDE (“trust” is used in a different context here than the focus of our review - 1)**

Hartin, J., Cook, J., Phillips, E., Singer, M., Webb, A., Adam, S.K.
Using algorithms in critical care outreach: UCLH Trust Patient Emergency Response Team (PERT) algorithm for heart rate > 125
(2002) Care of the Critically Ill, 18 (5), pp. 158-159.
https://www.scopus.com/inward/record.uri?eid=2-s2.0-0036792379&partnerID=40&md5=57678667f2d748823daacf3b7ba0aa45
DOCUMENT TYPE: Article
SOURCE: Scopus

**EXCLUDE (“trust” is used in a different context here than the focus of our review - 1)**

Rogers, A., Campbell, S., Coleman, A., Gask, L.
Implementing the mental health National Service Framework in primary groups and trusts
(2002) Primary Care Psychiatry, 8 (1), pp. 17-20.
https://www.scopus.com/inward/record.uri?eid=2-s2.0-0036271844&doi=10.1185%2f135525702125000417&partnerID=40&md5=95ab75e7f67a4b738a1815ec071e6fb1
DOCUMENT TYPE: Article
SOURCE: Scopus

**EXCLUDE (“trust” is used in a different context here than the focus of our review - 1)**

Rogers, A., Campbell, S., Gask, L., Sheaff, R., Marshall, M., Halliwell, S., Pickard, S.
Some National Service Frameworks are more equal than others: Implementing clinical governance for mental health in primary care groups and trusts
(2002) Journal of Mental Health, 11 (2), pp. 199-212.
https://www.scopus.com/inward/record.uri?eid=2-s2.0-0036252985&doi=10.1080%2f09638230020023589&partnerID=40&md5=aa92b8ef188ffc7b9d64c0c0ae91c1f3
DOCUMENT TYPE: Article
SOURCE: Scopus

**EXCLUDE (“trust” is used in a different context here than the focus of our review - 1)** Campbell, S.M., Sheaff, R., Sibbald, B., Marshall, M.N., Pickard, S., Gask, L., Halliwell, S., Rogers, A., Roland, M.O.
Implementing clinical governance in English primary care groups/trusts: Reconciling quality improvement and quality assurance
(2002) Quality and Safety in Health Care, 11 (1), pp. 9-14.
https://www.scopus.com/inward/record.uri?eid=2-s2.0-0036489351&partnerID=40&md5=46d8c0685e68a98a86f7669b1424e514
DOCUMENT TYPE: Article
SOURCE: Scopus

**EXCLUDE (“trust” is used in a different context here than the focus of our review - 1)**

Craig, G.
Primary care trust and local health group executive committee pharmacists: The story so far
(2002) Pharmaceutical Journal, 268 (7181), pp. 59-60.
https://www.scopus.com/inward/record.uri?eid=2-s2.0-0037132965&partnerID=40&md5=83d1d4411792084b05d8285f8bd002da
DOCUMENT TYPE: Article
SOURCE: Scopus

**EXCLUDE (“trust” is used in a different context here than the focus of our review - 1)**

Alborz, A., Wilkin, D., Smith, K.
Are primary care groups and trusts consulting local communities?
(2002) Health and Social Care in the Community, 10 (1), pp. 20-27.
https://www.scopus.com/inward/record.uri?eid=2-s2.0-0036359566&doi=10.1046%2fj.0966-0410.2001.00338.x&partnerID=40&md5=b0b7ddfb0eadc8a3611768d5ce47af1d
DOCUMENT TYPE: Review
SOURCE: Scopus

**EXCLUDE (population studied; not applicable or readily transferable to CCCs - 3)**

Tyler, T.R.
Public trust and confidence in legal authorities: What do majority and minority group members want from the law and legal institutions?
(2001) Behavioral Sciences and the Law, 19 (2), pp. 215-235.
https://www.scopus.com/inward/record.uri?eid=2-s2.0-0034963434&doi=10.1002%2fbsl.438&partnerID=40&md5=56e2057b0c195a2bde2487f4dcbc0aba
DOCUMENT TYPE: Review
SOURCE: Scopus

**EXCLUDE (“trust” is used in a different context here than the focus of our review - 1)** Banks-Smith, J., Dowswell, T., Gillam, S., Shipman, C.
Primary care groups and trusts.
(2001) Nursing times, 97 (45), pp. 30-32.
https://www.scopus.com/inward/record.uri?eid=2-s2.0-0035829863&partnerID=40&md5=7fff3ca38a18937366347ab54d0cb88c
DOCUMENT TYPE: Article
SOURCE: Scopus

**EXCLUDE (“trust” is used in a different context here than the focus of our review - 1)**

Wilkin, D., Coleman, A.
From primary care groups to primary care trusts in the new NHS in England
(2001) Primary Health Care Research and Development, 2 (4), pp. 215-222.
https://www.scopus.com/inward/record.uri?eid=2-s2.0-84993069797&doi=10.1191%2f146342301682157692&partnerID=40&md5=2fb4d617f3cae11ccec7e2133736455c
DOCUMENT TYPE: Article
SOURCE: Scopus

**EXCLUDE (“trust” is used in a different context here than the focus of our review - 1)**

Kerr, R.
Group health: avoiding a MET (multiple-employer trust) crisis.
(1989) Association management, 41 (4), pp. 82-85.
https://www.scopus.com/inward/record.uri?eid=2-s2.0-0024653216&partnerID=40&md5=7566ea3c3dcb09b2cc72aee7e1a2d6fa
DOCUMENT TYPE: Article
SOURCE: Scopus

**EXCLUDE (politics; not applicable or readily transferable to CCCs - 3)**

Pierce, J.C., Lovrich, N.P., Jr.
TRUST IN THE TECHNICAL INFORMATION PROVIDED BY INTEREST GROUPS: THE VIEWS OF LEGISLATORS, ACTIVISTS, EXPERTS, AND THE GENERAL PUBLIC
(1983) Policy Studies Journal, 11 (4), pp. 626-639.
https://www.scopus.com/inward/record.uri?eid=2-s2.0-0021043367&doi=10.1111%2fj.1541-0072.1983.tb00566.x&partnerID=40&md5=89e74ae2c6e8374de9352317526dd485
DOCUMENT TYPE: Article
SOURCE: Scopus
